# LINC01929 promotes breast cancer progression through a TFRC-associated ferroptosis pathway

**DOI:** 10.1038/s41420-026-03248-y

**Published:** 2026-07-09

**Authors:** Gang Li, Zhijun Yu, Hongmei Xu, Xianglin Sun, Bing Wang, Tianjiao Wei, Yifei Liu, Lisha Ye, Hongmei Qiu, Guohua Wang

**Affiliations:** 1 Institute of Special Environmental Medicine and Affiliated Rehabilitation Hospital, Nantong University, Nantong, China; 2 Huzhou Health Vocational College, Huzhou, China; 3 Second People’s Hospital of Nantong, Nantong, China; 4 Department of pharmacy, Affiliated Hospital of Nantong University, Nantong, China; 5 Department of Pathology, Affiliated Hospital of Nantong University, Nantong, China; 6 Department of Respiration, Nantong Geriatric Rehabilitation Hospital and Branch of Affiliated Hospital of Nantong University, Nantong, China; 7 Xuanwu Hospital Capital Medical University, Beijing, China

**Keywords:** Breast cancer, Long non-coding RNAs

## Abstract

Breast cancer (BC) remains the most prevalent malignancy among women worldwide, with persistent challenges such as drug resistance and tumor progression despite significant therapeutic advancements. The roles of ferroptosis and long non-coding RNAs (lncRNAs) in BC are not yet fully elucidated, underscoring the need for novel therapeutic targets. In this study, we investigated the function of LINC01929 in BC through a combination of in vitro and in vivo experiments, bioinformatics analysis, RNA pull-down mass spectrometry, and clinical tissue validation. We found that LINC01929 is significantly overexpressed in BC tissues and is associated with poor patient prognosis. Mechanistically, LINC01929 promoted malignant phenotypes in BC cells and was associated with reduced ferroptosis-related stress, with these effects being at least partly dependent on transferrin receptor (TFRC). Conversely, silencing LINC01929 led to reduced TFRC expression and induced ferroptosis in BC cells. Clinical data further confirmed that elevated TFRC levels correlate with aggressive tumor features and unfavorable outcomes. These findings suggest that targeting LINC01929 to regulate TFRC-mediated ferroptosis could represent a promising therapeutic strategy for BC, positioning both LINC01929 and TFRC as potential biomarkers and therapeutic targets.

**Figure:**
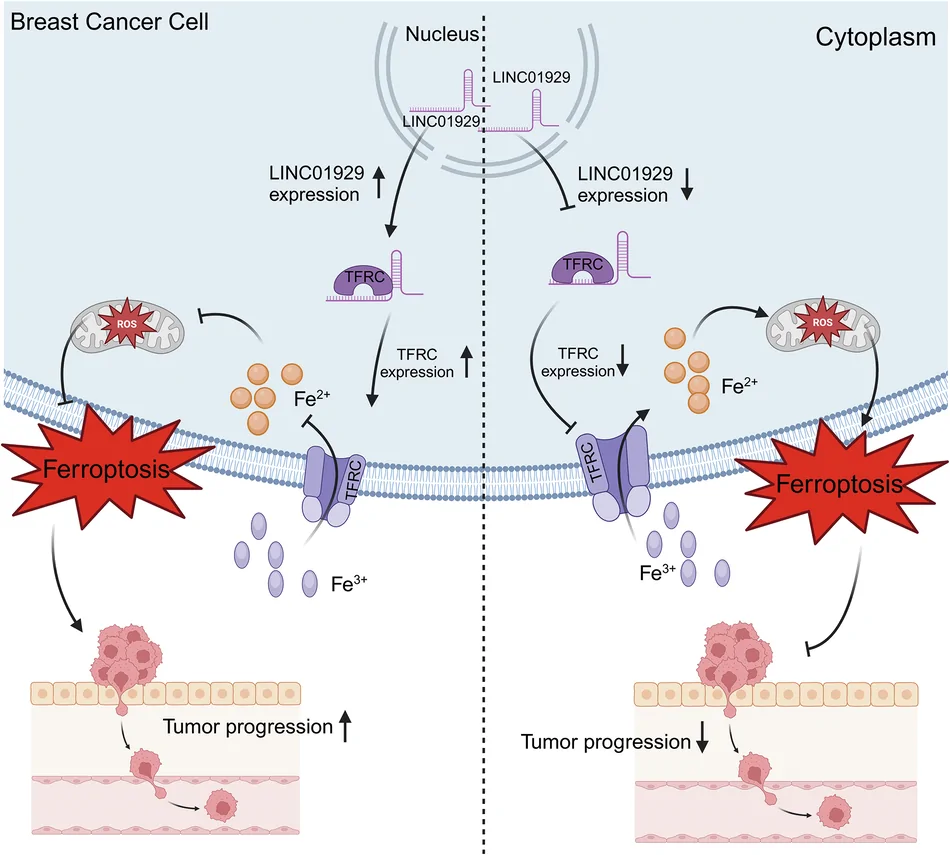

## Introduction

According to the latest global cancer statistics from the International Agency for Research on Cancer (IARC), a total of 2.34 million new cases of breast cancer (BC) were reported worldwide in 2022 [1]. BC is the most prevalent malignancy among women worldwide and remains a leading cause of cancer-related death [1]. Recent statistical data indicate a diminished rate of improvement in BC survival outcomes, implying a potential stagnation in therapeutic advancements [2]. Effective cancer treatment depends on early detection and precise intervention, both of which require a comprehensive understanding of disease pathogenesis. The oncogenic mechanisms underlying BC are highly complex, involving endocrine signaling, genetic alterations, and epigenetic modifications [3]. Consequently, a deeper understanding of the molecular mechanisms driving BC progression and the discovery of new therapeutic targets are critical to enhancing therapeutic outcomes.

Within the human genome, the preponderance of sequences is transcribed into RNA; however, only approximately 3% are transcribed into messenger RNA (mRNA), which encodes proteins [4]. Most transcripts are non-coding RNAs (ncRNAs) that lack protein-coding functions but perform crucial functions in regulating the progression and dissemination of several cancer types [5]. A non-coding RNA longer than 200 nucleotides is known as long non-coding RNA (lncRNA), and it has been linked to the development of bladder, breast, and liver cancers [6–8]. Growing data underscores the substantial function of lncRNAs in malignancies, where they act as oncogenes or inhibitors of tumor growth, exhibiting complex and precise regulatory effects on cancer development and progression across different tumor types, especially in BC [9, 10]. LncRNAs are stably expressed in tissue and plasma, with specific expression patterns in tumors, exhibiting tissue specificity and multifaceted biological functions. These functions include epigenetic regulation, gene expression control, nuclear chromatin modification, X-chromosome inactivation, imprinting, nucleocytoplasmic transport, DNA methylation, RNA splicing, and translational regulation. LncRNAs are also implicated in cancer invasion and metastasis and serve as potential diagnostic and prognostic markers [11, 12]. Thus, elucidating the mechanisms by which lncRNAs influence BC is crucial for validating their utility as novel indicators and therapeutic targets in BC prognosis and treatment. In our previous study, Gene Expression Omnibus (GEO) and Cancer Genome Atlas (TCGA) databases were searched for lncRNAs related to triple negative BC, and LINC01929 was found to be highly expressed in BC. The expression and precise mechanism of LINC01929 in BC have not been investigated, despite the fact that related research on the gene mostly focused on bladder cancer, non-small cell lung cancer, and oral squamous cell carcinoma [13, 14].

Ferroptosis is a newly discovered kind of controlled cell death, distinct from apoptosis, autophagy, and necroptosis, and is characterized by iron gathering, oxidation of lipids, and a decrease in the antioxidant glutathione (GSH) levels [15]. This process is primarily driven by mitochondrial lipid peroxidation resulting from the buildup of iron-dependent reactive oxygen species (ROS). In the last few years, the activation of ferroptosis has become a viable therapeutic approach, particularly for cancers that are resistant to traditional therapies [16]. Ferroptosis has been demonstrated to be either promoted or inhibited by a variety of tumor suppressor and carcinogenic signaling pathways. Additionally, certain tumor cells have a distinct dependence on defensive mechanisms that are dependent on ferroptosis in order to withstand oxidative and metabolic stress [17–19]. Recently, ferroptosis has been implicated in numerous tumor-related pathophysiological events, including immunological response, aging, and the development and spread of tumors [20–22]. Consequently, investigating ferroptosis-related genes linked to prognosis may yield important information for BC tailored treatments. More importantly, in our study, the proteins interacting with LINC01929 were found to be involved in ferroptosis pathway in pathway enrichment analysis by RNA pull down assay, suggesting a previously unrecognized association between LINC01929 and ferroptosis in BC.

In summary, although LINC01929 has been identified as a highly expressed lncRNA in BC through bioinformatics analyses, its precise biological functions and underlying mechanisms remain largely unexplored. Meanwhile, growing interest in ferroptosis—a distinct, iron-dependent form of regulated cell death—has underscored its therapeutic potential, particularly in treatment-resistant cancers. This study identifies LINC01929 as a lncRNA associated with ferroptosis-related regulation in BC.

## Results

### Differentially expressed lncRNAs in BC and BC adjacent normal tissues

**Fig. 1:**
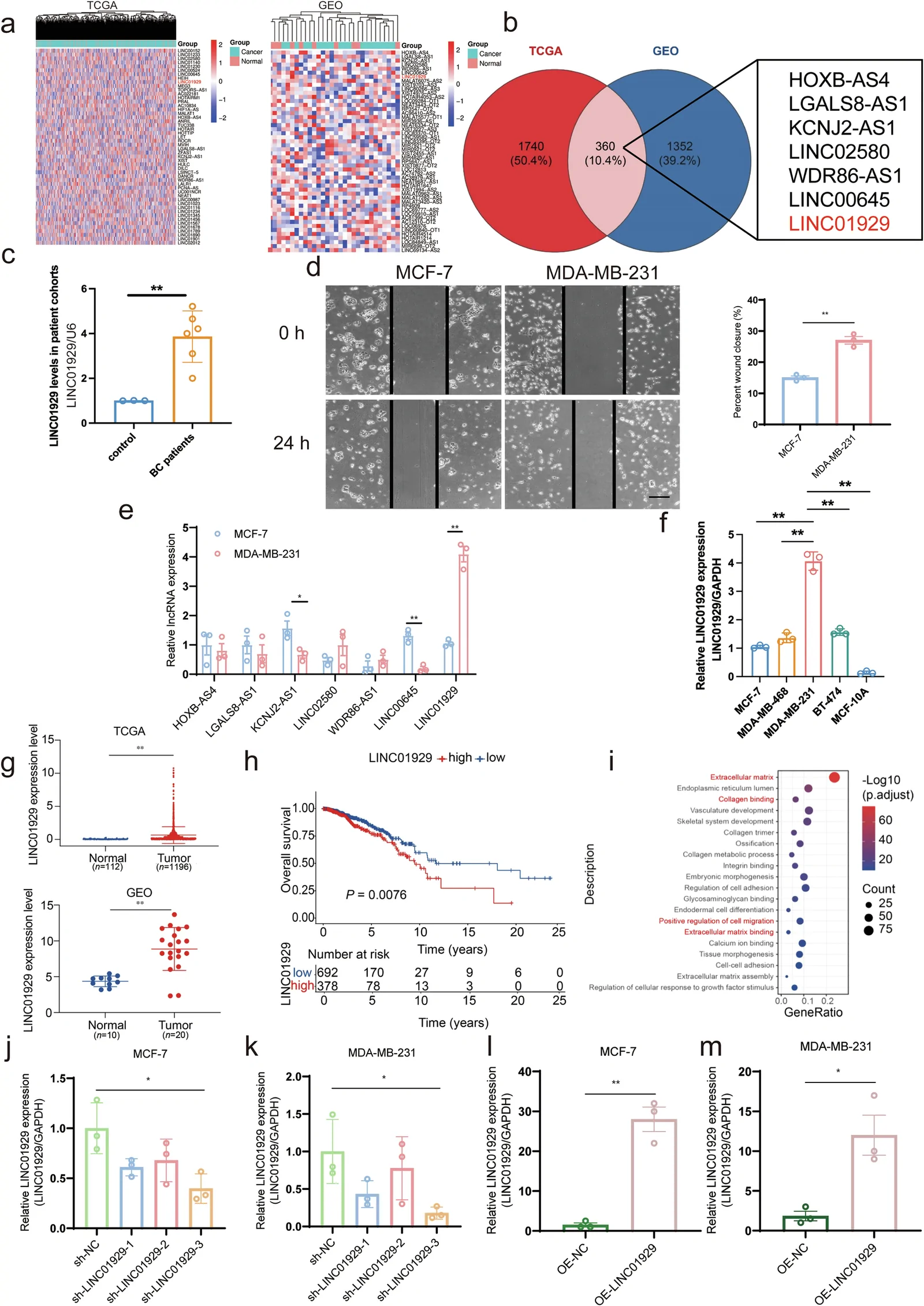
Differentially expressed lncRNAs in breast cancer (BC) and adjacent tissues. **a** Heatmap showing 47 lncRNAs with significantly different expression levels between BC tissues and neighboring normal tissues from the Cancer Genome Atlas (TCGA) and Gene Expression Omnibus (GEO) (GSE119233) datasets. Red indicates elevated expression, whilst blue signifies diminished expression. **b** Venn diagram showing 360 differentially expressed lncRNAs common to both the TCGA and GSE119233 datasets. **c** qRT-PCR was performed to determine LINC01929 expression in peripheral blood samples from BC patients (*n* = 6) and healthy controls (*n* = 3). U6 was used as an internal control. **d** Representative images of wound healing in MCF-7 and MDA-MB-231 cells at 24 hours. Scale bar = 200 μm. Quant analysis of wound healing efficacy in MCF-7 and MDA-MB-231 cells (*n* = 3). **e** Mathematical graphs from quantitative Real-Time Polymerase Chain Reaction (qRT-PCR) illustrating comparative variations in kinds of lncRNA expression in MCF-7 and MDA-MB-231 cells with distinct capacities. **f** qRT-PCR evaluation of LINC01929 expression in BC cell lines (MCF-7, MDA-MB-468, MDA-MB-231, BT-474 and MCF-10A) (*n* = 3) **g** LINC01929 expression levels in BC tissues compared to adjacent tissues, as shown in the TCGA and GEO (GSE119233) databases. **h** Kaplan–Meier survival study showing that elevated LINC01929 expression correlates with reduced long-term survival in BC patients from the TCGA dataset (*n* = 1070). **i** GO analysis of genes associated with LINC01929 (*n* = 407) in the TCGA dataset. **j**–**m** qRT-PCR results showing LINC01929 expression in MCF-7 and MDA-MB-231 cells transformed with sh-NC, sh-LINC01929-1, sh-LINC01929-2, sh-LINC01929-3, an empty (OE-NC) vector, and OE-LINC01929 (*n* = 3). The sh-NC and sh-LINC01929 represent the control and knockdown groups, respectively, while empty vector (OE-NC) and OE-LINC01929 represent the control and overexpression groups. **P < 0.01, *P < 0.05.

To identify differentially expressed lncRNAs between breast cancer tissues and adjacent normal tissues, we collected transcriptome count data of 1208 samples from the TCGA database, including 1096 breast cancer tissue samples and 112 adjacent normal tissue samples. After integration, a total of 14,142 lncRNAs were obtained. Using |Log_2_FC| > 1 and *P* adj < 0.05 as the screening criteria for differentially expressed lncRNAs in breast cancer, we identified 2100 significantly altered lncRNAs, among which 1075 were upregulated and 1025 were downregulated. The results were visualized as a heatmap (Fig. 1a). Meanwhile, we analyzed high-throughput sequencing data from the GEO dataset (GSE119233). Based on the same criteria (|Log_2_FC| > 1 and *P* adj < 0.05), a total of 4885 lncRNAs were detected, among which 1712 showed significant differential expression, including 887 upregulated and 825 downregulated lncRNAs. The results were displayed using a heatmap (Fig. 1a). To further identify lncRNAs consistently differentially expressed between breast cancer and adjacent normal tissues, we used a Venn diagram to determine the intersection of differentially expressed lncRNAs from the two datasets, revealing 360 lncRNAs that were significantly differentially expressed (*P* < 0.05) in both datasets (Fig. 1b). From the 360 differentially expressed lncRNAs, 7 lncRNAs (HOXB-AS4, LGALS8-AS1, KCNJ2-AS1, LINC02580, WDR86-AS1, LINC00645 and LINC01929) with the largest fold change were selected for qRT-PCR analysis (Figs. 1b and S1a Table S4). In addition, we conducted qRT-PCR analysis of peripheral blood samples from BC patients and healthy controls (Fig. 1c). The results revealed significantly elevated circulating LINC01929 levels in BC patients compared to healthy controls (*P* < 0.01), supporting its potential as a clinically accessible biomarker. Human BC cell lines MDA-MB-231 and MCF-7 exhibit different levels of invasiveness and malignancy, with MDA-MB-231 showing higher metastatic and malignant potential, as confirmed by wound-healing assays (Fig. 1d, *P* < 0.01). The examination of cell lines with differing migratory capacities demonstrated a considerable elevation in LINC01929 expression in MDA-MB-231 cells, whereas lncRNAs KCNJ2-AS1 and LINC00645 were significantly down-regulated in MCF-7 cells (Fig. 1e, *P* < 0.01). As shown in Fig. 1f, the investigation focused on the potential role of LINC01929 in BC, prompted by its elevated expression in highly metastatic malignant BC cell lines. Expression analysis of LINC01929 was conducted in BC cell lines, specifically MCF-10A, MCF-7, MDA-MB-468, MDA-MB-231 and BT-474 cells. qRT-PCR results revealed a progressive increase in LINC01929 expression with increasing metastatic capacity, showing the lowest levels in MCF-10A and MCF-7 cells and the highest levels in MDA-MB-231 and BT-474 cells (*P* < 0.01) (Fig. 1f). Consequently, we postulated that LINC01929 may be crucial for BC cell migration. MCF-7 and MDA-MB-231 cells, characterized by low and high LINC01929 expression, respectively, were chosen for subsequent functional experiments.

### LINC01929 is up-regulated in BC and promotes the migration of BC cells by interacting with proteins

Significantly higher levels of LINC01929 were found in BC tumor tissues, and the TCGA and GEO databases (GSE119233) showed the differences in LINC01929 levels between normal and tumor tissues (Fig. 1g, *P* < 0.01). From the 360 differentially expressed lncRNAs, 7 lncRNAs (HOXB-AS4, LGALS8-AS1, KCNJ2-AS1, LINC02580, WDR86-AS1, LINC00645 and LINC01929) with the largest fold change were selected for Kaplan-Meier (KM) analysis. These 7 lncRNAs were all associated with survival prognosis. In addition, BC patients with elevated LINC01929 expression experienced a worse outcome (Figs. S1b–g and 1h). GO enrichment analysis revealed that LINC01929 mostly participated in collagen binding, enhancement of cellular migration, and extracellular matrix binding (Fig. 1i). To investigate the biological role of LINC01929 in BC, three distinct LINC01929-targeting shRNA plasmids and one overexpression plasmid were engineered for transfection into MCF-7 and MDA-MB-231 cell lines. According to the results of qRT-PCR, sh-LINC01929-3 had the most potent inhibitory effect on both BC cell lines (Fig. 1j, k, *P* < 0.05). The LINC01929 overexpression vector was constructed using the pcDNA3.1 backbone, and transfection efficiency was assessed. In BC cells, LINC01929 expression was significantly upregulated following transfection with the overexpression vector (Fig. 1l, m, *P* < 0.05).

### LINC01929 promotes proliferation, migration and invasion of BC cells

**Fig. 2:**
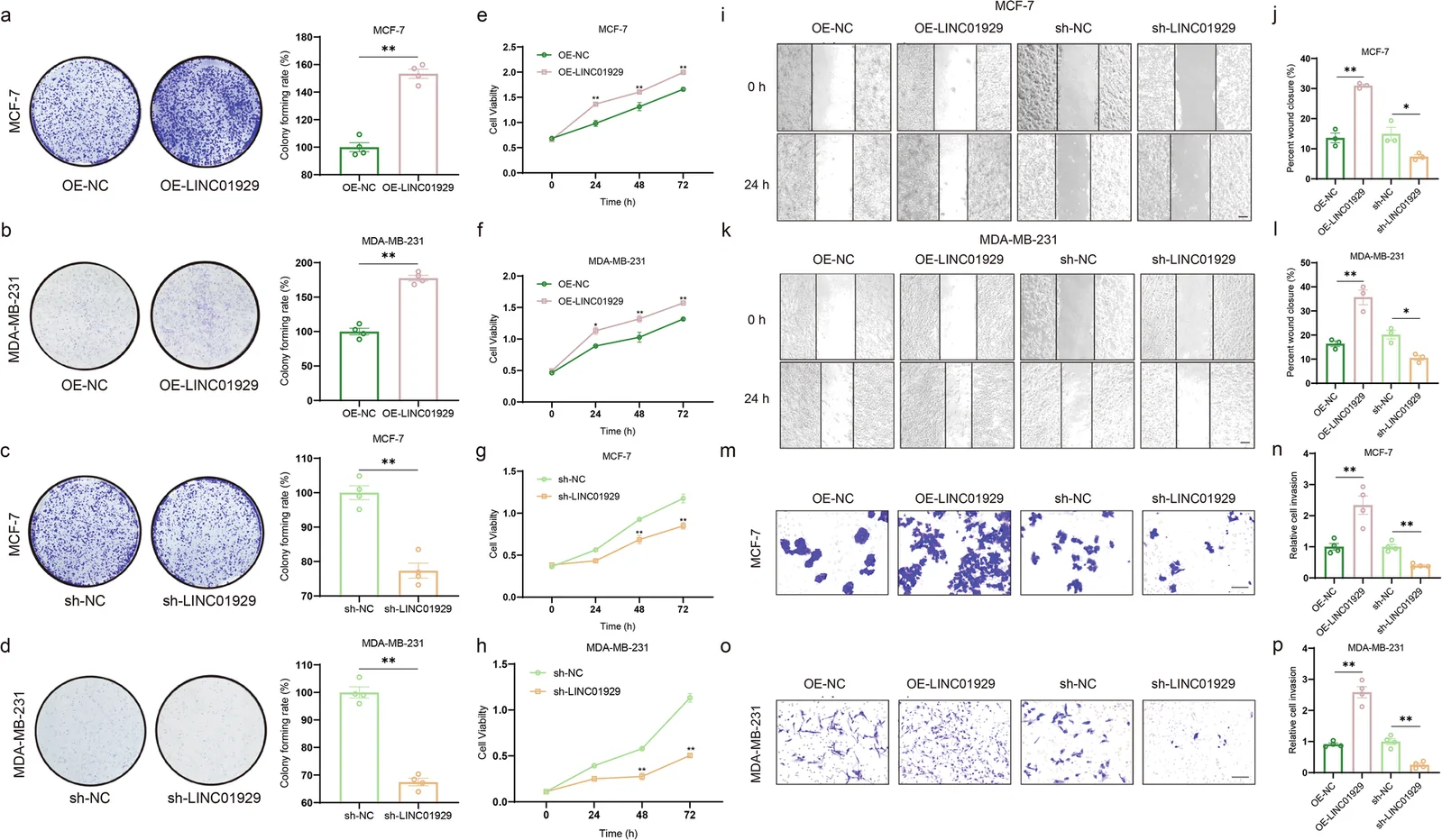
Effect of LINC01929 on proliferation, migration and invasion of BC cells in vitro. **a**–**d** Colony formation assays assessing the proliferation of MDA-MB-231 and MCF-7 cells following LINC01929 overexpression, LINC01929 suppressing and transfection using an empty vector. Statistical examination of the colony formation rate in MCF-7 and MDA-MB-231 cells transfected with OE-LINC01929 and sh-LINC01929 (*n* = 4). **e**–**h** Cell counting kit-8 (CCK-8) assays demonstrating the proliferation of MCF-7 and MDA-MB-231 cells transfected with OE-LINC01929 or sh-LINC01929 at 24, 48, and 72 h (*n* = 5). **i**–**l** Wound healing assays assessing migration in BC cell lines (MDA-MB-231, MCF-7) transfected with LINC01929 overexpression, LINC01929 interference, and evaluated at 24 h. Scale bar = 200 μm. The statistical evaluation of wound healing ability is shown (*n* = 3). **m**–**p** Transwell was used to evaluate the invasive potential of MDA-MB-231 and MCF-7 cells with LINC01929 overexpression, knockdown, and empty vector transfection at 24 h. Scale bar = 100 μm. The statistical examination of cellular invasion capability is also presented (*n* = 3). The sh-NC and sh-LINC01929 groups represent the control and knockdown groups, respectively, while the empty vector and OE-LINC01929 represent the control and overexpression groups. *P < 0.05; **P < 0.01.

Clone formation assays were conducted to assess changes in the proliferation of MCF-7 and MDA-MB-231 cells (Fig. 2a-d). BC cells with increased LINC01929 expression displayed a higher capacity for colony formation in contrast to the empty vector group. In contrast, as compared to the empty vector cohorts, LINC01929 inhibition led to a substantial reduction in both colony size and quantity in MCF-7 and MDA-MB-231 cells (*P* < 0.01). The CCK-8 results also showed that after OE-LINC01929 transfection, BC cell proliferation rates rose in comparison to the empty vector group, whereas LINC01929 interference markedly decreased multiplication in MCF-7 and MDA-MB-231 cells within 72 h (Fig. 2e–h, *P* < 0.01). The wound-healing assay showed that after LINC01929 overexpression, the capacity of BC cells to migrate was significantly increased. Conversely, within 24 hours, BC cells transfected with sh-LINC01929 showed noticeably less capacity to migrate than the empty vector group (Fig. 2i–l, *P* < 0.05). The transwell assay revealed that LINC01929 overexpression enhanced the invasive capacity of MCF-7 and MDA-MB-231 cells, while its silencing led to a marked reduction compared to the control group (Fig. 2m–p, *P* < 0.01).

### LINC01929 promotes BC progression by enhancing the growth of subcutaneous xenograft tumors

**Fig. 3:**
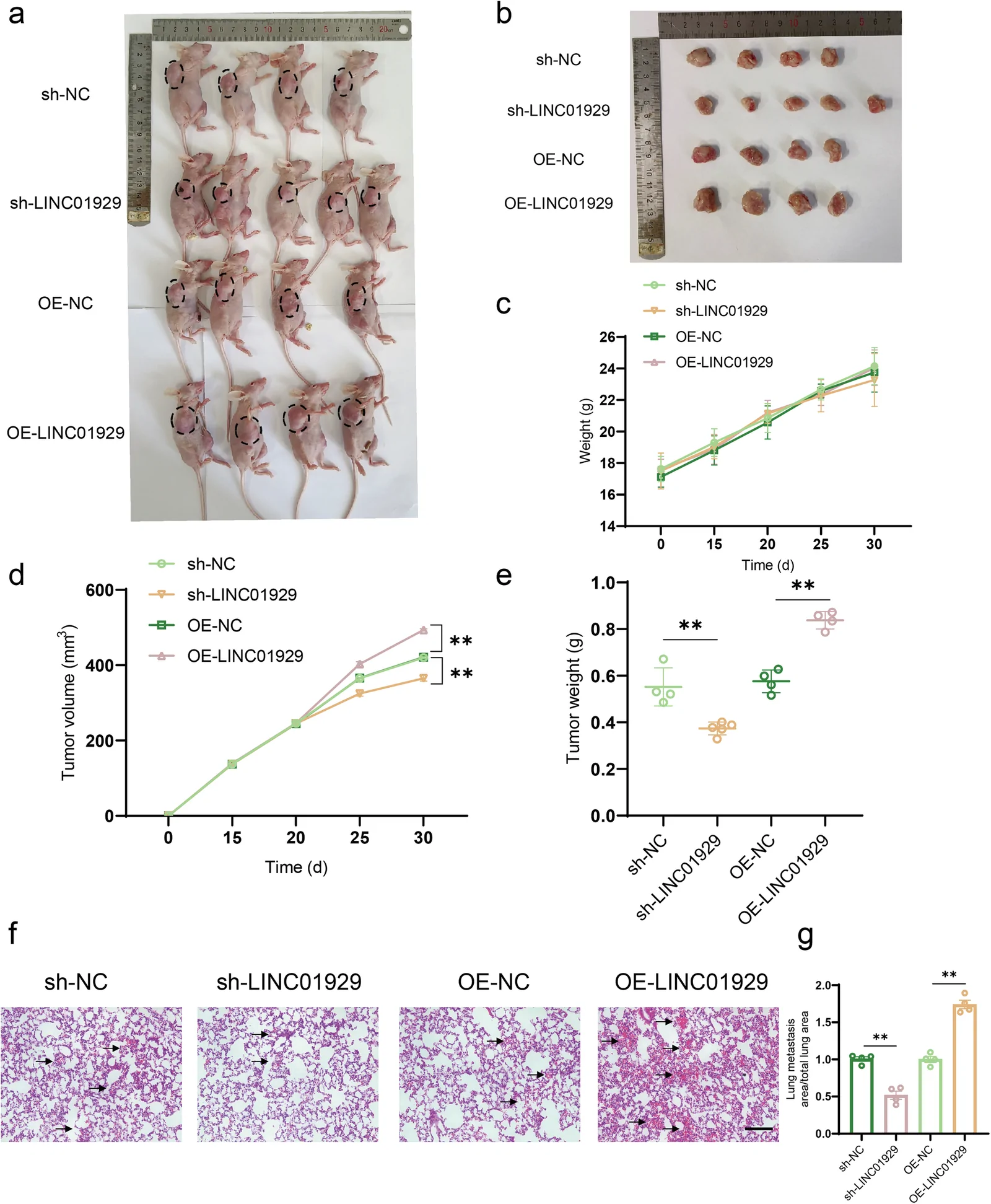
LINC01929 influences BC progression by regulating the growth of subcutaneous xenograft tumor. **a**, **b** MDA-MB-231 cells transfected with either the LINC01929 overexpression vector, empty vector, LINC01929 interference vector, or control vector were subcutaneously administered to nude mice to create a tumor model. Photographs of tumors were taken 30 days post-injection. (**c**) Body weight changes in nude mice over a 30-day period (*n* = 4-5). **d**, **e** Growth curves of tumor volume and comparisons of tumor weights across different groups (*n* = 4-5). **f**, **g** Representative hematoxylin and eosin (H&E) stained images of mouse lung tissue, showing the ratio of lung metastatic area to entire lung area in each group (*n* = 4). Arrows indicate metastatic sites in the lung tissue. *P < 0.05; **P < 0.01.

To conduct a more thorough examination of the function of LINC01929 in vivo, MDA-MB-231 cells expressing LINC01929 or a control vector were subcutaneously injected into nude mice, successfully establishing subcutaneous tumors (Fig. 3a). At day 30, tumor formation was observed in mice in both the control and overexpression groups, with all tumor-bearing mice surviving until the end of the experiment (Fig. 3b). The tumor weight, body weight, and tumor volume were tracked (Fig. 3c–e). The tumor development curve revealed that the OE-LINC01929 group grew at a substantially faster pace than the OE-NC group, but the sh-LINC01929 group grew at a slower rate (Fig. 3d, *P* < 0.01). Additionally, compared to the control mice, the tumor weight and volume were considerably smaller in the sh-LINC01929 group and much larger in the OE-LINC01929 group (Fig. 3e, *P* < 0.01). To further assess the impact of LINC01929 on BC metastasis in vivo, lung tissue was selected due to its ease of observation. Compared with the OE-NC group, the LINC01929 overexpression group exhibited a greater number and larger area of metastatic lung nodules, whereas the knockdown group showed the opposite effect (Fig. 3f, g, *P* < 0.01).

### LINC01929 promotes tumor progression by inhibiting ferroptosis in BC cells

**Fig. 4:**
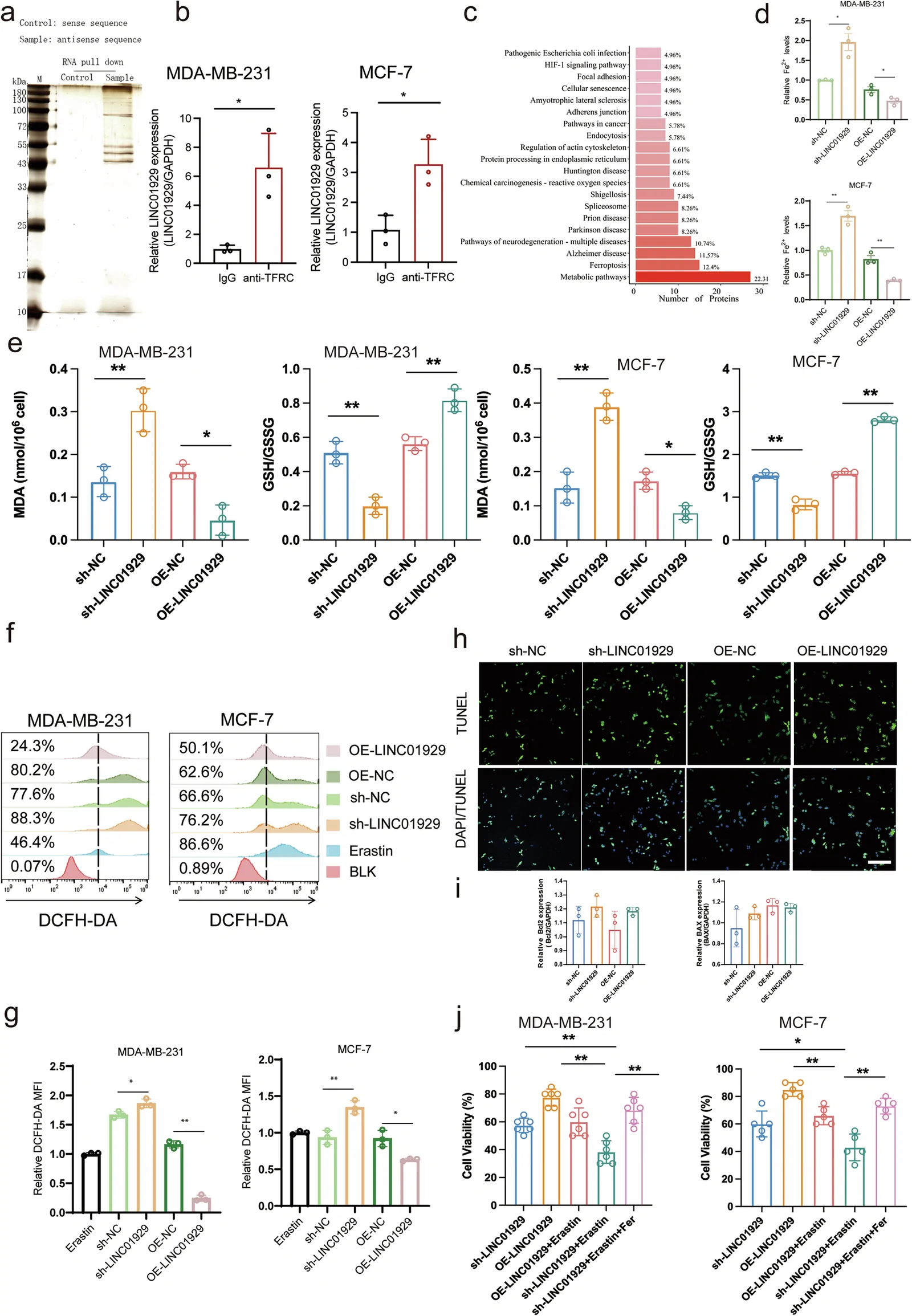
LINC01929 promotes tumor progression by inhibiting ferroptosis in BC cells. **a** RNA pull-down assay was used to screen the binding proteins of LINC01929. **b** RNA immunoprecipitation (RIP) assays were performed to investigate the interaction between LINC01929 and TFRC in MCF-7 and MDA-MB-231 cells. Cells were lysed and immunoprecipitated with anti-TFRC antibody or IgG control. The co-precipitated RNA was extracted and analyzed by RT-qPCR to measure the enrichment of LINC01929 (*n* = 3). **c** KEGG enrichment analyses of proteins that bind found using RNA pull-down followed by mass spectrometry (MS). **d** Measurement of Fe²⁺ levels in MCF-7 and MDA-MB-231 cells transfected with LINC01929 overexpression or silencing vectors using a ferrous ion assay kit (*n* = 3). **e** Quantification of malondialdehyde (MDA) and GSH/GSSG ratio in the indicated groups (*n* = 3). **f**, **g** Flow cytometry analysis of ROS levels in MCF-7 and MDA-MB-231 cells subsequent to LINC01929 upregulation or reduction, with Erastin, a ferroptosis inducer, used as a positive control (*n* = 3). **h** Apoptosis in MDA-MB-231 cells was assessed by Terminal deoxynucleotidyl transferase dUTP nick end labeling (TUNEL) staining 48 h after plasmid transfection. TUNEL staining showed no difference in the number of TUNEL-positive cells following LINC01929 knockdown or overexpression. Scale bar, 200 μm. **i** Relative mRNA expression levels of BAX and BCL2 were measured by qRT-PCR (normalized to GAPDH). (*n* = 3). **j** CCK-8 cell viability assay showing that the ferroptosis inhibitor Ferrostatin-1 significantly rescued cell viability in sh-LINC01929 cells (*n* = 5–6). The sh-NC and sh-LINC01929 groups represent the control and knockdown groups, respectively, while the empty vector and OE-LINC01929 represent the control and overexpression groups. *P < 0.05; **P < 0.01.

To further elucidate the precise mechanism by which LINC01929 facilitates BC invasion and metastasis, we first screened out 157 differentially expressed proteins(DEPs) that interacted with LINC01929 by RNA pull down MS assay (Fig. 4a). To investigate the interaction between LINC01929 and TFRC, RIP assays were performed. As shown in Fig. 4b, LINC01929 was significantly enriched in the anti-TFRC group compared to the IgG control in both MCF-7 and MDA-MB-231 cells (Fig. 4b, *P* < 0.01). These results demonstrate that LINC01929 directly interacts with TFRC in BC cells. In addition, the 157 DEPs were then subjected to KEGG enrichment analysis, and ferroptosis was found to be the only statistically significant cell death-related pathway in KEGG enrichment analysis (Fig. 4c).To test whether LINC01929 induces ferroptosis in BC cells, ferrous iron concentration was measured and found to be higher in sh-LINC01929 group, indicating that LINC01929 silencing promotes ferroptosis in MDA-MB-231 and MCF-7 cells (Fig. 4d, *P* < 0.05). Furthermore, we conducted our ferroptosis assays to include malondialdehyde (MDA) levels and the GSH/GSSG ratio as additional biochemical indicators of lipid peroxidation and redox imbalance in MDA-MB-231 and MCF-7 cells (Fig. 4e, *P* < 0.05). MDA levels were significantly increased, and the GSH/GSSG ratio was markedly decreased in the sh-LINC01929 group compared with controls, whereas OE-LINC01929 cells exhibited the opposite trend, consistent with a ferroptosis-suppressive role of LINC01929 (Fig. 4e). To assess lipid peroxidation levels, we used flow cytometry after inducing ferroptosis with Erastin as a positive control, followed by LINC01929 silencing. ROS accumulation was increased in sh-LINC01929 cells and remained comparable to or greater than that observed in the erastin-treated positive control, further supporting enhanced ferroptosis-related oxidative stress (Fig. 4f-g). These results suggest that LINC01929 promotes tumor progression by inhibiting ferroptosis in BC cells. Finally, we also performed TUNEL cell apoptosis assays and qRT-PCR analysis of apoptosis-related genes (BAX and BCL2) to determine whether LINC01929 regulates cell death through an apoptotic mechanism. As shown in Fig. 4h–i, there were no significant differences in the number of TUNEL-positive cells or in the mRNA expression levels of BAX and BCL2 among the groups, suggesting that LINC01929 does not induce typical apoptosis. To verify the specificity of ferroptosis, we additionally applied Ferrostatin-1 (ferroptosis inhibitor) (Fig. 4i). Ferrostatin-1 effectively rescued the loss of cell viability in sh-LINC01929 cells, confirming that the observed cell death was ferroptosis-specific (Fig. 4j, *P* < 0.01). In conclusion, LINC01929 promotes BC progression mainly by inhibiting cell ferroptosis.

### LINC01929 inhibits ferroptosis in BC cells by targeting TFRC to promote tumor progression

**Fig. 5:**
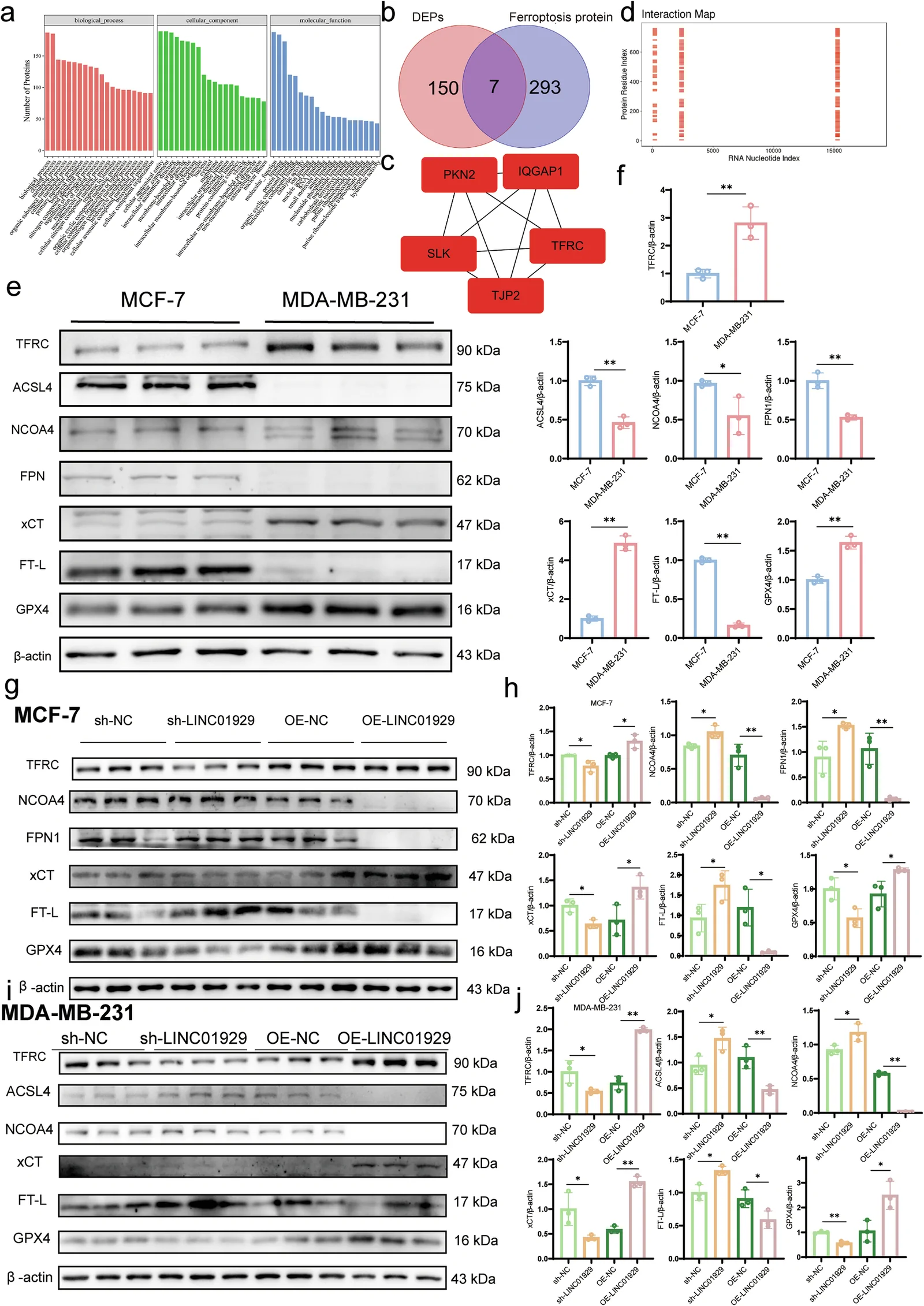
LINC01929 inhibits ferroptosis in BC cells by targeting TFRC to promote tumor progression. **a** GO enrichment analyses of proteins that bind found using RNA pull-down followed by mass spectrometry (MS). **b** Venn diagram showing 7 overlapping proteins identified as both binding and ferroptosis-related proteins isolated using RNA pull-down MS. **c** Key hub genes (*PNK2, IQGAP1, TFRC, TJP2, SLK*) were identified in the PPI network using CytoHubba in Cytoscape. **d** Predicted binding sites between LINC01929 and TFRC as determined by the catRAPID database. **e**, **f** Western blot analysis of TFRC and ferroptosis-related proteins (ACSL4, NCOA4, FPN1, xCT, FT-L and GPX4) in MCF-7 and MDA-MB-231 cell lines (*n* = 3). **g**–**j** Western blot analysis of TFRC and ferroptosis-related proteins (ACSL4, NCOA4, FPN1, xCT, FT-L and GPX4) expression following LINC01929 overexpression or silencing in MCF-7 and MDA-MB-231 cells, with empty vectors serving as controls (*n* = 3). The sh-NC and sh-LINC01929 groups represent the control and knockdown groups, respectively, while the empty vector and OE-LINC01929 represent the control and overexpression groups. *P < 0.05; **P < 0.01.

Then GO enrichment analysis of 157 DEPs demonstrated that they were predominantly associated with RNA binding and protein binding in molecular function (Fig. 5a). Therefore, we interposed these 157 DEPs with ferroptosis-related proteins, and found 7 proteins related to ferroptosis among the 157 DEPs (Fig. 5b). To find out which ferroptosis proteins LINC01929 interacted with, PPI network analysis was performed (Figure S2). The MCC algorithm in Cytoscape software was used to screen TFRC as the core node in the PPI network (Fig. 5c). To verify the potential interaction, we first used the catRAPID online database to predict the presence of binding sites between LINC01929 and TFRC (Fig. 5d and Table S5). Western blot analysis showed that TFRC levels were markedly increased in MDA-MB-231 cells compared to MCF-7 cells (Fig. 5e, f; *P* < 0.01), mirroring the expression pattern of LINC01929, and indicating a positive correlation between the two. Furthermore, LINC01929 overexpression led to a significant upregulation of TFRC protein levels, while silencing LINC01929 resulted in a marked reduction of TFRC expression in MCF-7 and MDA-MB-231 cells (Fig. 5g–j, *P* < 0.05). To further clarify the regulatory relationship between LINC01929 and TFRC, we co-transfected cells with LINC01929 siRNA and a TFRC overexpression plasmid. qRT-PCR and Western blot analyses revealed that TFRC overexpression could partially reverse the effects of LINC01929 silencing, confirming their functional linkage (Fig. S3a, b, *P* < 0.05). Western blot and qRT-PCR analyses confirmed that LINC01929 positively regulates TFRC expression in both MCF-7 and MDA-MB-231 cells. TFRC overexpression markedly increased TFRC protein levels, whereas LINC01929 knockdown significantly decreased TFRC expression. Importantly, TFRC overexpression reversed the reduction caused by LINC01929 silencing (Fig. S3a–c). TFRC overexpression significantly reduced intracellular Fe²⁺ levels, whereas LINC01929 knockdown increased Fe²⁺ accumulation (Fig. S3d). Furthermore, qRT-PCR results showed that LINC01929 overexpression upregulated the anti-ferroptotic gene GPX4 and downregulated the pro-ferroptotic gene ACSL4, whereas TFRC knockdown had the opposite effect (Fig. S3e). Additional analyses using NC, OE-LINC01929, sh-TFRC, and OE-LINC01929 + sh-TFRC groups further showed that TFRC silencing markedly attenuated, but did not completely abolish, the protective effect of LINC01929 on Fe²⁺ accumulation and cell viability under erastin treatment (Fig. S3f, g). Together, these results suggest that the anti-ferroptotic/pro-survival phenotype associated with LINC01929 is at least partly dependent on TFRC. In addition, LINC01929 overexpression altered the expression of several ferroptosis-related proteins (decreased ACSL4, NCOA4, and FTL, but increased xCT and GPX4), while LINC01929 knockdown produced the opposite effects (Fig. 5e–j, *P* < 0.01). To further confirm that LINC01929 targets TFRC to influence BC metastasis in vivo, tumors overexpressing LINC01929 exhibited markedly higher TFRC expression, as shown by immunohistochemical staining, compared to the OE-NC group (Fig. S4, *P* < 0.01).

### TFRC promotes proliferation, migration and invasion of BC cells

**Fig. 6:**
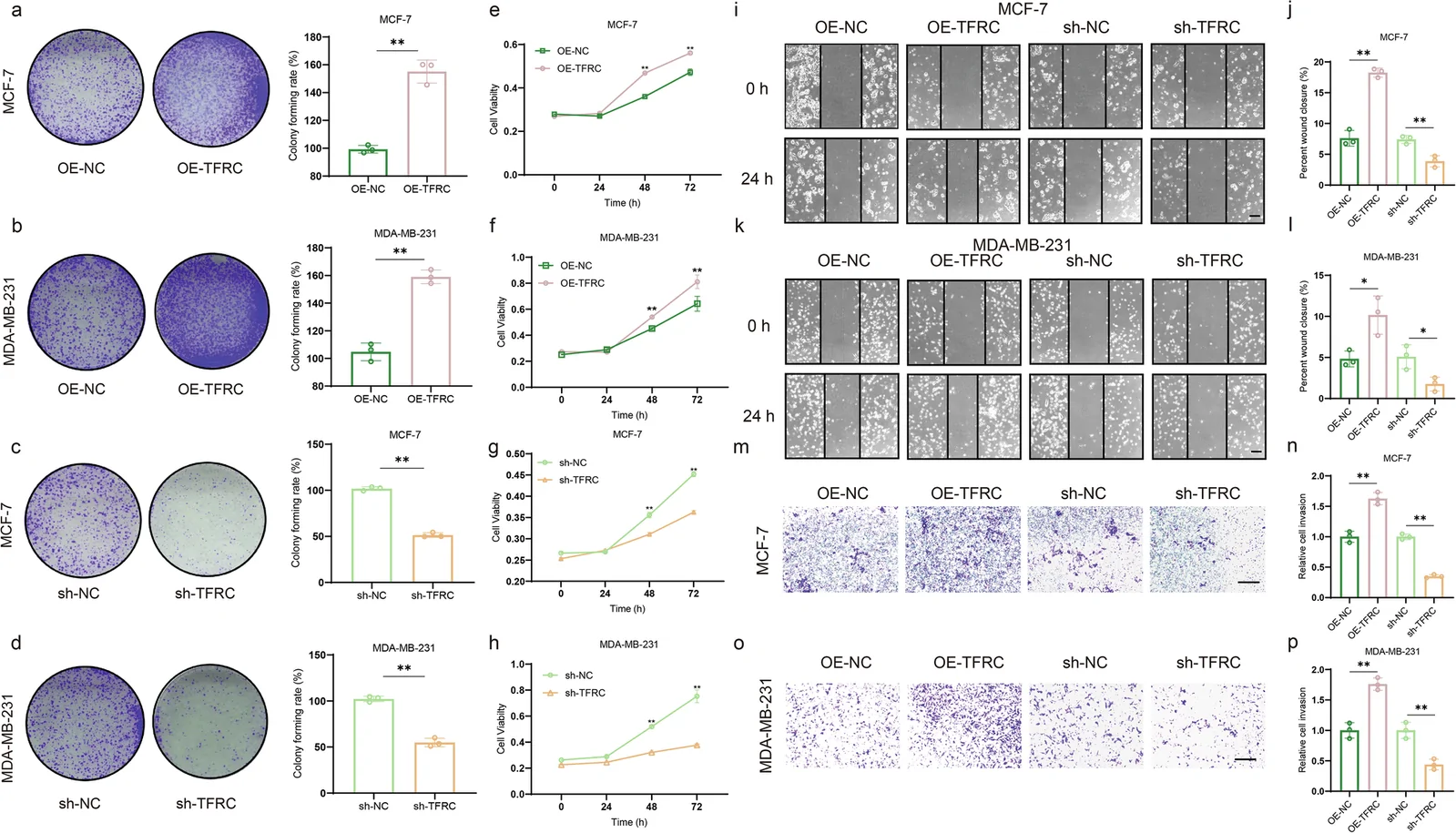
Effect of TFRC on proliferation, migration and invasion of breast cancer cells cultured in vitro*.* **a**–**d** Colony formation assays evaluating the proliferative capacity of BC cells (MCF-7 and MDA-MB-231) following TFRC overexpression, TFRC silencing, and empty vector transfection. Statistical evaluation of colony formation capability in MCF-7 and MDA-MB-231 cells (*n* = 4). **e**–**h** CCK-8 assays measuring cell proliferation in MCF-7 and MDA-MB-231 cells transfected with OE-TFRC or sh-TFRC at 24, 48, and 72 h (*n* = 5). **i**–**l** Wound healing assays assessing the migration capacity of MCF-7 and MDA-MB-231 cells transfected with TFRC excessive expression, TFRC knockdown, and empty vector at 24 h. Scale bar = 200 μm. A quantitative investigation of wound healing efficiency is provided (*n* = 3). **m–p** Transwell assays evaluating the invasive capacity of MCF-7 and MDA-MB-231 cells transfected with TFRC excessive expression, TFRC interference, and empty vector at 24 h. Scale bar = 100 μm. The statistical examination of cellular invasion capability is included (*n* = 3). The sh-NC and sh-TFRC groups serve as the control and knockdown groups, respectively, while the empty vector and OE-TFRC represent the control and overexpression groups. *P < 0.05; **P < 0.01.

To further confirm that the ferroptosis-related phenotype induced by LINC01929 knockdown was not due to off-target effects of a single shRNA construct, an additional shRNA (sh-LINC01929-2) was tested in parallel with sh-LINC01929-3. Consistent with the results obtained using sh-LINC01929-3, silencing LINC01929 with sh-LINC01929-2 significantly increased MDA levels in both MCF-7 and MDA-MB-231 cells, indicating enhanced lipid peroxidation (Fig. S5a). In contrast, control cells (sh-NC) exhibited markedly lower MDA levels. These findings demonstrate that the ferroptosis-promoting effects observed upon LINC01929 depletion are reproducible across two independent shRNA constructs, supporting that the phenotype is specific to LINC01929 knockdown rather than off-target activity. In addition, three different TFRC interfering shRNA plasmids and one overexpression plasmid were designed to transfect MCF-7 and MDA-MB-231 cell lines. Sh-TFRC-1 had the strongest inhibitory effect in both BC cell lines, according to the results of qRT-PCR (Fig. S5b). A TFRC overexpression vector was created using pcDNA3.1, and the expression level of this vector was measured (Fig. S5b, *P* < 0.05). Clone formation assays were conducted to assess changes in MCF-7 and MDA-MB-231 cell proliferation (Fig. 6a–d). BC cells with increased TFRC expression displayed a higher capacity for colony formation in contrast to the empty vector group. Conversely, inhibition of TFRC led to a marked reduction in colony number and size, especially in MCF-7 and MDA-MB-231 cells, relative to the empty vector controls (Fig. 6a–d, *P* < 0.01). The CCK-8 assay also showed that after OE-TFRC transfection, BC cell proliferation rates rose in comparison to the empty vector group, whereas TFRC interference dramatically decreased proliferation in MCF-7 and MDA-MB-231 cells over a 72-h period (Fig. 6e–h, *P* < 0.01). After TFRC overexpression, the wound-healing assay showed a marked improvement in BC migrate capacity. Conversely, within 24 h, cells transfected with sh-TFRC showed noticeably less capacity to migrate than the empty vector group (Fig. 6i–l, *P* < 0.05). Transwell assays demonstrated that TFRC overexpression promoted, while TFRC silencing suppressed, the invasive potential of MCF-7 and MDA-MB-231 cells compared to the empty vector controls (Fig. 6m–p, *P* < 0.01).

### LINC01929 interacts with TFRC to affect BC metastasis in vivo

**Fig. 7:**
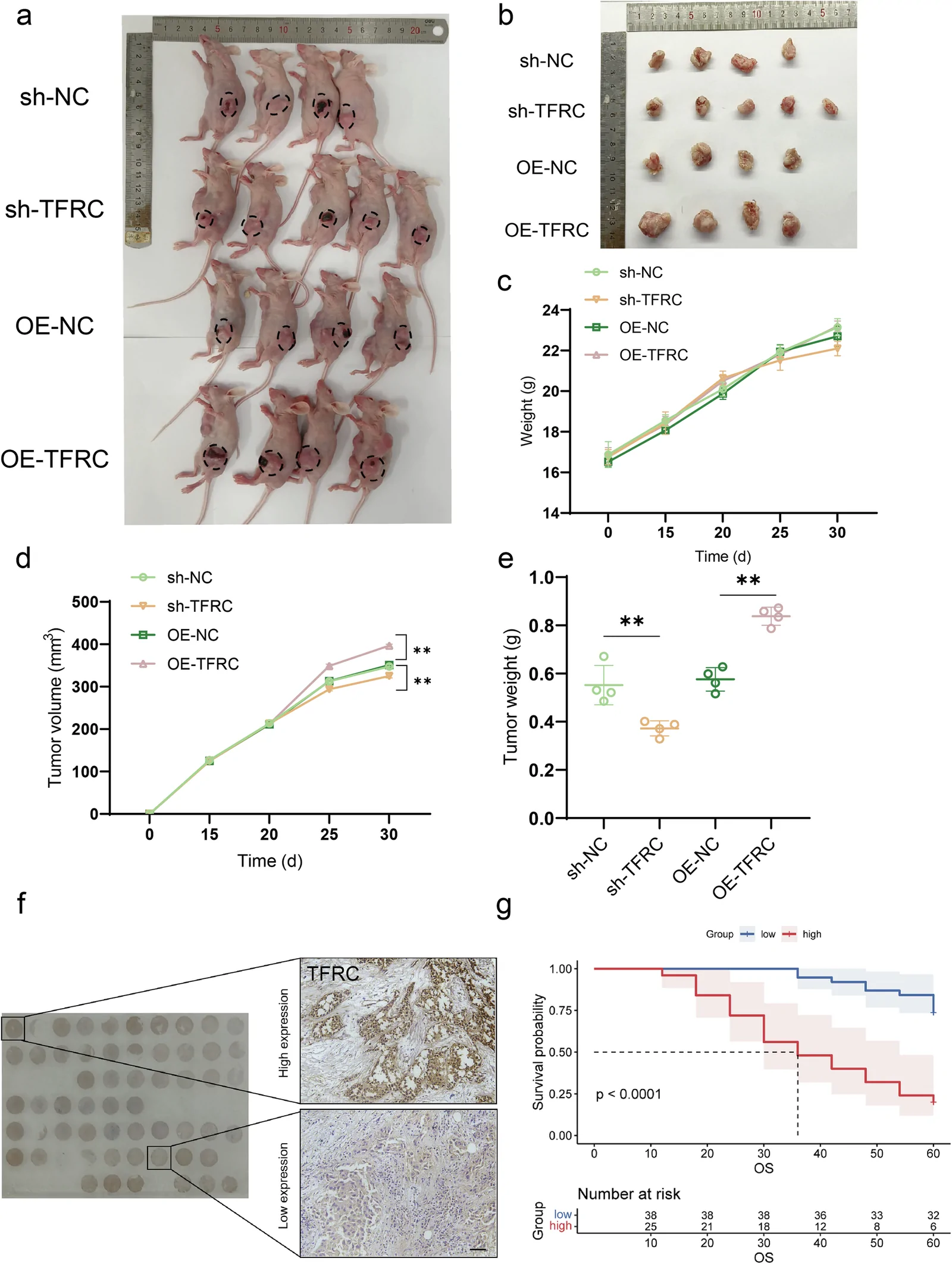
LINC01929 interacts with TFRC to promote BC metastasis in vivo. **a**, **b** After transfecting the TFRC overexpression vector, the control vector, the TFRC interference vector or the control vector into MDA-MB-231 cells, and injecting them into nude mice through the mammary fat pad to establish an in situ tumor model, the tumor photos were taken and observed 30 days later. **c** Body weight changes of nude mice over 30 days (*n* = 4–5). **d**, **e** Tumor growth curves and changes in tumor volume across different groups (*n* = 4–5). **f** Immunohistochemical analysis of TFRC expression in 57 BC tissue specimens, with representative images shown for two cases. Scale bar = 50 μm. **g** High TFRC expression correlates with unfavorable prognosis in clinical BC samples. The sh-NC and sh-TFRC groups serve as the control and knockdown groups, respectively, while the OE-NC and OE-TFRC represent the control and overexpression groups. *P < 0.05; **P < 0.01.

To investigate TFRC’s function in vivo, MDA-MB-231 cells stably expressing TFRC or a control vector were orthotopically injected into nude mice, successfully establishing orthotopic tumors (Fig. 7a). After 30 days, mice in both the empty vector control group and the TFRC overexpression group survived and developed tumors (Fig. 7b). The tumor weight, body weight, and tumor volume were tracked (Fig. 7c-e). The tumor development curve showed that the OE-TFRC group grew at a much faster pace than the OE-NC group, whereas the sh-TFRC group grew at a slower rate (Fig. 7d, *P* < 0.01). Additionally, in comparison to the control mice, the tumor weight and volume were considerably lower in the sh-TFRC group and much larger in the OE-TFRC group (Fig. 7e, *P* < 0.01).

### TFRC promotes BC progression by inhibiting ferroptosis

To test whether TFRC induces ferroptosis in BC cells, ferrous iron concentration was measured and found to be higher in sh-TFRC group, indicating that TFRC silencing promotes ferroptosis in BC cell (Fig. S6a, b, *P* < 0.05). To assess lipid peroxidation levels, we used flow cytometry after inducing ferroptosis with Erastin as a positive control, followed by TFRC silencing. Compared to the positive control group, the ROS levels in the sh-TFRC group were greater. These results suggest that TFRC promotes tumor progression by inhibiting ferroptosis in BC cells (Fig. S6c–e, *P* < 0.01). Western blot assays showed changes in the levels of other ferroptosis-related proteins after TFRC overexpression (ACSL4, NCOA4 and FT-L were reduced, and xCT and GPX4 were elevated), while TFRC knockdown produced the opposite effects. (Figure S6f, P < 0.01).

### TFRC expression is associated with poor prognosis in BC

To further investigate the association between TFRC expression and clinicopathological features in BC, we utilized a tissue microarray to assess TFRC expression levels in patient tumor samples with diverse pathological characteristics. TFRC expression was detected in 29 out of 57 BC samples (51%) (Fig. 7f). In addition, survival analysis was performed using data from 63 BC patients treated at the Affiliated Hospital of Nantong University, including 25 patients with high TFRC expression and 38 with low expression (Fig. 7g). Kaplan–Meier analysis revealed that patients with high TFRC expression exhibited significantly poorer overall survival compared to those with low TFRC expression (*P* < 0.001). TFRC expression was significantly associated with tumor grade (*χ*² = 7.725, *P* = 0.0054), tumor size (*χ*² = 7.458, *P* = 0.0063), estrogen receptor (ER) status (*χ*² = 16.61, *P* = 0.0063), progesterone receptor (PR) status (*χ*² = 5.054, *P* = 0.0246), and lymph node metastasis (*χ*² = 7.566, *P* = 0.0059). However, no significant correlation was observed between TFRC expression and patient age, HER2 status, Ki-67 index, or TNM stage (Table 1). Together, these findings suggest that elevated TFRC expression is associated with poor prognosis and more aggressive tumor features in BC. Furthermore, our data provide a mechanistic insight into how LINC01929 modulates ferroptosis in BC cells via TFRC regulation.

**Table 1:** Relations between TFRC and clinicopathological parameters of breast cancer.

Group	Number of cases (*n* = 63)	TFRC		*χ*^2^	*P* value
		Low (*n* = 38)	High (*n* = 25)		
Age (years)				0.2208	0.8955
≤40	8	5	3		
40–60	22	14	8		
≥60	33	19	14		
Grading of tumors				7.725	0.0054
Ⅰ–Ⅱ	51	35	16		
Ⅲ	12	3	9		
Tumor size (cm^3^)				7.458	0.0063
≤2	31	24	7		
>2	32	14	18		
ER (Estrogen receptor)				16.61	0.0001
Negative	30	26	4		
Positive	33	12	21		
PR (Progesterone receptor)				5.054	0.0246
Negative	43	30	13		
Positive	20	8	12		
HER2 (Human epidermal growth factor receptor 2)				0.8005	0.3709
Negative	49	31	18		
Positive	14	7	7		
Ki67				3.255	0.0712
Low	34	24	10		
High	29	14	15		
Lymphatic metastasis				7.566	0.0059
N0	36	27	9		
N1 + 2 + 3	27	11	16		
TNM				1.139	0.5659
Ⅰ	17	12	5		
Ⅱ	31	17	14		
Ⅲ	15	9	6		

### LINC01929 and TFRC manipulation promotes tumor growth in mice

To examine the role of LINC01929 in tumor growth, MCF-7 cells with LINC01929 overexpression (OE-LINC01929) and TFRC silencing (sh-TFRC) were implanted orthotopically in nude mice. Figure S7A shows representative images of orthotopic tumors at day 21, with OE-LINC01929 tumors being significantly larger than NC and OE-LINC01929 + sh-TFRC groups. Figure S7B presents the gross appearance of excised tumors. Figure S7C shows tumor weight at day 21, where OE-LINC01929 tumors were heavier than NC and sh-TFRC groups, and TFRC silencing reduced tumor weight. Figure S7D presents tumor volume over 21 days, with the OE-LINC01929 group showing significantly faster growth compared to the other groups (*P* < 0.05, *P* < 0.01, *P* < 0.001).

## Discussion

BC is the predominant cause of cancer mortality among women globally. Since drug resistance and metastasis are two of the main causes of high mortality, it is imperative to investigate novel therapeutic targets to combat these issues [23]. This study suggests that LINC01929 facilitates BC progression in association with reduced ferroptosis-related stress, with TFRC contributing to this phenotype in BC cells. Our findings show that LINC01929 enhances BC cell proliferation, migration, and invasion, and is associated with ferroptosis-related regulation in BC cells, underscoring its promise as a biomarker and target of therapy in BC. Recent research indicates that lncRNAs may contribute to solid tumors, including lung, liver, and stomach cancer [24–27]. In this study, the TCGA and GEO datasets were examined via bioinformatics methods to find differentially expressed lncRNAs in BC and adjacent non-cancerous tissues. LINC01929 is significantly expressed in BC and has been validated as substantially articulated in the highly metastatic human BC cell line MDA-MB-231. Moreover, elevated LINC01929 expression correlated with worse survival in BC patients, indicating its significance in clinical progression and outcomes. However, it is unclear whether LINC01929 is linked to the development of other human cancer types, thus, it will be crucial to investigate LINC01929 expression in pan-cancer studies and examine how it functions in the treatment of other malignancies. To examine the mechanism via which LINC01929 enhances BC growth and metastasis, we performed RNA pull down assay and KEGG pathway enrichment analysis. LINC01929 was closely associated with the ferroptosis pathway. These results provided an important basis for prioritizing downstream candidates involved in LINC01929-mediated BC proliferation, invasion, and migration. Through screening and validation, we identified TFRC as a key candidate associated with LINC01929 and ferroptosis-related regulation in BC cells.

Ferroptosis, a regulated type of cell death triggered by iron-dependent lipid peroxidation, is a prospective therapeutic target for cancer, especially in tumors that exhibit resistance to conventional therapies [28]. TFRC makes up the human transferrin receptor protein 1 (TFR1, UniProt-P02786), which regulates intracellular iron level [29, 30]. Ferric iron (Fe^3+^) attaches to transferrin in a soluble state and is carried into cells as part of the metabolism and transport of iron inside cells. Fe^3+^ is converted to ferrous iron (Fe^2+^) after passing through the inner membrane with the help of TFRC, which then enters the cytoplasmic labile iron pool [31, 32]. Excess Fe^2+^ in this pool induces ferroptosis through the production of ROS via the Fenton reaction. TFRC, a membrane protein, is widely expressed across nearly all cell and tissue types in the human body [33]. Its primary physiological function is to bind transferrin (TF) and mediate cellular iron uptake via endocytosis [34]. Transferrin facilitates iron absorption through receptor-mediated endocytosis and is implicated in cancer-associated ferroptosis [35, 36]. Yet, a number of publications indicate that TFRC is excessively expressed in certain human cancers, including astrocytic brain tumors and anaplastic thyroid carcinoma [37, 38]. However, in this latest study, we initially noted that TFRC expression was markedly increased in human BC tissues, and that overexpression of TFRC was directly correlating to a bad outcome. In this study, our data support ferroptosis as a major pathway associated with LINC01929 function in BC, with TFRC emerging as a key interacting candidate.

The function of ferroptosis, a form of non-apoptotic cell death, in tumor cells has been the subject of an increasing number of investigations [39]. The recent review by Chen [40] and Jiang [15] described that ferroptosis is intricately linked to cancer via several processes, including cellular hypoxia, epithelial-mesenchymal transition, immunological response, and numerous metabolic pathways. Additionally, several research have concentrated on the function of lncRNAs in cancer ferroptosis [41]. For example, Qi et al. [42] found that the long-chain non-coding RNA PGM5P3-AS1 stabilizes the manifestation of the target gene microtubule-associated protein light chain C by binding to the RNA binding protein NOP58, which promotes ferroptosis and then inhibits the malignant progression of triple-negative breast cancer. Zhang et al [43] discovered that LINC00460 prevented ferroptosis and increased BC cell proliferation via the miR-320a/MAL2 axis. Li et al. [44] discovered that ferroptosis in BC was reduced by POU2F2-mediated overexpression of lncRNA PTPRG-AS1 via the miR-376c-3p/SLC7A11 axis. Wang et al. [45] discovered that RUNX1-IT1 regulated the IGF2BP1/GPX4 axis, which in turn encouraged the occurrence and progression of BC. Accumulating evidence suggests that ferroptosis is a key tumor suppressor mechanism, and tumor cells develop various methods to evade ferroptosis. Our findings offer a fresh direction for ferroptosis in cancer cells.

The ability of LINC01929 to reduce ferroptosis-related stress in BC cells may reflect a survival adaptation exploited by tumor cells to evade oxidative damage. This mechanism is particularly relevant as iron metabolism and ferroptosis are increasingly recognized as pivotal regulators of tumor behavior. By influencing TFRC-associated ferroptosis-related phenotypes, LINC01929 may help BC cells sustain proliferation while reducing susceptibility to ferroptotic stress. In addition, in our study, LINC01929 was found to interact with other ferroptosis-related genes (DNAJB6, PCBP2, OTUB1, SLC3A2, PRDX6, GOT1) in addition to TFRC in the ferroptosis pathway, which may offer a novel avenue for investigating the mechanisms of ferroptosis. Our study also indicates that LINC01929 and TFRC expression levels may serve as predictive indicators of BC patient outcomes. High TFRC expression was associated with poor survival, reinforcing its role in supporting malignant behavior. These findings suggest that TFRC, together with LINC01929, may serve as a biomarker associated with BC aggressiveness and treatment responsiveness. Clinically, targeting the LINC01929-TFRC axis may provide a dual benefit: re-sensitizing BC cells to ferroptosis and simultaneously hindering their metastatic capabilities.

Ferroptosis-related lncRNAs may serve as predictors for the prognosis and outcome of BC. This elucidates further information understanding the function of lncRNAs and provides targets for therapy for oncological treatment. The application of ferroptosis inducers and inhibitors has significant promise in tumor therapy [27]. The activation of the ferroptosis pathway can promote the demise of cancer cells, particularly in instances of treatment resistance, hence augmenting the susceptibility to chemotherapeutic agents [46]. For example, the combination of cisplatin and the ferroptosis inducer Erastin markedly enhances anti-tumor efficacy, indicating the crucial importance of ferroptosis in cancer therapy [47]. Future directions for research should explore whether LINC01929 inhibition or TFRC modulation can effectively induce ferroptosis in other malignancies and assess the potential of LINC01929 inhibitors as therapeutic agents. Combining ferroptosis inducers with LINC01929 or TFRC-targeting agents may offer a potent strategy to overcome resistance mechanisms in BC, potentially enhancing the effectiveness of existing treatments. Additionally, given the intricate balance of ferroptosis with other forms of cell death like apoptosis, further studies are warranted to understand the interplay between these pathways, which may yield insights for combination therapies.

Although TFRC is generally recognized as a plasma membrane iron transporter associated with ferroptosis susceptibility, our data suggest that its role may be context dependent in BC cells. Similar anti-ferroptotic effects of TFRC regulation have been reported for circRAPGEF5, implying that TFRC may exert dual roles depending on the cellular environment [48, 49]. In our study, LINC01929 upregulation increased TFRC expression, reduced lipid peroxidation and ROS accumulation, and enhanced the expression of anti-ferroptotic markers (GPX4, xCT) while suppressing pro-ferroptotic markers (ACSL4, NCOA4). These findings indicate that LINC01929 may protect BC cells from ferroptosis-related stress in a TFRC-associated manner, thereby facilitating malignant progression.

In conclusion, our findings identify LINC01929 as an important promoter of BC progression that is associated with reduced ferroptosis-related stress, with TFRC contributing to this phenotype. By reducing ferroptosis-related oxidative stress, LINC01929 may enable BC cells to evade ferroptotic cell death and support malignant progression. The LINC01929-TFRC axis, therefore, represents a promising therapeutic target in BC, and interventions aimed at reactivating ferroptosis via this pathway may offer a novel approach to combat BC progression and improve patient outcomes. This study advances our understanding of lncRNA-mediated regulation in cancer and underscores the therapeutic potential of ferroptosis induction in controlling tumor growth and metastasis. Although our data suggest that LINC01929 can regulate ferroptosis-related phenotypes in tumor cells in association with TFRC expression, it is not clear whether the degree of limiting the occurrence of ferroptosis achieved in culture can be achieved in a physiological setting in vivo. In addition, this study focused on lncRNAs and cancer cells, and did not investigate the effect of the target in combination with the drug, or the effect of direct targeting by the vector, which would be more meaningful for the effect of clinical translation. Finally, our results were obtained in a limited number of cancer cell lines and only targeted the TFRC target. We did not explore the role of the remaining ferroptotic targets (DNAJB6, PCBP2, OTUB1, SLC3A2, PRDX6, GOT1) in this mechanism.

## Materials and methods

### Clinical samples

This study enrolled specimens from BC patients who underwent modified radical mastectomy at the Affiliated Hospital of Nantong University between January 2012 and December 2014.

Peripheral blood samples were collected in EDTA tubes, plasma was prepared by centrifugation, and LINC01929 expression was quantified by qRT-PCR using U6 as the internal control. The study protocol was reviewed and approved by the Ethics Committee of the Affiliated Hospital of Nantong University (Approval No.: 2024-K063-01), and conducted in accordance with the Declaration of Helsinki. Written informed consent was obtained from all participants prior to sample collection. All specimens were embedded in paraffin blocks and prepared as pathological tissue microarrays, with all cases histologically confirmed as invasive breast cancer. No individuals underwent preoperative radiotherapy, chemotherapy, or immunotherapy, and complete clinical data were recorded. All patients supplied consent with knowledge, and prognostic information was collected through telephone follow-up. The study protocol was approved by the Ethics Committee of the Affiliated Hospital of Nantong University. Following dewaxing, the tissue microarrays were stained using immunohistochemical techniques identical to those employed for subsequent staining of tumor tissues from nude mice. And diaminobenzidine (DAB) (DS-0005, Jinqiao, Zhongshan, China) and hematoxylin-eosin were performed to observe the expression of protein and cancer progression. Tissues from tumors and lungs were rapidly fixed in 4% paraformaldehyde for 24 h before embedding in paraffin. These sections were deparaffinized and hydrated before antigen repair with sodium citrate. Subsequent steps included incubation with 0.3% hydrogen peroxide and primary antibody against transferrin receptor (TFRC) (1:100, 7037-MSM4-P1, Invitrogen, USA). Imaging was conducted with a DM4000B microscope (Leica, Germany).

### Cell culture

MCF-10A cells were purchased from Shanghai Zhong Qiao Xin Zhou Biotechnology Co., Ltd. (Cat. No. ZQ0451, Shanghai, China). BC cell lines, including MCF-7, MDA-MB-468, MDA-MB-231, and BT-474, were acquired from the National Certified Cell Culture Collection Center (Shanghai, China). These cell lines were sustained at 37 °C with 5% CO_2_ in Dulbecco’s Modified Eagle Medium (DMEM), which contained 10% FBS, 100 μg/mL penicillin, and 100 μg/mL streptomycin. All cell lines used in this study were confirmed to be free of mycoplasma contamination and authenticated by short tandem repeat (STR) profiling prior to use.

### Bioinformatic analysis

The transcription of omics data from the GEO database of the GSE119233 data set (http://www.ncbi.nlm.nih.gov) and the TCGA database (https://portal.gdc.cancer.gov) is sampled by BC. LncRNA annotations adhere to the GRCh38 standard of the GENCODE database (https://www.gencodegenes.org/). Transcriptome data comprising 112 normal samples and 1096 BC samples were obtained from the TCGA database. The research also contained GSE119233, which comprises 20 BC samples and 10 normal tissues. The limma R tool in R version 4.4 was used to log2 transform and normalize the data (R Foundation for Statistical Computing, Austria) [21]. Subsequently, common differentially expressed genes from both datasets were retrieved utilizing the Venn R program, and differential gene analysis was conducted with the limma R package to compare BC and normal samples. Heat maps and volcano plots were used to visualize the data in a comprehensive way, and Kaplan-Meier analysis was used to evaluate survival trends. In addition, Gene Ontology (GO) enrichment analysis was performed using the Metascape portal (https://metascape.org) to identify biological functions associated with mRNAs related to LncRNAs. Using R software package for enrichment of GO analysis and the enrichment of the Kyoto encyclopedia (KEGG) gene and genome analysis [50, 51]. The threshold requirement for GO and KEGG pathway enrichment analysis was established at *p*-value < 0.01. The results are shown as bubble plots by “ggplot2”. The R software was utilized to assess the survival prognosis of differentially expressed genes, and the survival curve was generated. The CatRAPID database (http://service.tartaglialab.com/page/catrapid_group) was used to predict proteins that interact with the indicated lncRNAs.

### Plasmid construction and transfection

LINC01929 control vector (sh-NC, OE-NC), overexpression plasmid (OE-LINC01929), interference plasmid (sh-LINC01929), overexpression plasmid (OE-TFRC), and TFRC interference plasmid (sh-TFRC) (Table S1**details the sequences and types of plasmid constructs used**) were acquired from OBiO Technology in Shanghai, China. Plasmid transfection was performed in accordance with the manufacturer’s guidelines utilizing Lipofectamine 2000 reagent (11668027, Invitrogen, USA), and shRNA was used to temporarily mute the target genes. After 48 h of plasmid and shRNA transfection, BC cells were prepared for additional analysis.

### Quantitative real-time polymerase chain reaction (qRT-PCR)

Total RNA was isolated from cultivated cells utilizing TRIzol (15596018, Invitrogen, USA) in compliance with the manufacturer’s guidelines. The amount and purity of total RNA were checked using a Biophotometer plus (Eppendorf, Germany), and RNA underwent reverse transcription to cDNA with HiScript II RT SuperMix. qRT-PCR analysis was then performed using Universal SYBR qRT-PCR Master Mix (Vazyme, China). Comparative gene expression standard was determined utilizing the 2^−ΔΔCt^. GAPDH expression was assessed simultaneously on the same plate as normalized mRNA. The sets of primers employed in this work are enumerated in Table S2.

### Western blot analysis

Proteins were recovered from the cells utilizing Radio Immunoprecipitation Assay (RIPA) lysis buffer (P0013B, Beyotime Biotechnology) in accordance with the manufacturer’s guidelines. A comparable quantity of protein from every specimen was analyzed using 10% Sodium Dodecyl Sulfate-Polyacrylamide Gel Electrophoresis (SDS-PAGE) and subsequently transferred to Polyvinylidene Fluoride (PVDF) membranes. After blocking with 5% milk, the blots were mixed with TFRC (7037-MSM4-P1, Invitrogen, USA), Acyl-CoA Synthetase Long Chain Family Member 4 (ASCL4) (sc-271800, Santacruz, USA), Nuclear Receptor Coactivator 4 (NCOA4) (EPR28511-12, Abcam, UK), Ferroportin 1 (FPN1) (NBP1-21502, Novus, USA), Cystine/Glutamate Transporter (xCT) (EPR27115-64, Abcam, USA), Ferritin Light Chain (FT-L) (EPR5260, Abcam, UK), Glutathione Peroxidase 4 (GPX4) (EPNCIR144, Abcam, UK) and β-actin (Sigma, USA) were cultivated together with chief antibodies overnight at 4 °C, after which the membranes are incubated with the proper secondary antibodies and detection of protein bands using an enhanced chemiluminescence (ECL) detection system (Tanon, China). By chemiluminescence, the color contrast of the bands was detected to reflect the protein expression. The total protein normalization was performed using β-actin. Protein bands were quantified using ImageJ, and the intensity of the target protein was normalized to the loading control to account for differences in protein loading.

### Colony formation assay and cell counting kit-8 (CCK-8)

Forty-eight hours post-transfection, cells from the different groups were plated on 6-well plates at a specific mass of 5 × 10^2^ to 1 × 10^3^ cells per well for 14 days at 37 °C in 5% CO_2_ to aid colony formation. Upon conclusion of the experiment, cell colonies were preserved using 4% paraformaldehyde and subsequently stained with 0.5% crystal violet (Sigma-Aldrich). Then took pictures and counted the colonies. As previously mentioned, 96-well plates (Corning, USA) were filled with 1000–2000 cells/100 µL of transfected BC cells. After adding 10 µL CCK-8 reagent (Beyotime Biotechnology, China) for 0, 24, 48, 72 h and incubation for 1 h, the continuous determination was performed. A microplate reader (Bio-Tek, USA) was employed to assess absorbance at 450 nm. A higher absorbance value indicates a higher proliferative capacity. Wells without cells were used as negative controls. For ferroptosis-related viability assays, transfected cells were treated with erastin for 24 h, and relative cell viability was assessed by CCK-8.

### Cell migration and invasion assays

The ability of cells to migrate was evaluated by a wound-healing experiment in accordance with standard laboratory techniques. Forty-eight hours post-transfection, cells proliferated and aggregated to establish monolayers at the base of 6-well plates (Corning, USA). A wound was inflicted by abrading the monolayer with a sterile 200 µL pipette tip (KIRGRN, China). Cells were meticulously rinsed with PBS and subsequently substituted with serum-free media. Upon an incubation period of 24 hours at 37 °C in a 5% CO_2_ environment, the wound area was remeasured. Transwell assay was employed to assess cellular invasion capability. In addition, 5 × 10^4^ MDA-MB-231 cells and 1 × 10^5^ MCF-7 cells were inoculated in 8 µm transwell migration chambers (Corning, 3422, USA) in medium containing 1% FBS. The cells were inoculated for 24 h in a chamber with 600 µL of medium containing 10% FBS. The invaded cells were fixed, stained and subsequently examined by microscopy (Leica, Germany).

### Terminal deoxynucleotidyl transferase-mediated dUTP nick end labeling (TUNEL) assay

After being grown in 6-well plates, MDA-MB-231 cells were transfected with plasmids for 48 h, rinsed with PBS, and fixed with 4% paraformaldehyde. Adhering to the manufacturer’s guidelines, TUNEL assays were carried out using a one-step TUNEL apoptosis Detection kit (green fluorescence) (Beyotime, C1086). Lastly, 4’,6-Diamidino-2-Phenylindole (DAPI) was used to stain the nuclei, and a laser confocal microscope TCS SP8 (Leica, GER) was used to record the fluorescence signals.

### Ferrous iron (Fe^2+^) level assay

Iron determination kit (TC1015; LEAGENE, Guangzhou, China) for Fe^2+^ level measurement. Transfected cells were lysed on ice and centrifuged at 12500 rpm. After that, the supernatant was gathered and mixed with the ferrireductase-containing reagent. Following a 30-min incubation, the absorbance at 562 nm was measured using a microplate reader (Bio-Tek, USA).

### Measurement of MDA and GSH/GSSG ratio

The levels of malondialdehyde (MDA) and the ratio of reduced to oxidized glutathione (GSH/GSSG) were determined to evaluate lipid peroxidation and redox balance in breast cancer cells. Briefly, MCF-7 and MDA-MB-231 cells from each treatment group were collected and lysed in ice-cold buffer. The MDA concentration was measured using a lipid peroxidation (MDA) assay kit (BOXBIO, China) based on the reaction of MDA with thiobarbituric acid (TBA) to form a colored adduct, which was quantified at 532 nm using a microplate reader. And the glutathione (GSH) and oxidized glutathione (GSSG) assay kit (Beyotime, China) was used to detect GSH level and GSH/GSSG ratio in cells following the manufacturer’s protocol.

### Reactive oxygen species (ROS) assay

The cells treated with Erastin for 24 h served as the positive control group, while the cells left untreated served as the blank control group. Following a 30-min incubation at 37 °C with DCFH-DA (MCE, USA), the cells went through washing three times with PBS. Cells were harvested using trypsin digestion, resuspended in 300 μL of PBS with 10% FBS, and subsequently filtered through a 40 μm cell filter (BD Falcon). Using flow cytometry (BECKMAN COULTER, USA) with a 488 nm laser on a FL1 detector to measure ROS, the fluorescence of cells was assessed. For every condition, at least 10,000 single cells were examined. FlowJo software (version 10.0) was utilized for assessing the intensity of ROS fluorescence.

### RNA pull-down mass spectrometry (MS) assay

As directed by the manufacturer, the RNA Structure Buffer (Fab Bio, item number: FP1823) was used. In summary, the LINC01929 and LINC01929 antisense sequences (Table S3) were transcribed in vitro using the mMESSAGE mMACHINE™ T7 Transcription Kit (AM1344, Invitrogen, USA), followed by purification. Following RNA pull-down, the resulting RNA was biotinylated, and the biotin-labeled RNA was treated for 30 min with rotating RNA catch buffer and streptavidin magnetic beads. Following a magnetic separator separation of the precipitate, 100 µL of protein-RNA binding buffer was added, and the mixture was rotated for 60 min during incubation. The obtained proteins were subsequently extracted, combined with the biotinylated RNA solution and SDS loading buffer, heated to 95 °C for 5 min, and then detected by western blot. The Pull-down solution was subsequently analyzed by liquid chromatography.

### RNA immunoprecipitation assay

RIP assays were performed using the Sigma‑Aldrich Imprint RNA Immunoprecipitation Kit (Catalog No. RIP‑12RXN) according to the manufacturer’s instructions. Briefly, cell lysates were incubated with anti‑TFRC antibody or IgG control, followed by capture with Protein A/G magnetic beads supplied in the kit. Co‑precipitated RNA was purified and analyzed by RT‑qPCR.

### Protein-protein interaction (PPI) network analysis

Protein interactions that are likely to occur were gathered and compiled utilizing the STRING database (https://string-db.org). PPI network analysis was performed using the associated genes that were acquired. The criterion for significance was determined to be a confidence score greater than 0.7. Then the pertinent data were imported into Cytoscape (v3.8.2) for display and future analysis. The Cytoscape cytoHubba plug-in was used to identify key modules, and the Maximum Clique Centrality (MCC) ranking of cytoHubba was used to identify the top 10 nodes as hub genes.

### Tumor xenograft assays

Four-week-old female BALB/c mice, with a weight range of 22–25 g, were acquired from the Laboratory Animal Center of Nantong University (Institutional License: SYXK(SU)-2012-0030). All murine research received clearance from the Animal Ethics Committee of Nantong University (approval number: S20221115-902). And complied with the National Institutes of Health guidelines for the care and utilization of laboratory animals. Inclusion criteria for this study required that mice be healthy, with no signs of infection or injury, and fall within the specified weight range. Exclusion criteria were pre-established and included animals that showed signs of severe illness, infection, or injury during the study period. Mice that did not show tumor formation within a designated timeframe or exhibited abnormal health decline, such as significant body weight loss (greater than 20% of initial body weight), were also excluded from the analysis. Mice were monitored for overall health status, including body weight and tumor volume, throughout the duration of the study. Tumors were measured regularly using calipers, and any animal exhibiting significant health concerns, such as abnormal tumor growth or failure to respond to the experimental treatment, was removed from the study. The MDA-MB-231 cells that had been transfected with OE-LINC01929 and sh-LINC01929, as well as the corresponding control cells, were transplanted subcutaneously at the waist of mice. The MDA-MB-231 cells that had been transfected with OE-TFRC and sh-TFRC, as well as the corresponding control cells, were transplanted into the mammary fat pads of mice. A caliper was used to measure the tumors on a regular basis, and the formula π/6 × *L* × *W*^2^ (where *L* is the longest diameter and *W* is the shortest) was used to determine the volume [52]. Changes in the body weight and tumor volume of naked mice were tracked and documented throughout the tumor formation process. Prior to lung and tumor removal, mice were given an intraperitoneal injection of sodium pentobarbital (Cat# P-010, Millipore Sigma, USA) to induce anesthesia when the tumors reached a size of 1000–1500 mm^3^, and the tumor’s weight was recorded. Animals were randomly assigned to the experimental groups prior to tumor cell inoculation to minimize selection bias. Randomization was performed using a computer-generated random number list, ensuring that each mouse had an equal probability of being allocated to any group. Group allocation was carried out by a researcher who was not involved in data collection or analysis, in order to maintain allocation concealment and reduce experimental bias. To reduce experimental bias, the investigator responsible for measuring tumor size and assessing experimental outcomes was blinded to the group allocation. Group identities were coded and not revealed until all measurements and data recording were completed. However, blinding was not applied during the tumor cell inoculation stage, as the procedure required knowledge of the corresponding cell line to be implanted. This approach ensured objective outcome assessment while maintaining practical feasibility during the experimental procedures.

### Sample sizes chosen

The sample sizes chosen for both the cell-based and animal experiments were justified based on standard practices in preclinical research and published recommendations. For the in vitro experiments, each treatment group consisted of *n* = 3 independent biological replicates, which is consistent with commonly accepted practices for assays where within-group variability is relatively low, ensuring adequate power to detect meaningful differences. For the animal studies, each experimental group included 4–5 animals. Although a formal a priori power analysis was not conducted, this sample size was selected following established guidance that aims to balance the ability to detect a biologically meaningful effect with ethical considerations regarding animal use. Previous studies, such as Dell et al. [53], suggest that sample sizes should be chosen to provide sufficient power while minimizing animal use. Additionally, contemporary reviews emphasize the importance of balancing statistical sensitivity with ethical and resource constraints to avoid under-powered studies that may yield ambiguous results, or excessively large groups that waste resources. Based on pilot studies and prior experience in similar models, we determined that a sample size of 4–5 animals per group was adequate to detect the expected effect size while minimizing animal use.

### Statistical analysis

GraphPad Prism version 8.0 and the R language of programming (version 4.4) served as platforms for data analysis. Prior to applying parametric statistical tests, the normality of data distribution was assessed using the Shapiro–Wilk test, and homogeneity of variance was evaluated using Levene’s test. For data that satisfied assumptions of normal distribution and equal variance, two-tailed unpaired Student’s *t*-tests (two groups) or one-way ANOVA followed by Tukey’s post hoc test (multiple groups) were applied. For data that did not meet these assumptions, non-parametric tests such as the Mann–Whitney *U* test or Kruskal–Wallis test were used as appropriate. Data are presented as mean ± standard deviation (SD) unless otherwise stated. The number of biological replicates (*n*) is indicated in each figure legend. Estimates of variation within each group are shown through SD error bars. Group variances were confirmed to be similar across statistically compared groups, and no statistical outliers were removed unless justified and described. The survival rate was determined using the Kaplan–Meier method. The threshold for statistical significance was set at a *P*-value of less than 0.05. At least three duplicates of each experiment were conducted.

## Supplementary information

Supplemental materials

Full uncropped Gels and Blots images

## Data Availability

Data generated or analyzed during this study are included in this article. Further enquiries can be directed to the corresponding author.

## References

[1] Bray F, Laversanne M, Sung H, Ferlay J, Siegel RL, Soerjomataram I, et al. Global cancer statistics 2022: GLOBOCAN estimates of incidence and mortality worldwide for 36 cancers in 185 countries. CA Cancer J Clin. 2024;74:229–63. 10.3322/caac.21834 38572751

[2] Miller KD, Nogueira L, Devasia T, Mariotto AB, Yabroff KR, Jemal A, et al. Cancer treatment and survivorship statistics, 2022. CA Cancer J Clin. 2022;72:409–36. 10.3322/caac.21731 35736631

[3] Statello L, Guo CJ, Chen LL, Huarte M. Gene regulation by long non-coding RNAs and its biological functions. Nat Rev Mol Cell Biol. 2021;22:96–118. 10.1038/s41580-020-00315-9 PMC7754182 33353982

[4] Fumagalli C, Ranghiero A, Gandini S, Corso F, Taormina S, De Camilli E, et al. Inter-tumor genomic heterogeneity of breast cancers: comprehensive genomic profile of primary early breast cancers and relapses. Breast Cancer Res. 2020;22:107. 10.1186/s13058-020-01345-z PMC7566144 33059724

[5] Rosati D, Giordano A. Single-cell RNA sequencing and bioinformatics as tools to decipher cancer heterogenicity and mechanisms of drug resistance. Biochem Pharmacol. 2022;195:114811. 10.1016/j.bcp.2021.114811 34673017

[6] Yuan K, Lan J, Xu L, Feng X, Liao H, Xie K, et al. Long noncoding RNA TLNC1 promotes the growth and metastasis of liver cancer via inhibition of p53 signaling. Mol Cancer. 2022;21:105. 10.1186/s12943-022-01578-w PMC9044722 35477447

[7] Park MK, Zhang L, Min KW, Cho JH, Yeh CC, Moon H, et al. NEAT1 is essential for metabolic changes that promote breast cancer growth and metastasis. Cell Metab. 2021;33:2380–97.e9. 10.1016/j.cmet.2021.11.011 PMC8813003 34879239

[8] Luo W, Wang J, Xu W, Ma C, Wan F, Huang Y, et al. LncRNA RP11-89 facilitates tumorigenesis and ferroptosis resistance through PROM2-activated iron export by sponging miR-129-5p in bladder cancer. Cell Death Dis. 2021;12:1043. 10.1038/s41419-021-04296-1 PMC8563982 34728613

[9] Esposito R, Bosch N, Lanzós A, Polidori T, Pulido-Quetglas C, Johnson R. Hacking the cancer genome: profiling therapeutically actionable long non-coding RNAs using CRISPR-Cas9 screening. Cancer Cell. 2019;35:545–57. 10.1016/j.ccell.2019.01.019 30827888

[10] Kim J, Piao HL, Kim BJ, Yao F, Han Z, Wang Y, et al. Long noncoding RNA MALAT1 suppresses breast cancer metastasis. Nat Genet. 2018;50:1705–15. 10.1038/s41588-018-0252-3 PMC6265076 30349115

[11] Dhanoa JK, Sethi RS, Verma R, Arora JS, Mukhopadhyay CS. Long non-coding RNA: its evolutionary relics and biological implications in mammals: a review. J Anim Sci Technol. 2018;60:25. 10.1186/s40781-018-0183-7 PMC6201556 30386629

[12] Goodall GJ, Wickramasinghe VO. RNA in cancer. Nat Rev Cancer. 2021;21:22–36. 10.1038/s41568-020-00306-0 33082563

[13] Xiong Y, Pang M, Du Y, Yu X, Yuan J, Liu W, et al. The LINC01929/miR-6875-5p/ADAMTS12 axis in the ceRNA network regulates the development of advanced bladder cancer. Front Oncol. 2022;12:856560. 10.3389/fonc.2022.856560 PMC9133480 35646642

[14] Che H, Che Y, Zhang Z, Lu Q. Long non-coding RNA LINC01929 accelerates progression of oral squamous cell carcinoma by targeting the miR-137-3p/FOXC1 axis. Front Oncol. 2021;11:657876. 10.3389/fonc.2021.657876 PMC8097103 33968763

[15] Jiang X, Stockwell BR, Conrad M. Ferroptosis: mechanisms, biology and role in disease. Nat Rev Mol Cell Biol. 2021;22:266–82. 10.1038/s41580-020-00324-8 PMC8142022 33495651

[16] Su Y, Zhao B, Zhou L, Zhang Z, Shen Y, Lv H, et al. Ferroptosis, a novel pharmacological mechanism of anti-cancer drugs. Cancer Lett. 2020;483:127–36. 10.1016/j.canlet.2020.02.015 32067993

[17] Lei G, Zhuang L, Gan B. Targeting ferroptosis as a vulnerability in cancer. Nat Rev Cancer. 2022;22:381–96. 10.1038/s41568-022-00459-0 PMC10243716 35338310

[18] Yao X, Li W, Fang D, Xiao C, Wu X, Li M, et al. Emerging roles of energy metabolism in ferroptosis regulation of tumor cells. Adv Sci. 2021;8:e2100997. 10.1002/advs.202100997 PMC8596140 34632727

[19] Zhao L, Zhou X, Xie F, Zhang L, Yan H, Huang J, et al. Ferroptosis in cancer and cancer immunotherapy. Cancer Commun. 2022;42:88–116. 10.1002/cac2.12250 PMC8822596 35133083

[20] Xu H, Ye D, Ren M, Zhang H, Bi F. Ferroptosis in the tumor microenvironment: perspectives for immunotherapy. Trends Mol Med. 2021;27:856–67. 10.1016/j.molmed.2021.06.014 34312075

[21] Ritchie ME, Phipson B, Wu D, Hu Y, Law CW, Shi W, et al. limma powers differential expression analyses for RNA-sequencing and microarray studies. Nucleic Acids Res. 2015;43:e47. 10.1093/nar/gkv007 PMC4402510 25605792

[22] Gao W, Wang X, Zhou Y, Wang X, Yu Y. Autophagy, ferroptosis, pyroptosis, and necroptosis in tumor immunotherapy. Signal Transduct Target Ther. 2022;7:196. 10.1038/s41392-022-01046-3 PMC9208265 35725836

[23] Yao RW, Wang Y, Chen LL. Cellular functions of long noncoding RNAs. Nat Cell Biol. 2019;21:542–51. 10.1038/s41556-019-0311-8 31048766

[24] McCabe EM, Rasmussen TP. lncRNA involvement in cancer stem cell function and epithelial-mesenchymal transitions. Semin Cancer Biol. 2021;75:38–48. 10.1016/j.semcancer.2020.12.012 33346133

[25] Wang M, Mao C, Ouyang L, Liu Y, Lai W, Liu N, et al. Long noncoding RNA LINC00336 inhibits ferroptosis in lung cancer by functioning as a competing endogenous RNA. Cell Death Differ. 2019;26:2329–43. 10.1038/s41418-019-0304-y PMC6889193 30787392

[26] Qu X, Liu B, Wang L, Liu L, Zhao W, Liu C, et al. Loss of cancer-associated fibroblast-derived exosomal DACT3-AS1 promotes malignant transformation and ferroptosis-mediated oxaliplatin resistance in gastric cancer. Drug Resist Updat. 2023;68:100936. 10.1016/j.drup.2023.100936 36764075

[27] Qi W, Li Z, Xia L, Dai J, Zhang Q, Wu C, et al. LncRNA GABPB1-AS1 and GABPB1 regulate oxidative stress during erastin-induced ferroptosis in HepG2 hepatocellular carcinoma cells. Sci Rep. 2019;9:16185. 10.1038/s41598-019-52837-8 PMC6838315 31700067

[28] Yang WS, SriRamaratnam R, Welsch ME, Shimada K, Skouta R, Viswanathan VS, et al. Regulation of ferroptotic cancer cell death by GPX4. Cell. 2014;156:317–31. 10.1016/j.cell.2013.12.010 PMC4076414 24439385

[29] Moharir SC, Sirohi K, Swarup G. Regulation of transferrin receptor trafficking by optineurin and its disease-associated mutants. Prog Mol Biol Transl Sci. 2023;194:67–78. 10.1016/bs.pmbts.2022.06.019 36631201

[30] Dinh HQ, Eggert T, Meyer MA, Zhu YP, Olingy CE, Llewellyn R, et al. Coexpression of CD71 and CD117 identifies an early unipotent neutrophil progenitor population in human bone marrow. Immunity. 2020;53:319–34.e6. 10.1016/j.immuni.2020.07.017 PMC7942809 32814027

[31] Lakhal-Littleton S, Wolna M, Carr CA, Miller JJ, Christian HC, Ball V, et al. Cardiac ferroportin regulates cellular iron homeostasis and is important for cardiac function. Proc Natl Acad Sci USA. 2015;112:3164–9. 10.1073/pnas.1422373112 PMC4364209 25713362

[32] Gao M, Monian P, Quadri N, Ramasamy R, Jiang X. Glutaminolysis and transferrin regulate ferroptosis. Mol Cell. 2015;59:298–308. 10.1016/j.molcel.2015.06.011 PMC4506736 26166707

[33] Qin J, Li Z, Su L, Wen X, Tang X, Huang M, et al. Expression of transferrin receptor/TFRC protein in bladder cancer cell T24 and its role in inducing iron death in bladder cancer. Int J Biol Macromol. 2024;274:133323. 10.1016/j.ijbiomac.2024.133323 38908617

[34] Shi H, Bencze KZ, Stemmler TL, Philpott CC. A cytosolic iron chaperone that delivers iron to ferritin. Science. 2008;320:1207–10. 10.1126/science.1157643 PMC2505357 18511687

[35] Kawabata H. Transferrin and transferrin receptors update. Free Radic Biol Med. 2019;133:46–54. 10.1016/j.freeradbiomed.2018.06.037 29969719

[36] Yang C, Li J, Guo Y, Gan D, Zhang C, Wang R, et al. Role of TFRC as a novel prognostic biomarker and in immunotherapy for pancreatic carcinoma. Front Mol Biosci. 2022;9:756895. 10.3389/fmolb.2022.756895 PMC8965864 35372510

[37] Parenti R, Salvatorelli L, Magro G. Anaplastic thyroid carcinoma: current treatments and potential new therapeutic options with emphasis on TfR1/CD71. Int J Endocrinol. 2014;2014:685396. 10.1155/2014/685396 PMC4102021 25097549

[38] Rosager AM, Sørensen MD, Dahlrot RH, Hansen S, Schonberg DL, Rich JN, et al. Transferrin receptor-1 and ferritin heavy and light chains in astrocytic brain tumors: expression and prognostic value. PLoS ONE. 2017;12:e0182954. 10.1371/journal.pone.0182954 PMC5570299 28837569

[39] Wang H, Cheng Y, Mao C, Liu S, Xiao D, Huang J, et al. Emerging mechanisms and targeted therapy of ferroptosis in cancer. Mol Ther. 2021;29:2185–208. 10.1016/j.ymthe.2021.03.022 PMC8261167 33794363

[40] Chen X, Kang R, Kroemer G, Tang D. Broadening horizons: the role of ferroptosis in cancer. Nat Rev Clin Oncol. 2021;18:280–96. 10.1038/s41571-020-00462-0 33514910

[41] Zhi Y, Gao L, Wang B, Ren W, Liang KX, Zhi K. Ferroptosis holds novel promise in treatment of cancer mediated by non-coding RNAs. Front Cell Dev Biol. 2021;9:686906. 10.3389/fcell.2021.686906 PMC8255676 34235152

[42] Qi L, Sun B, Yang B, Lu S. PGM5P3-AS1 regulates MAP1LC3C to promote cell ferroptosis and thus inhibiting the malignant progression of triple-negative breast cancer. Breast Cancer Res Treat. 2022;193:305–18. 10.1007/s10549-021-06501-3 35325342

[43] Zhang C, Xu L, Li X, Chen Y, Shi T, Wang Q. LINC00460 facilitates cell proliferation and inhibits ferroptosis in breast cancer through the miR-320a/MAL2 axis. Technol Cancer Res Treat. 2023;22:15330338231164359. 10.1177/15330338231164359 PMC10028644 36938678

[44] Li J, Li PT, Wu W, Ding BN, Wen YG, Cai HL, et al. POU2F2-mediated upregulation of lncRNA PTPRG-AS1 inhibits ferroptosis in breast cancer via miR-376c-3p/SLC7A11 axis. Epigenomics. 2024;16:215–31. 10.2217/epi-2023-0100 38318853

[45] Wang S, Wang Y, Li Q, Zeng K, Li X, Feng X. RUNX1-IT1 favors breast cancer carcinogenesis through regulation of IGF2BP1/GPX4 axis. Discov Oncol. 2023;14:42. 10.1007/s12672-023-00652-z PMC10086083 37036576

[46] Yu H, Guo P, Xie X, Wang Y, Chen G. Ferroptosis, a new form of cell death, and its relationships with tumourous diseases. J Cell Mol Med. 2017;21:648–57. 10.1111/jcmm.13008 PMC5345622 27860262

[47] Sato M, Kusumi R, Hamashima S, Kobayashi S, Sasaki S, Komiyama Y, et al. The ferroptosis inducer erastin irreversibly inhibits system x(c)- and synergizes with cisplatin to increase cisplatin’s cytotoxicity in cancer cells. Sci Rep. 2018;8:968. 10.1038/s41598-018-19213-4 PMC5772355 29343855

[48] Zhang J, Chen S, Wei S, Cheng S, Shi R, Zhao R, et al. CircRAPGEF5 interacts with RBFOX2 to confer ferroptosis resistance by modulating alternative splicing of TFRC in endometrial cancer. Redox Biol. 2022;57:102493. 10.1016/j.redox.2022.102493 PMC9526237 36182807

[49] Wang J, Xiu M, Wang J, Gao Y, Li Y. METTL16-SENP3-LTF axis confers ferroptosis resistance and facilitates tumorigenesis in hepatocellular carcinoma. J Hematol Oncol. 2024;17:78. 10.1186/s13045-024-01599-6 PMC11367782 39218945

[50] Gene Ontology Consortium. Gene Ontology Consortium: going forward. Nucleic Acids Res. 2015;43:D1049–56. 10.1093/nar/gku1179 PMC4383973 25428369

[51] Kanehisa M, Goto S. KEGG: kyoto encyclopedia of genes and genomes. Nucleic Acids Res. 2000;28:27–30. 10.1093/nar/28.1.27 PMC102409 10592173

[52] Tomayko MM, Reynolds CP. Determination of subcutaneous tumor size in athymic (nude) mice. Cancer Chemother Pharmacol. 1989;24:148–54. 10.1007/BF00300234 2544306

[53] Dell RB, Holleran S, Ramakrishnan R. Sample size determination. ILAR J. 2002;43:207–13. 10.1093/ilar.43.4.207 PMC3275906 12391396

