# Supplementary material for: LINC01929 promotes breast cancer progression through a TFRC-associated ferroptosis pathway
Source: Cell Death Discov. 2026 Jul 9;12:396. doi: 10.1038/s41420-026-03248-y (PMC13631369; doi:10.1038/s41420-026-03248-y)

Figure 5e

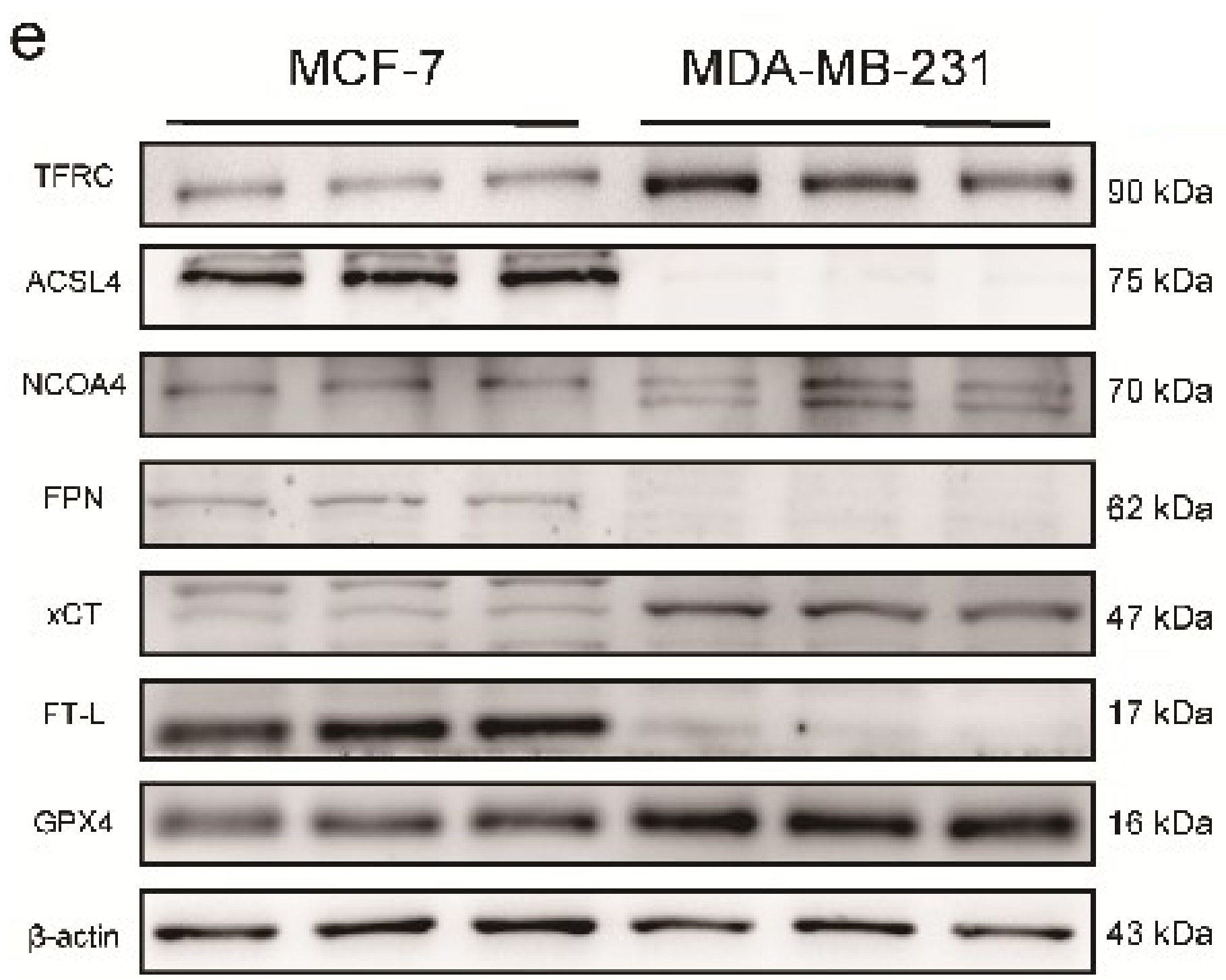
e
MCF-7
MDA-MB-231
TFRC
ACSL4
NCOA4
FPN
xCT
FT-L
GPX4
β-actin
90 kDa
75 kDa
70 kDa
62 kDa
47 kDa
17 kDa
16 kDa
43 kDa

Figure 5e—TFRC-90 kDa

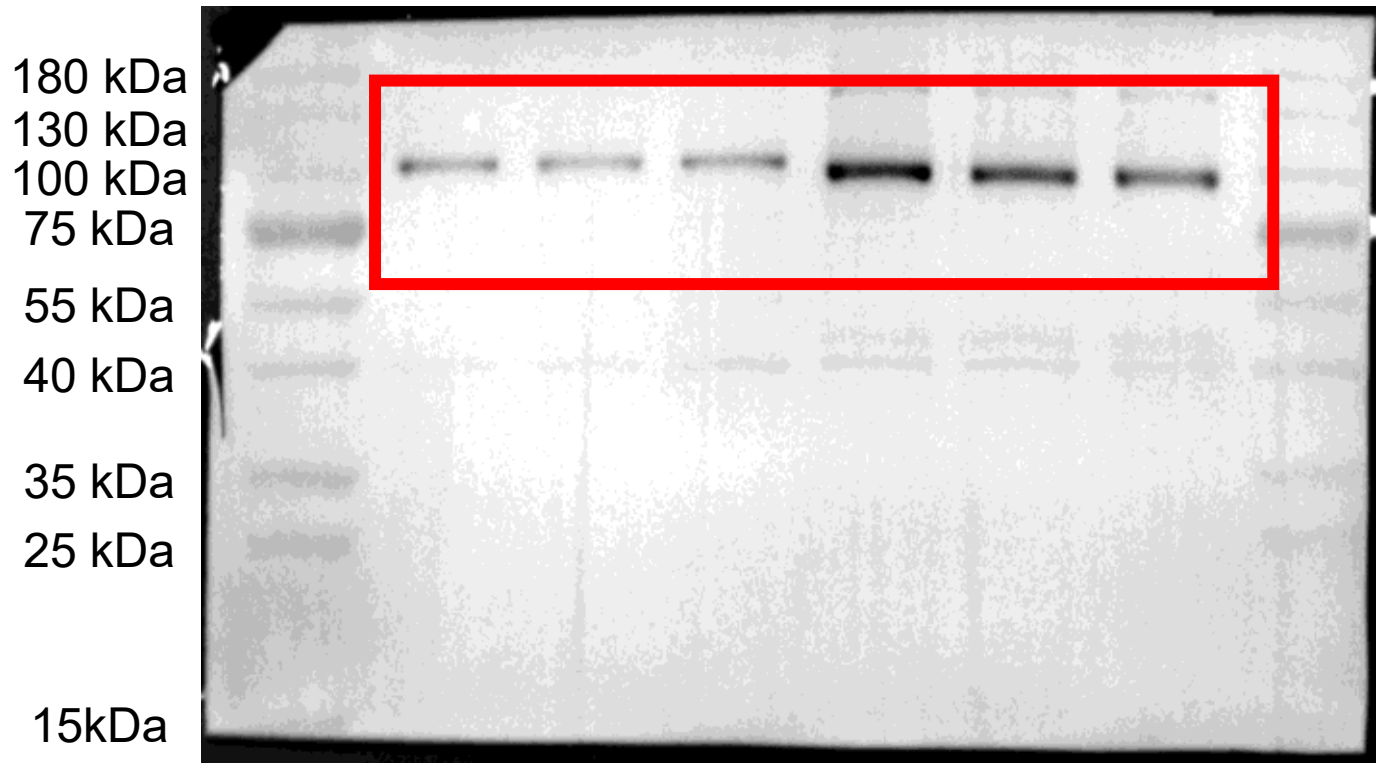

Figure 5e -ACSL4- 75 kDa

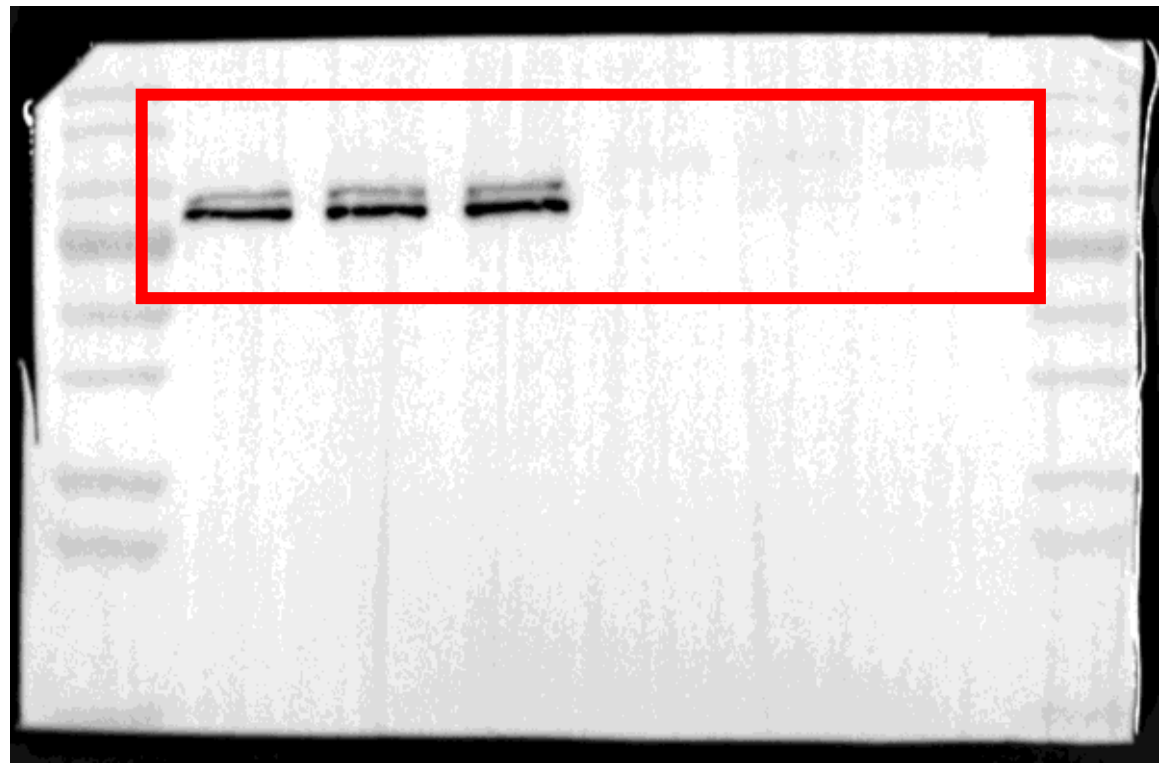

Figure 5e -NCOA4-70 kDa

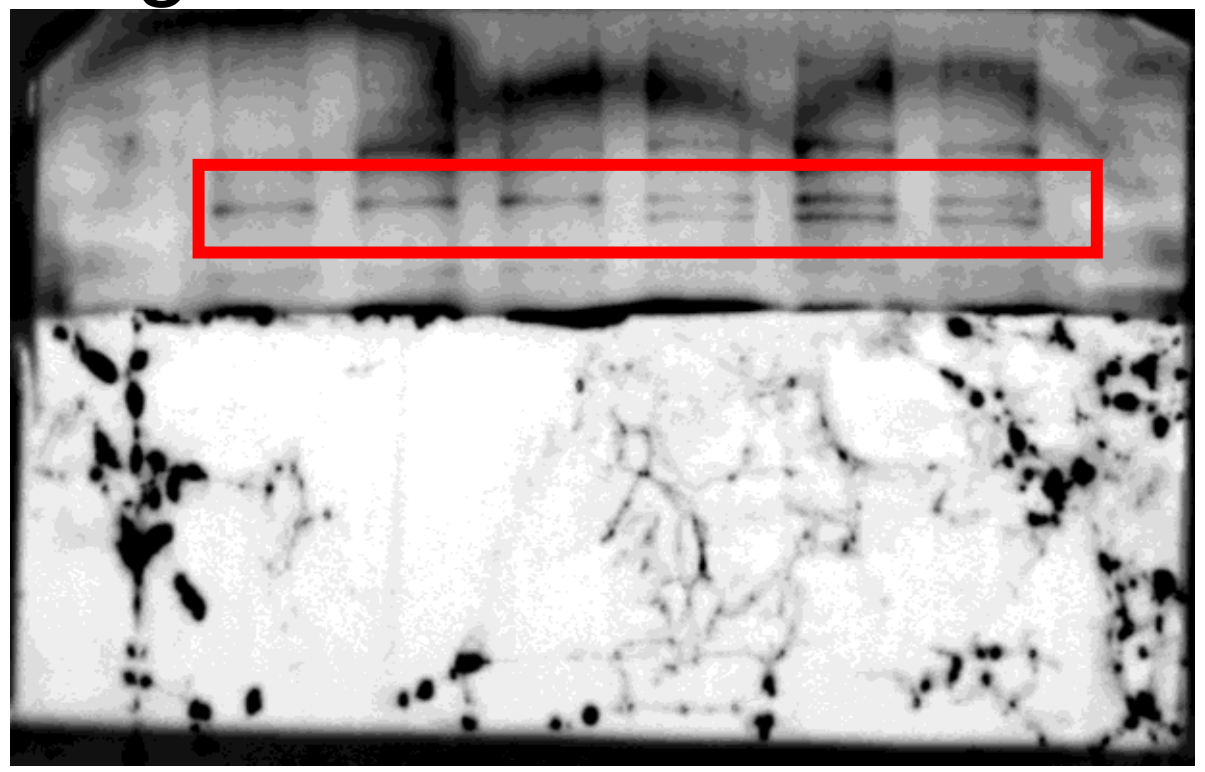

Figure 5e -FPN-62 kDa

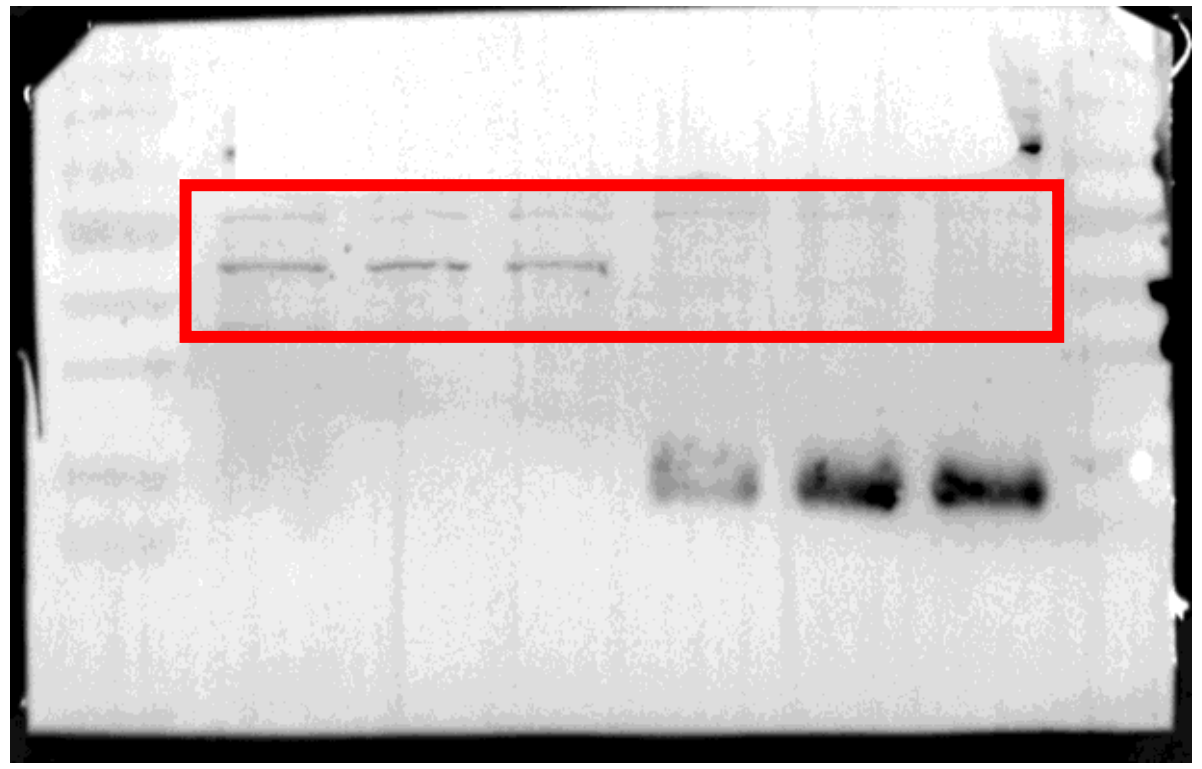

Figure 5e -xCT-47 kDa

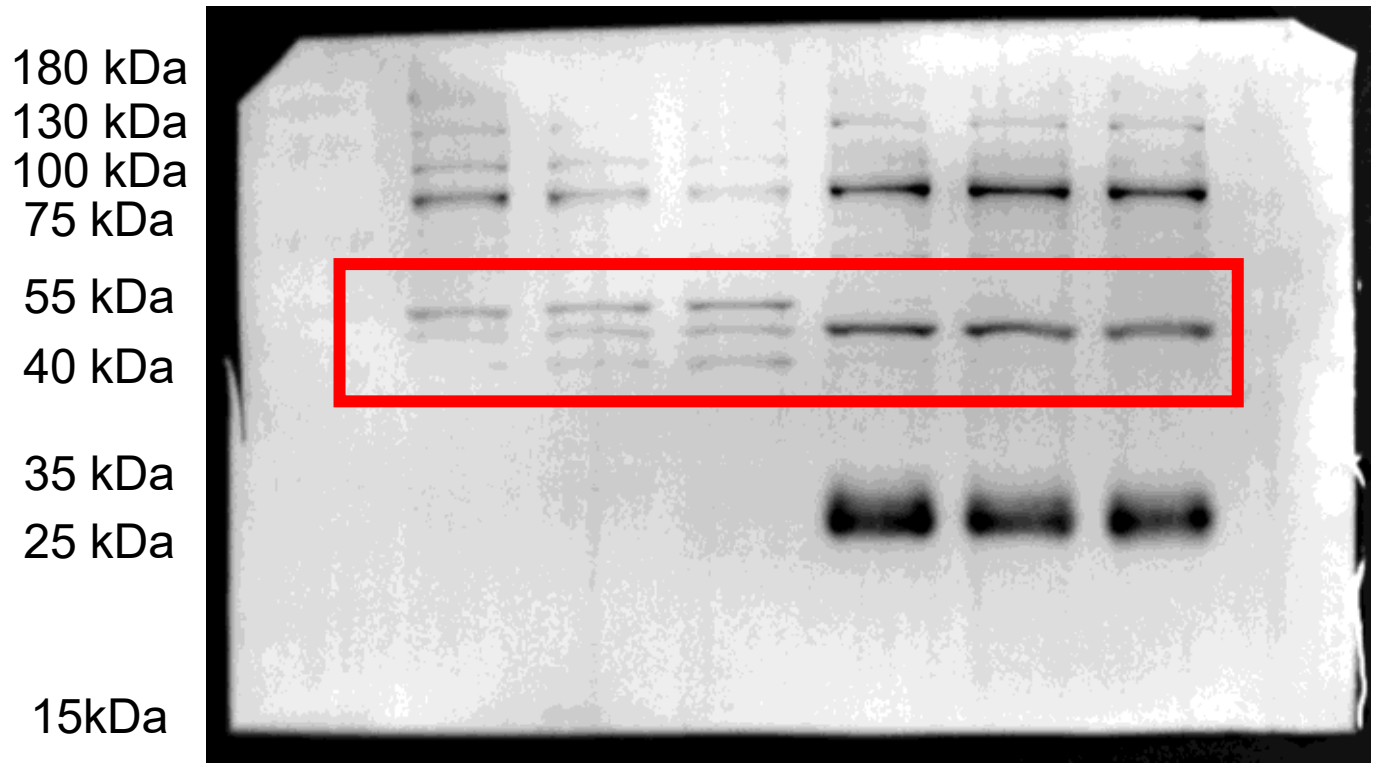

Figure 5e -FT-L-17kDa

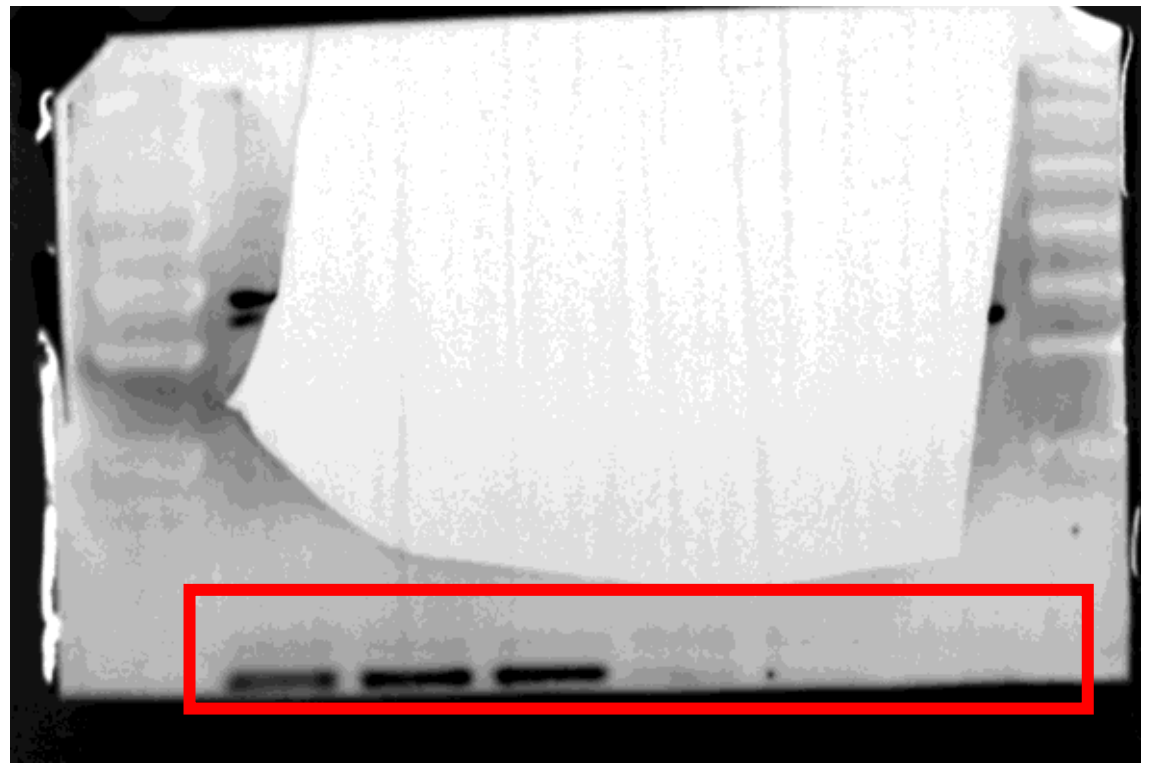

Figure 5e -GPX4-16 kDa

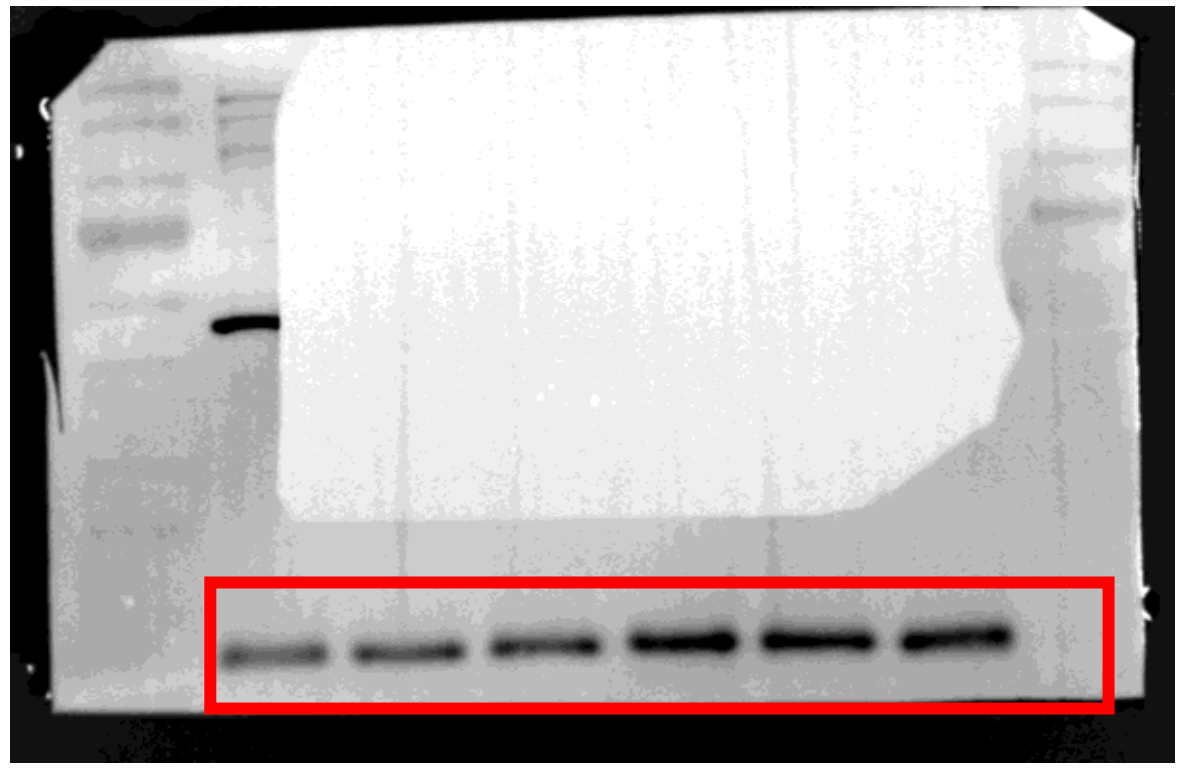

Figure 5e--β-actin--43 kDa

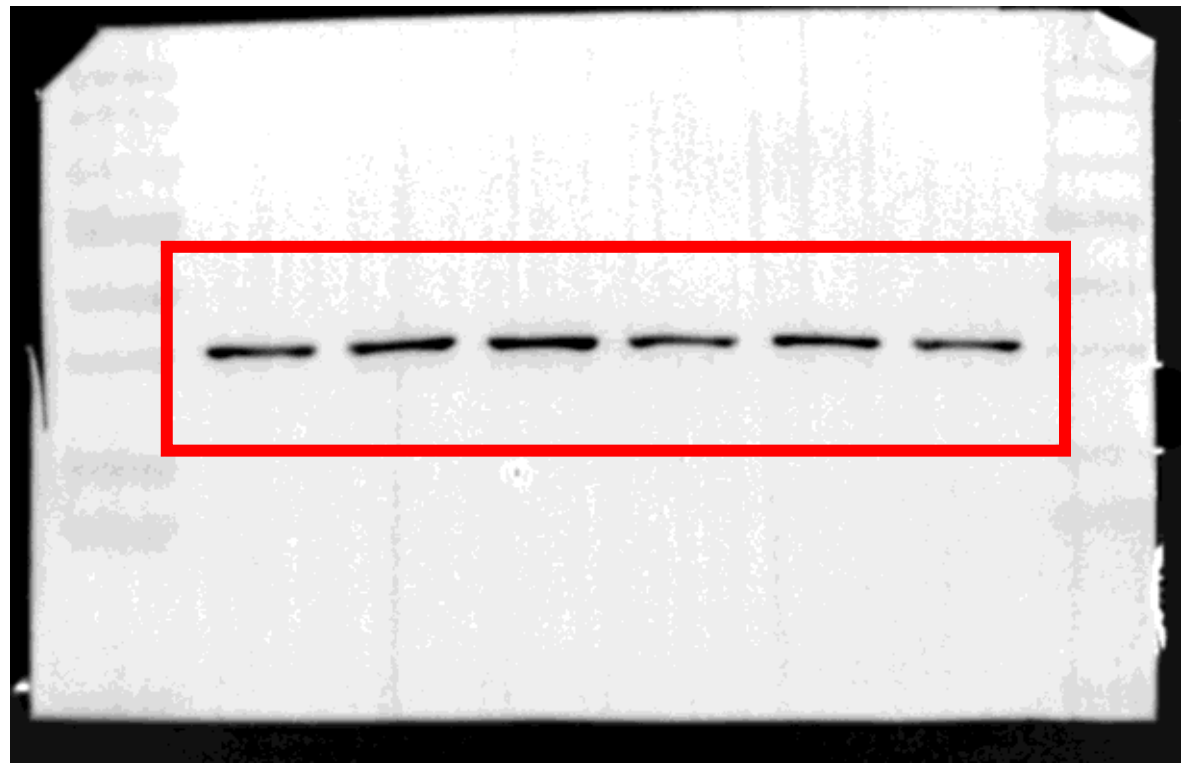

Figure 5g

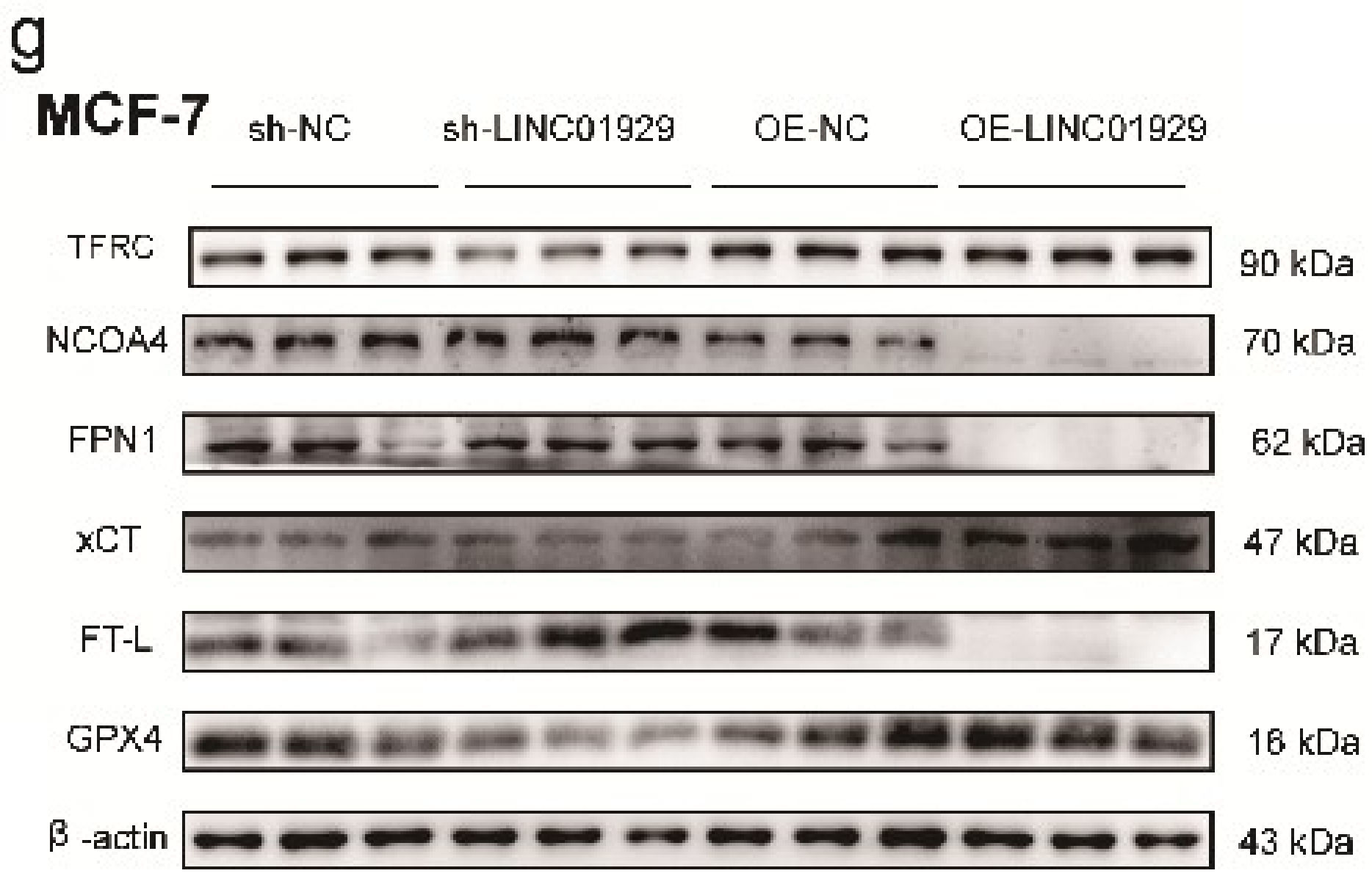
g
MCF-7
sh-NC
sh-LINC01929
OE-NC
OE-LINC01929
TFRC
NCOA4
FPN1
xCT
FT-L
GPX4
β -actin
90 kDa
70 kDa
62 kDa
47 kDa
17 kDa
16 kDa
43 kDa

Figure 5g—TFRC-90 kDa

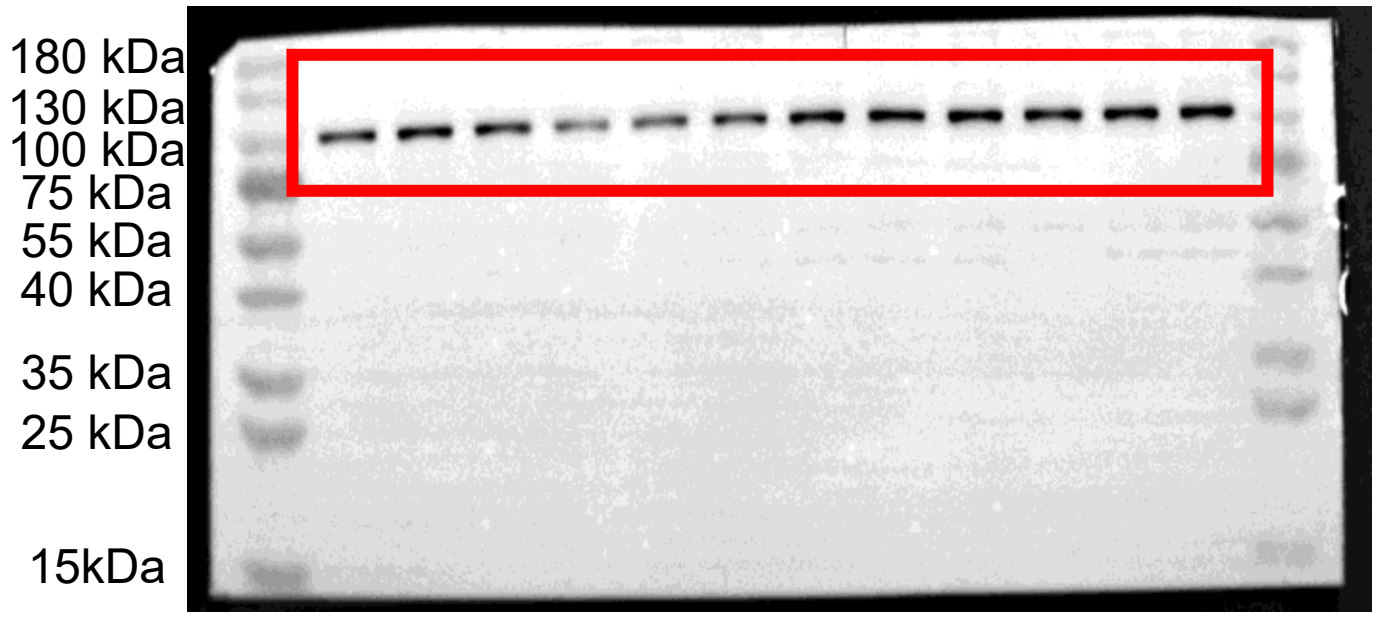

Figure 5g -NCOA4-70 kDa

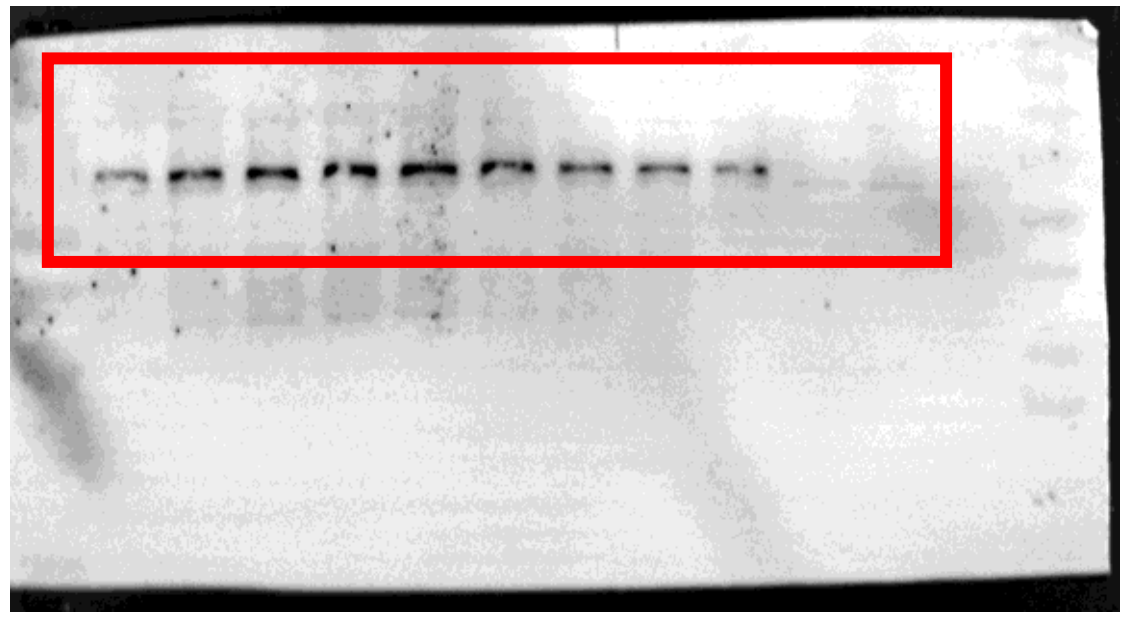

Figure 5g –FPN1-62 kDa

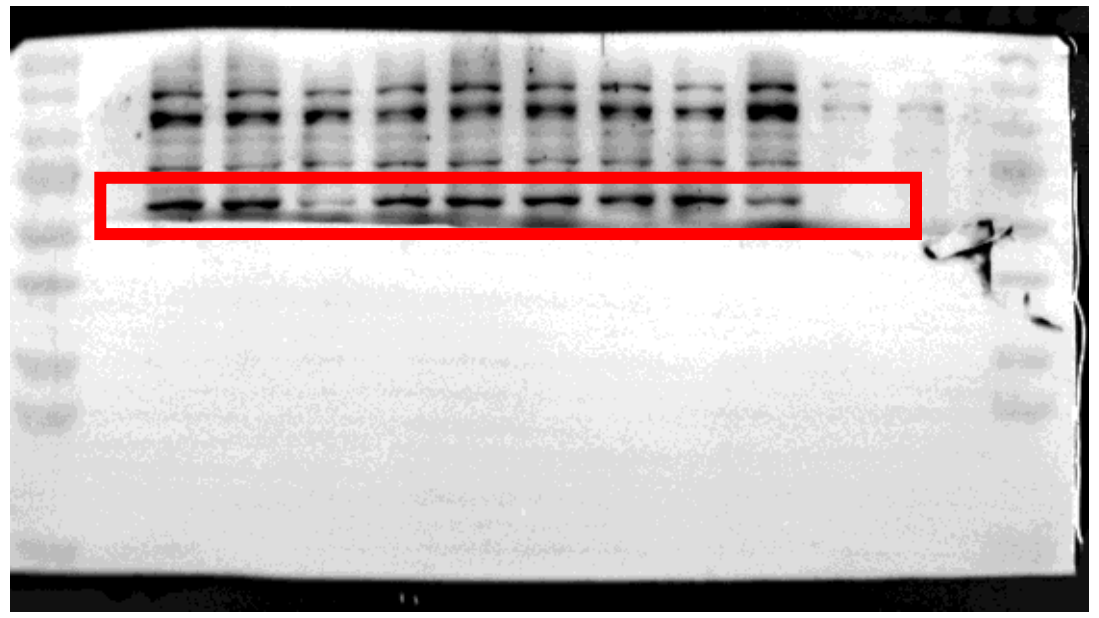

Figure 5g -xCT-47 kDa

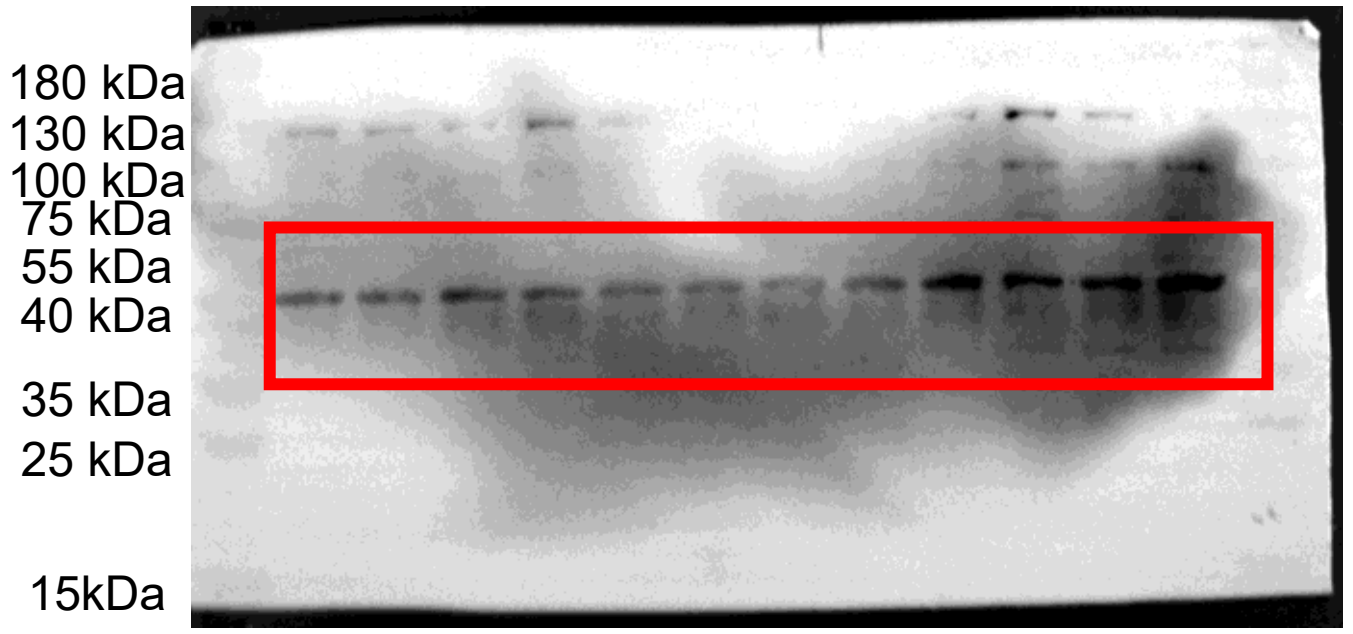

Figure 5g-FT-L-17kDa

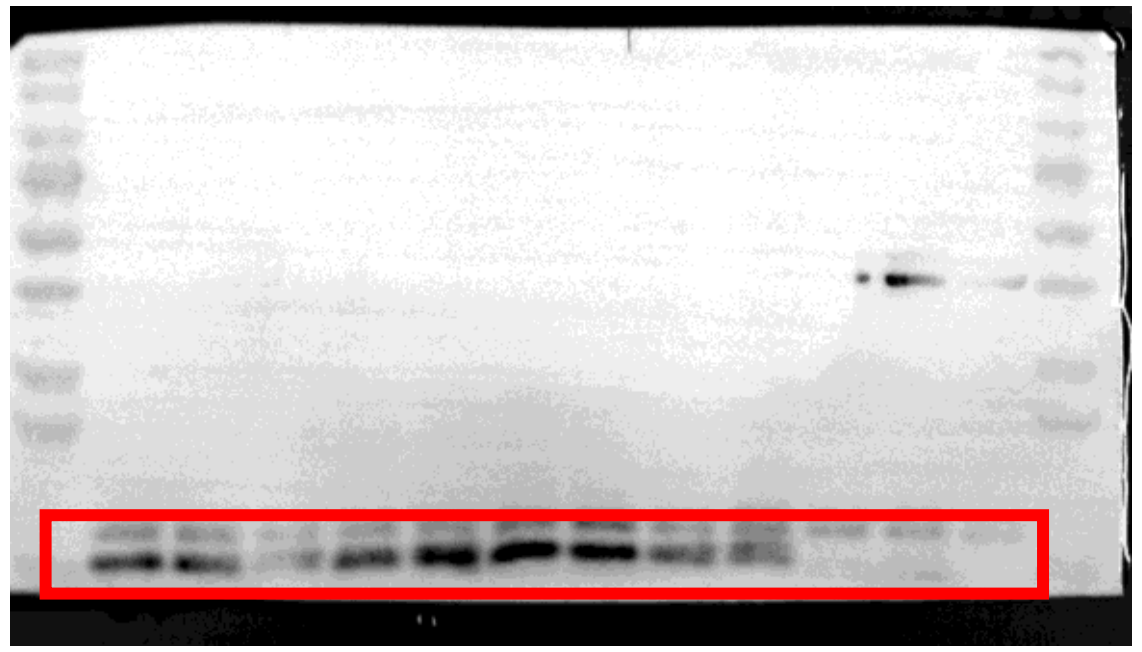

Figure 5g -GPX4-16 kDa

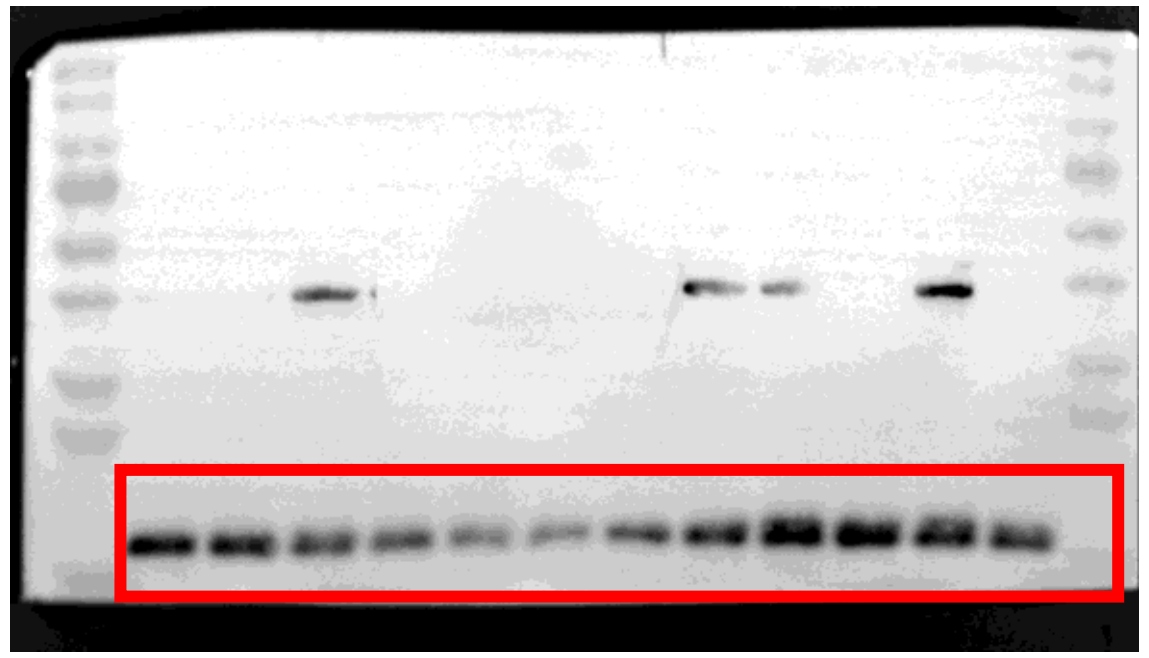

Figure 5g--β-actin--43 kDa

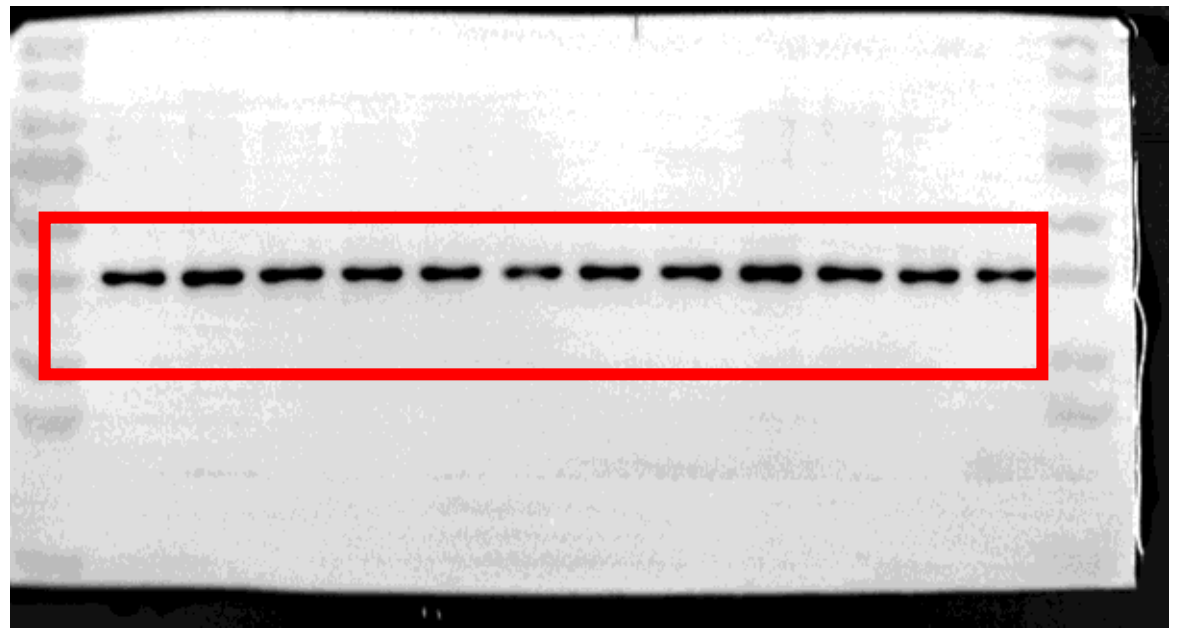

Figure 5i

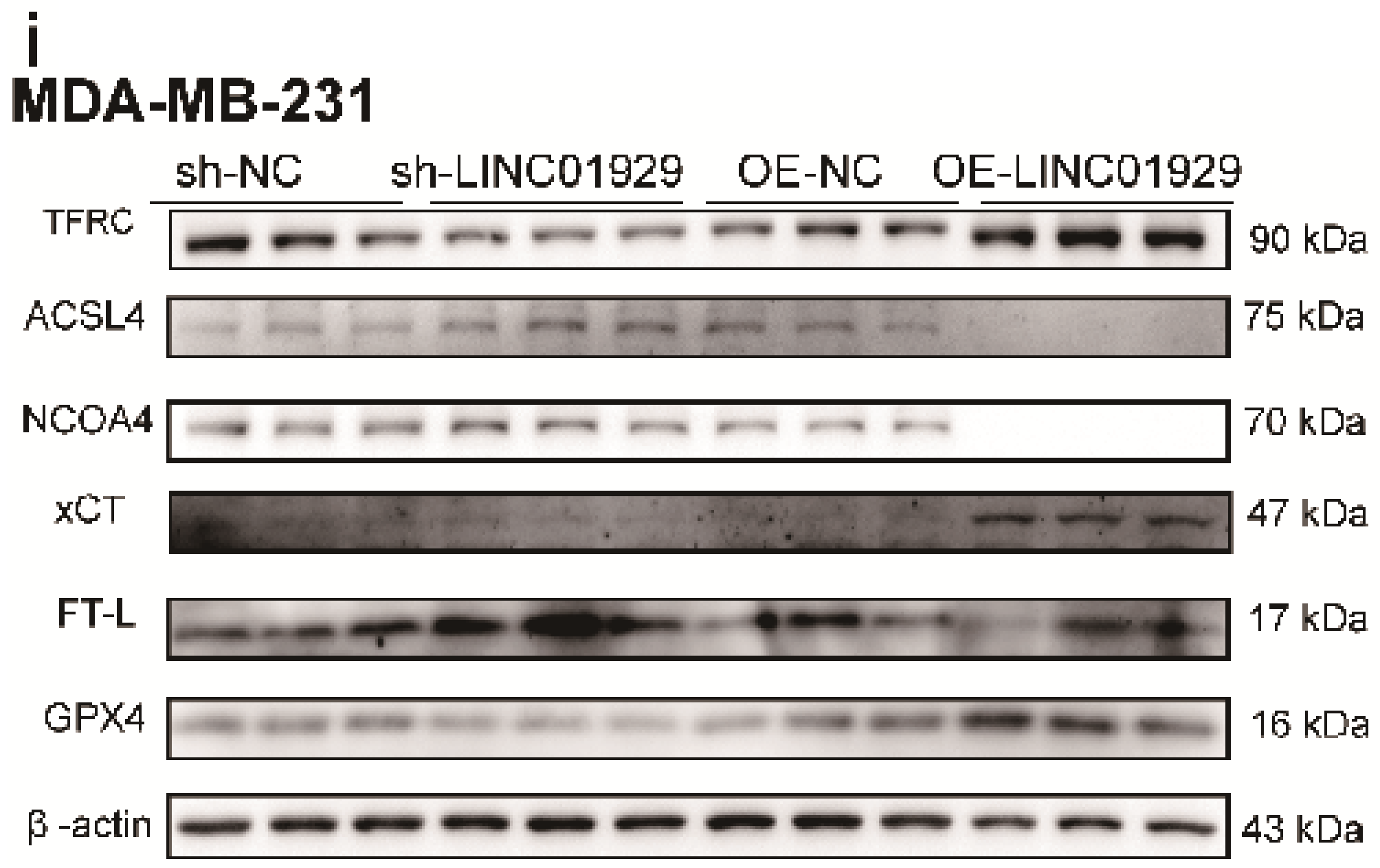
i
MDA-MB-231
sh-NC
sh-LINC01929
OE-NC
OE-LINC01929
TFRC
ACSL4
NCOA4
xCT
FT-L
GPX4
β -actin
90 kDa
75 kDa
70 kDa
47 kDa
17 kDa
16 kDa
43 kDa

Figure 5i—TFRC-90 kDa

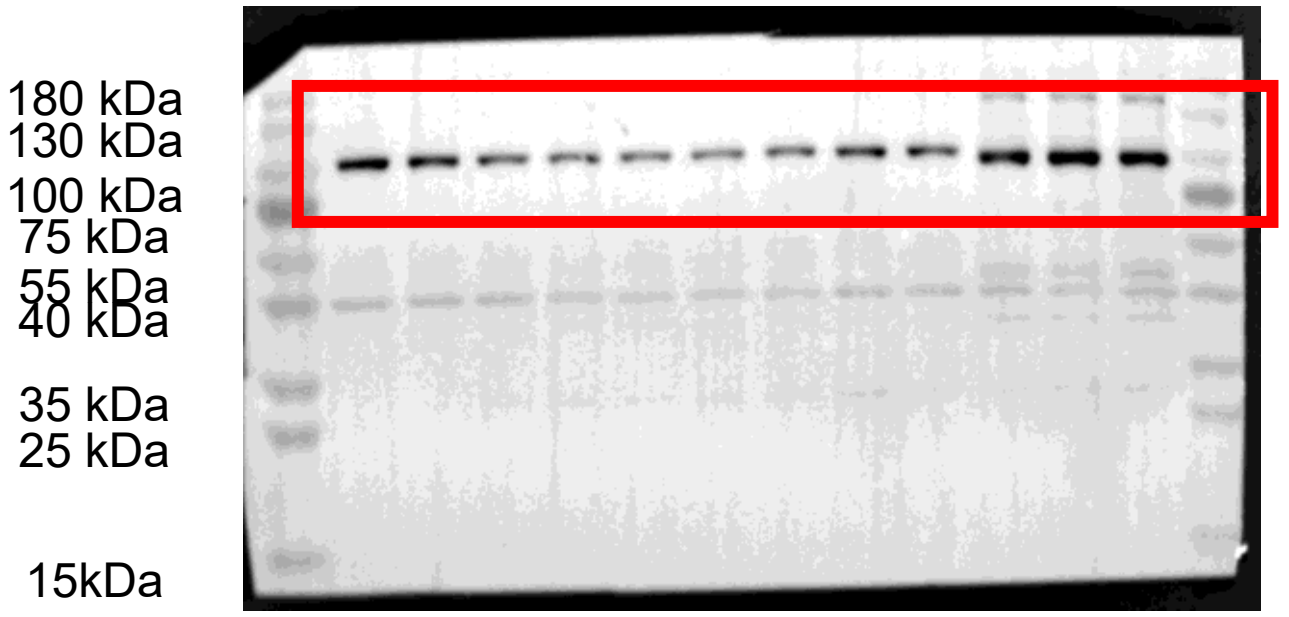

Figure 5i -ACSL4- 75 kDa

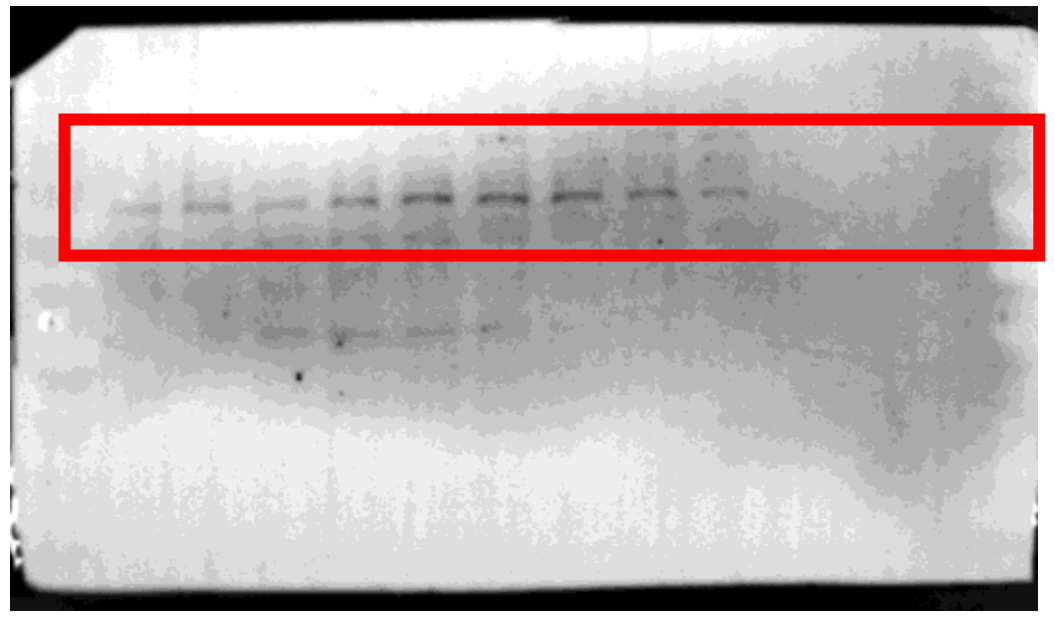

Figure 5i -NCOA4-70 kDa

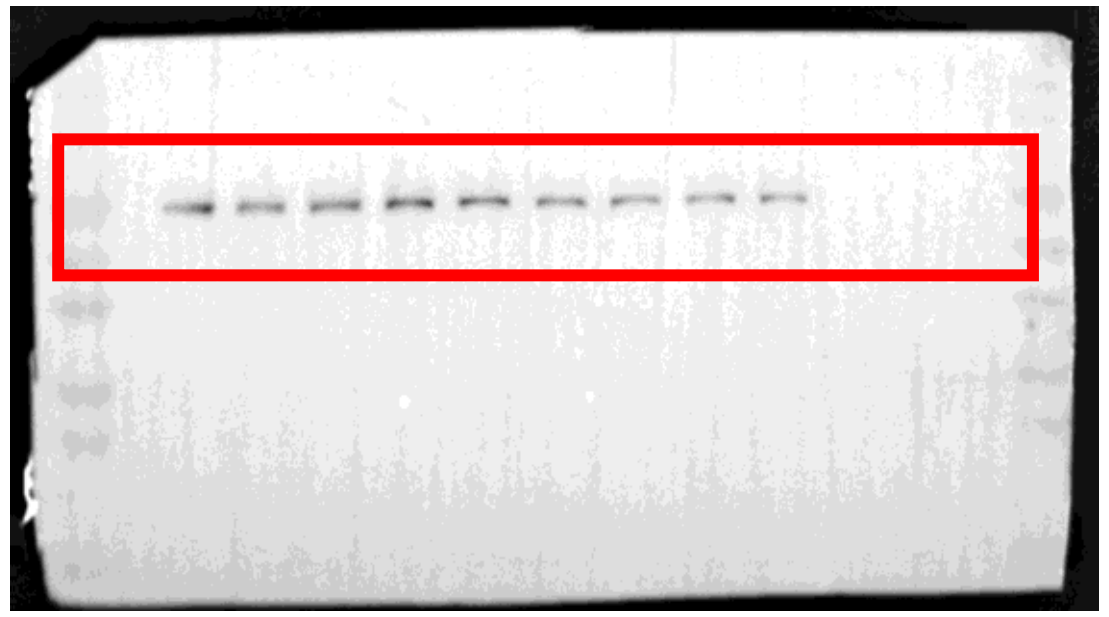

Figure 5i -xCT-47 kDa

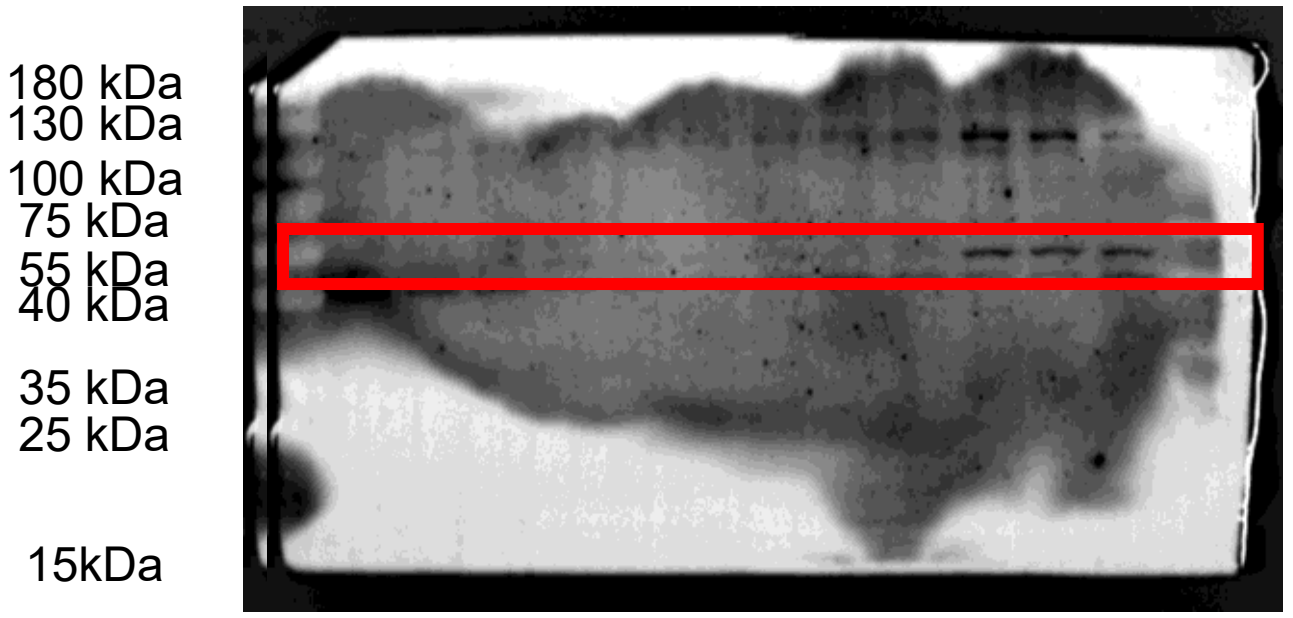

Figure 5i-FT-L-17kDa

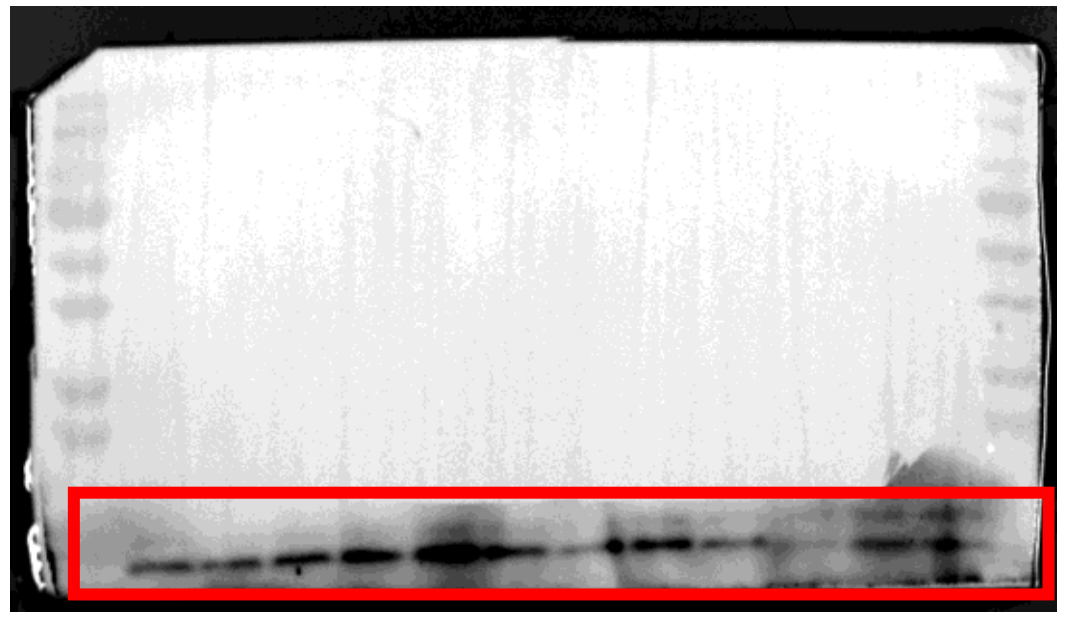

Figure 5i -GPX4-16 kDa

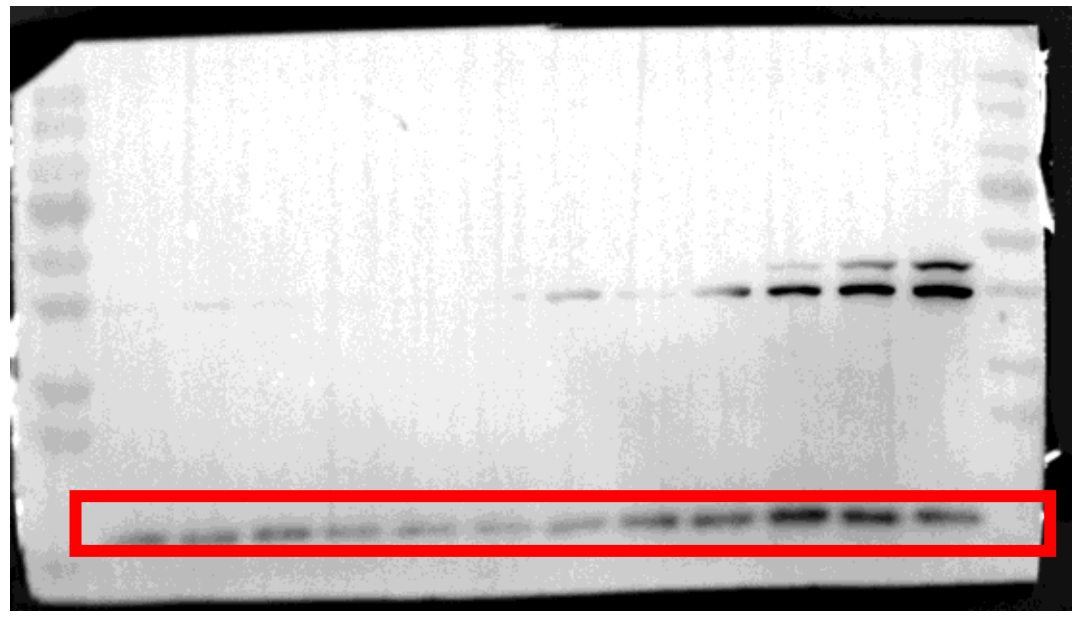

Figure 5i--β-actin--43 kDa

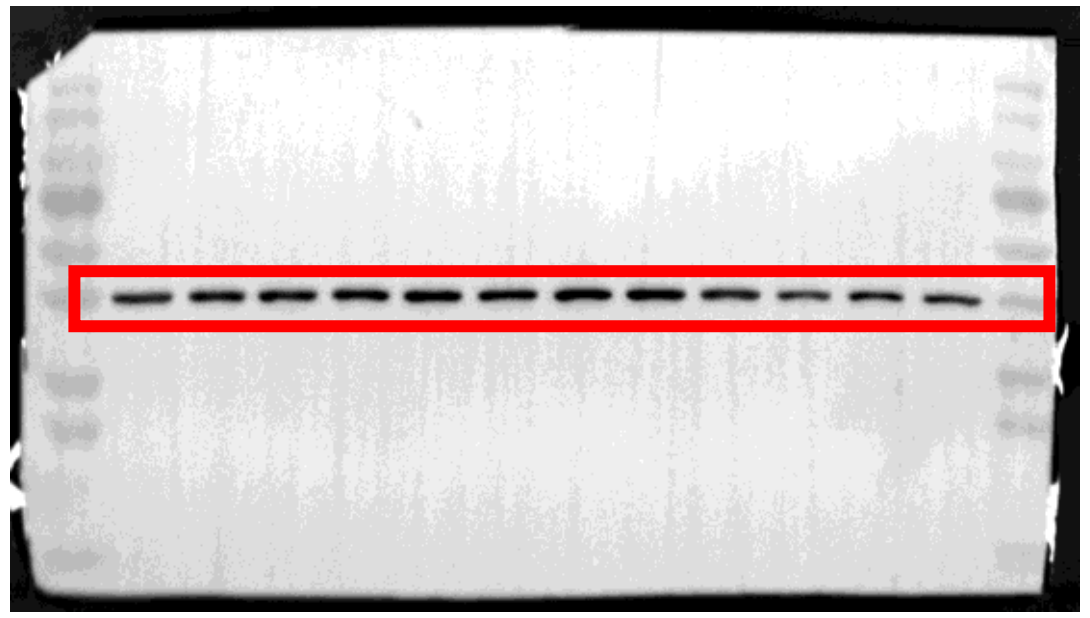

Figure S3a

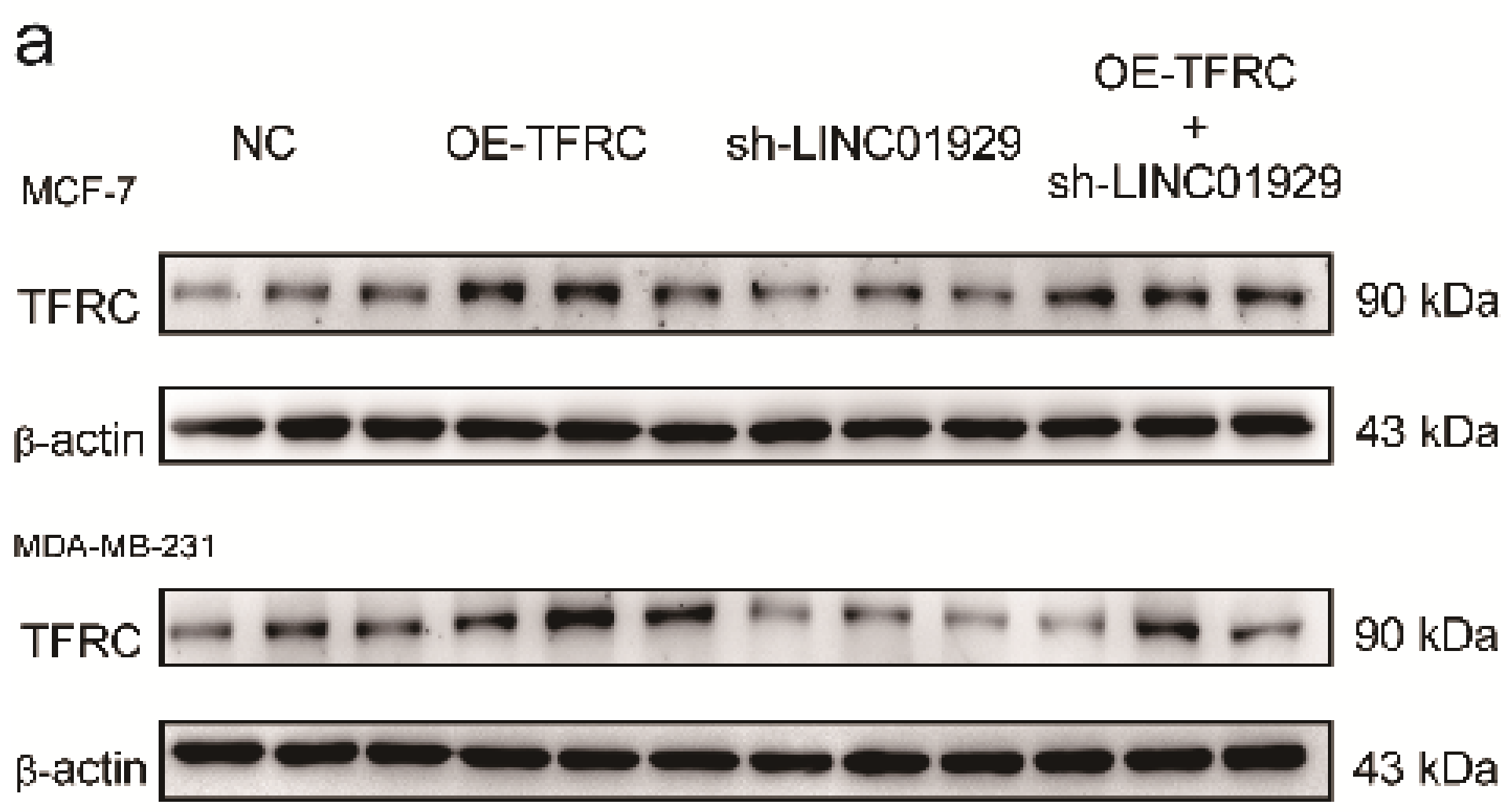
a
NC
OE-TFRC
sh-LINC01929
OE-TFRC
+
sh-LINC01929
MCF-7
TFRC
90 kDa
β-actin
43 kDa
MDA-MB-231
TFRC
90 kDa
β-actin
43 kDa

Figure S3a --TFRC-MCF -90 kDa

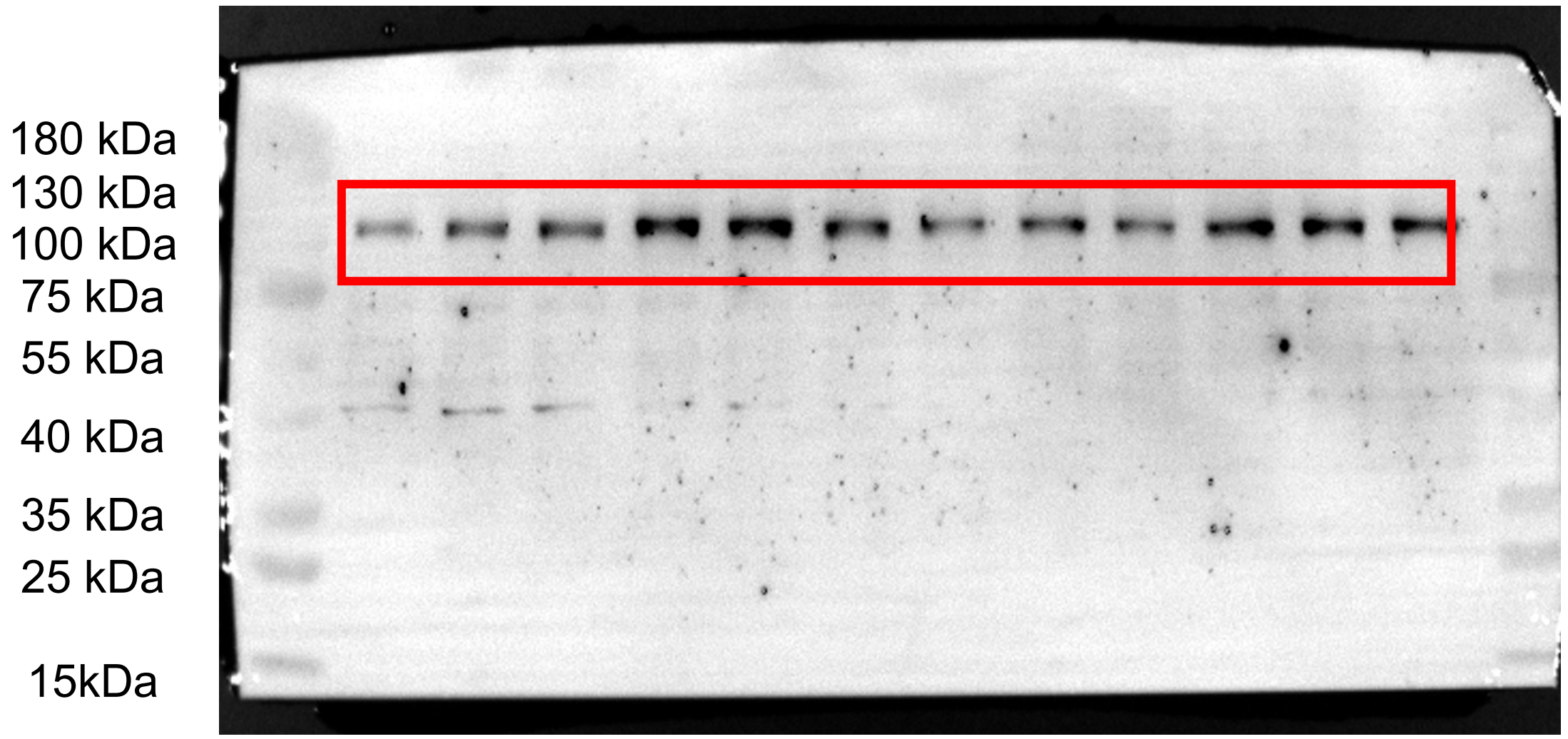

Figure S3a --β-actin-MCF -43 kDa

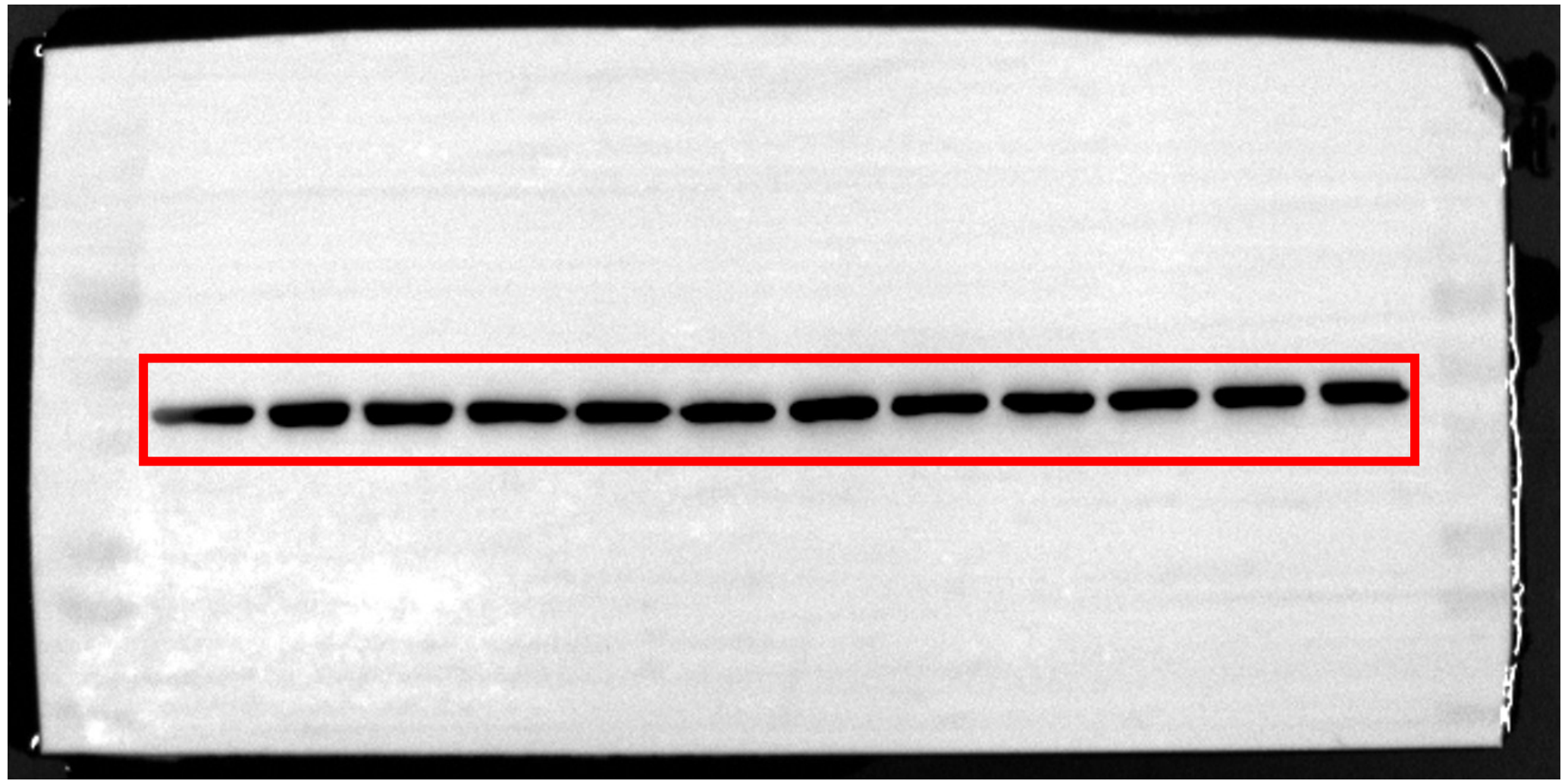

Figure S3a --TFRC-MDA -90 kDa

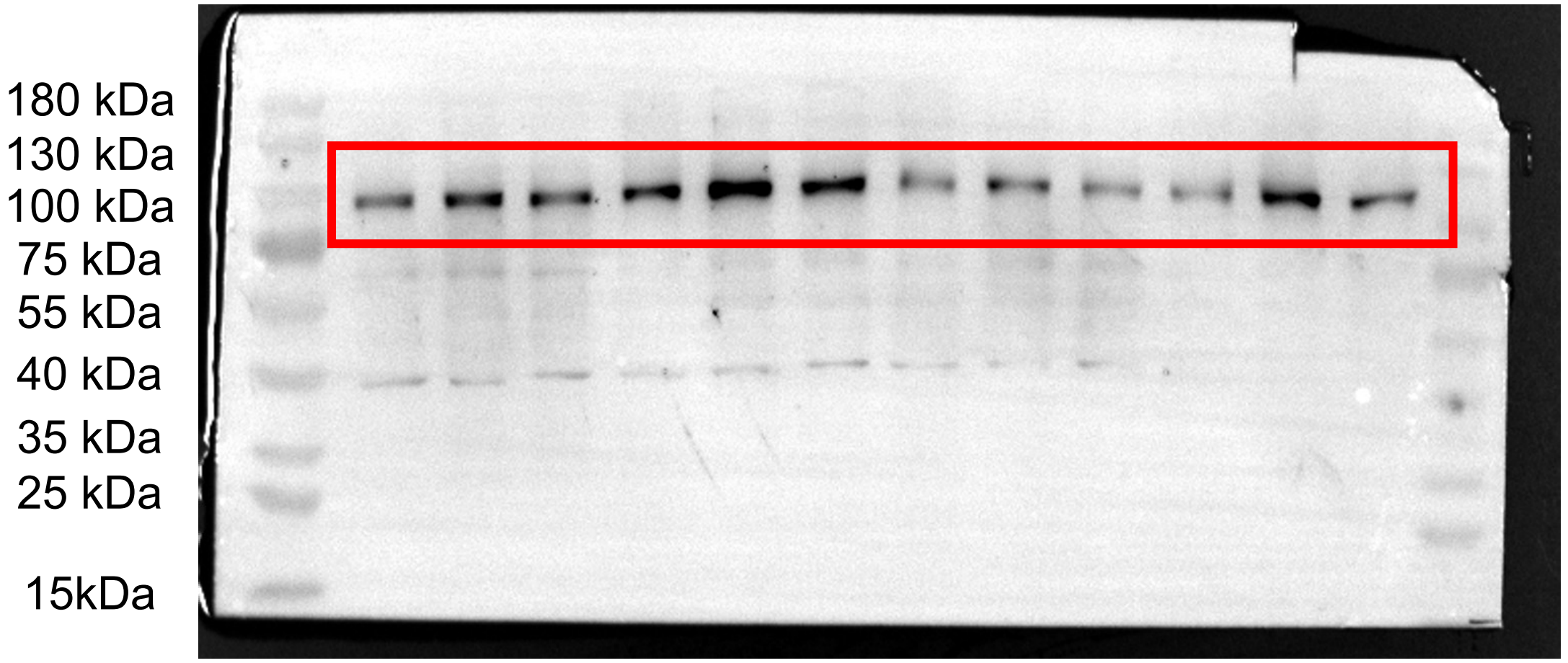

Figure S3a--β-actin-MDA -43 kDa

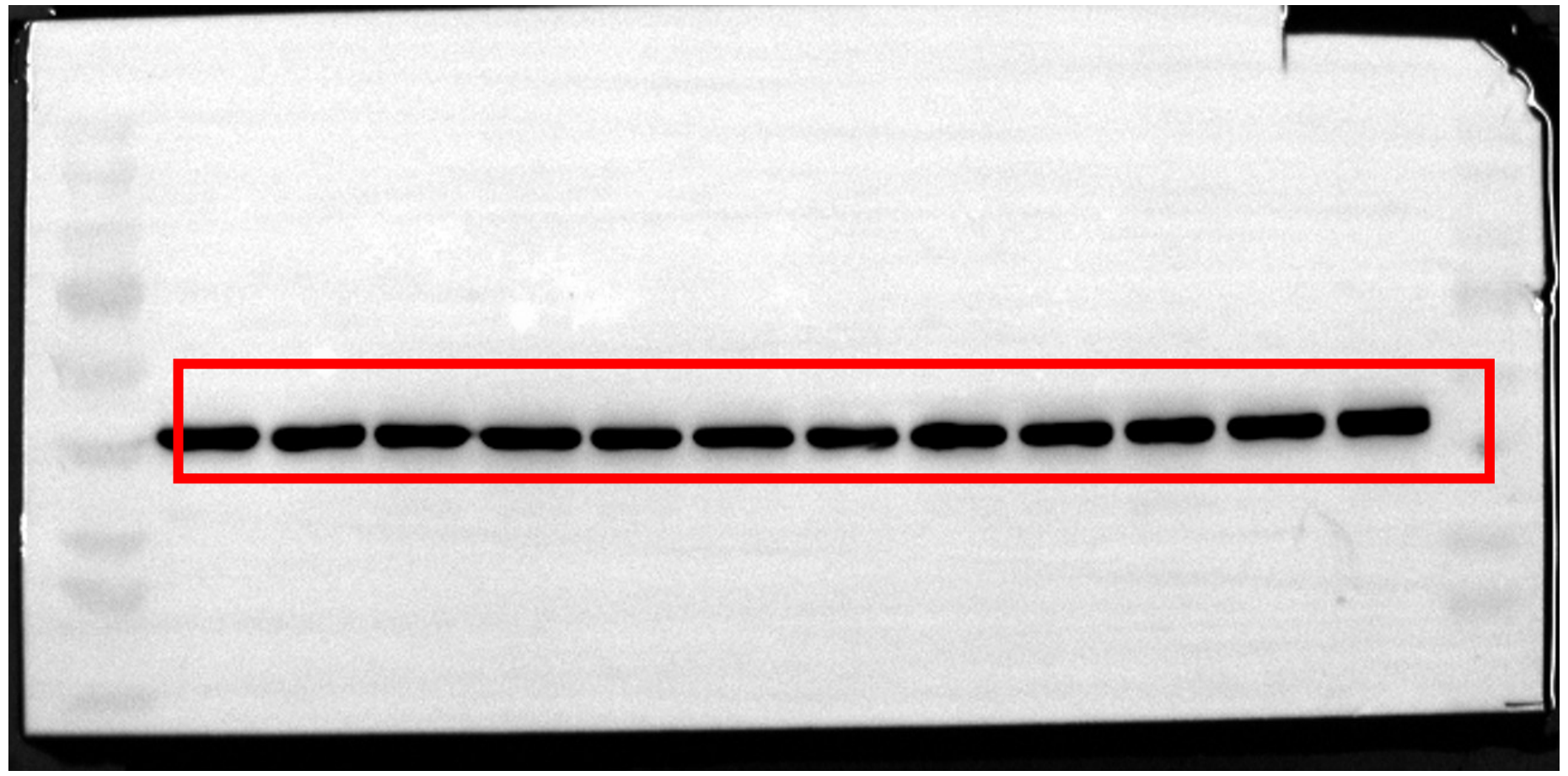

Figure S6f

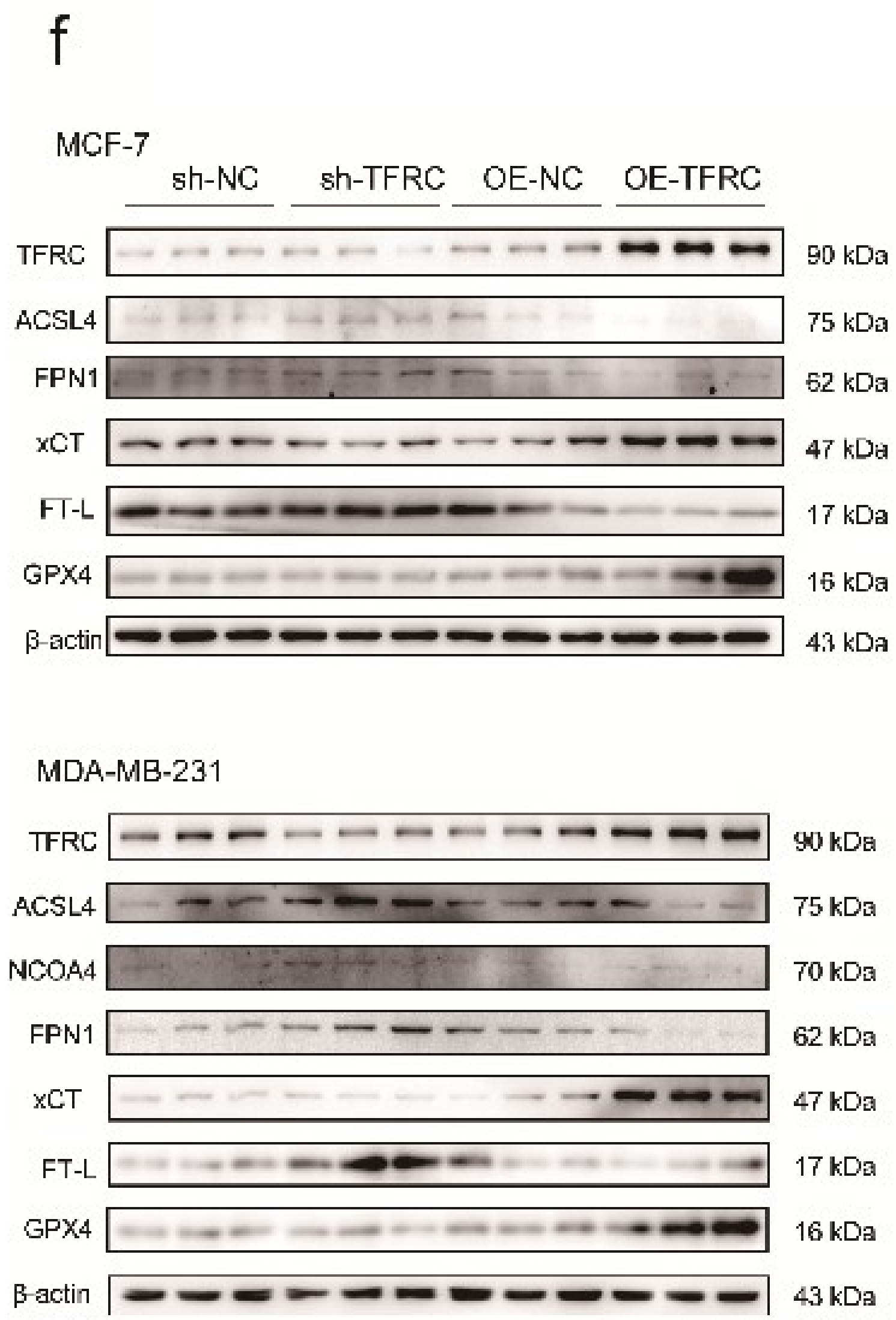
f
MCF-7
sh-NC
sh-TFRC
OE-NC
OE-TFRC
TFRC
90 kDa
ACSL4
75 kDa
FPN1
62 kDa
xCT
47 kDa
FT-L
17 kDa
GPX4
16 kDa
β-actin
43 kDa
MDA-MB-231
TFRC
90 kDa
ACSL4
75 kDa
NCOA4
70 kDa
FPN1
62 kDa
xCT
47 kDa
FT-L
17 kDa
GPX4
16 kDa
β-actin
43 kDa

MCF-7

Figure S6f—TFRC-90 kDa

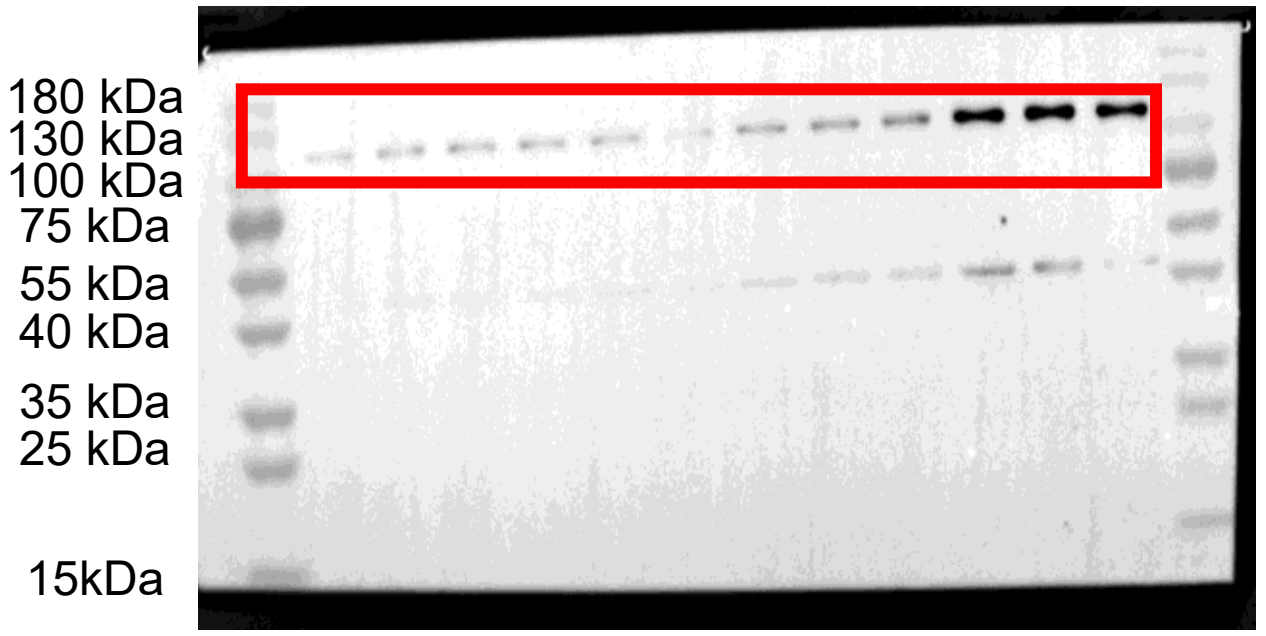

Figure S6f -ACSL4- 75 kDa

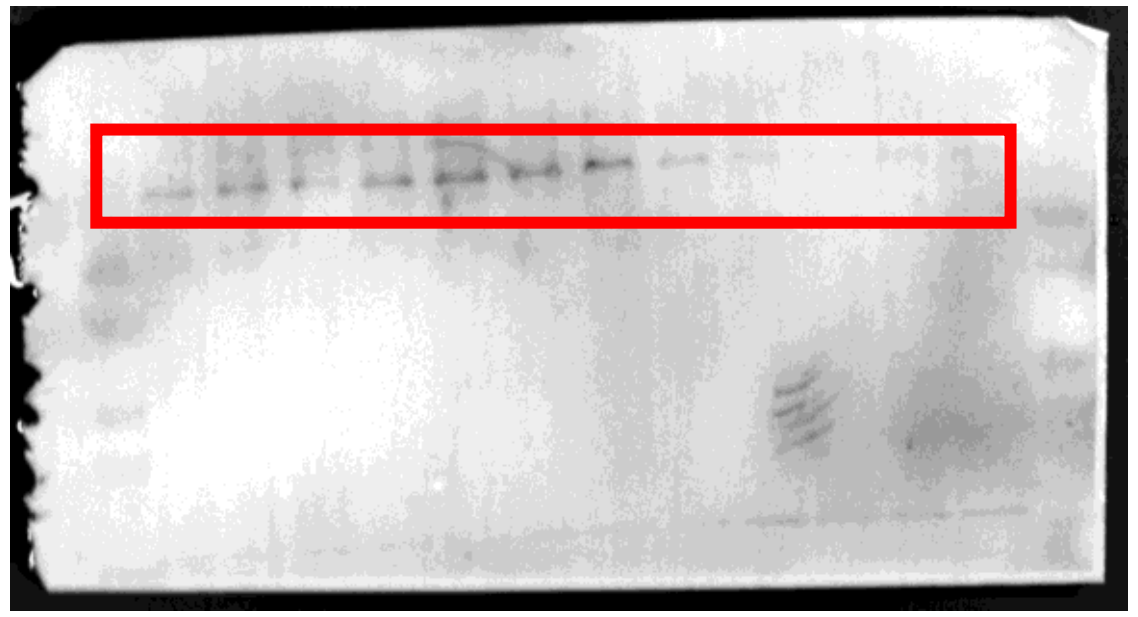

Figure S6f –FPN1-62 kDa

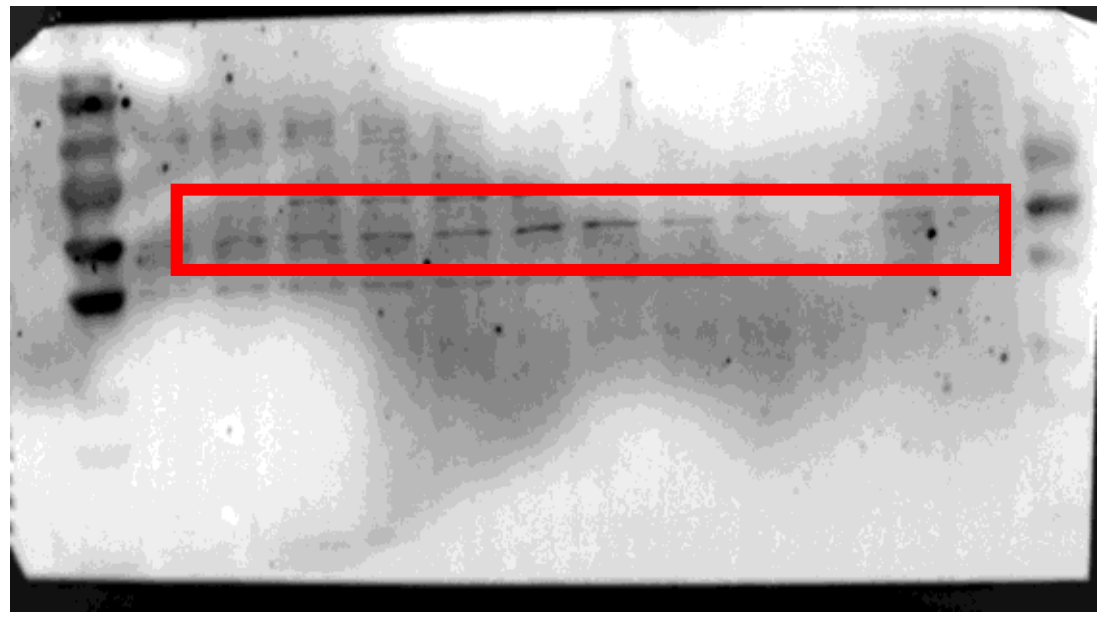

Figure S6f -xCT-47 kDa

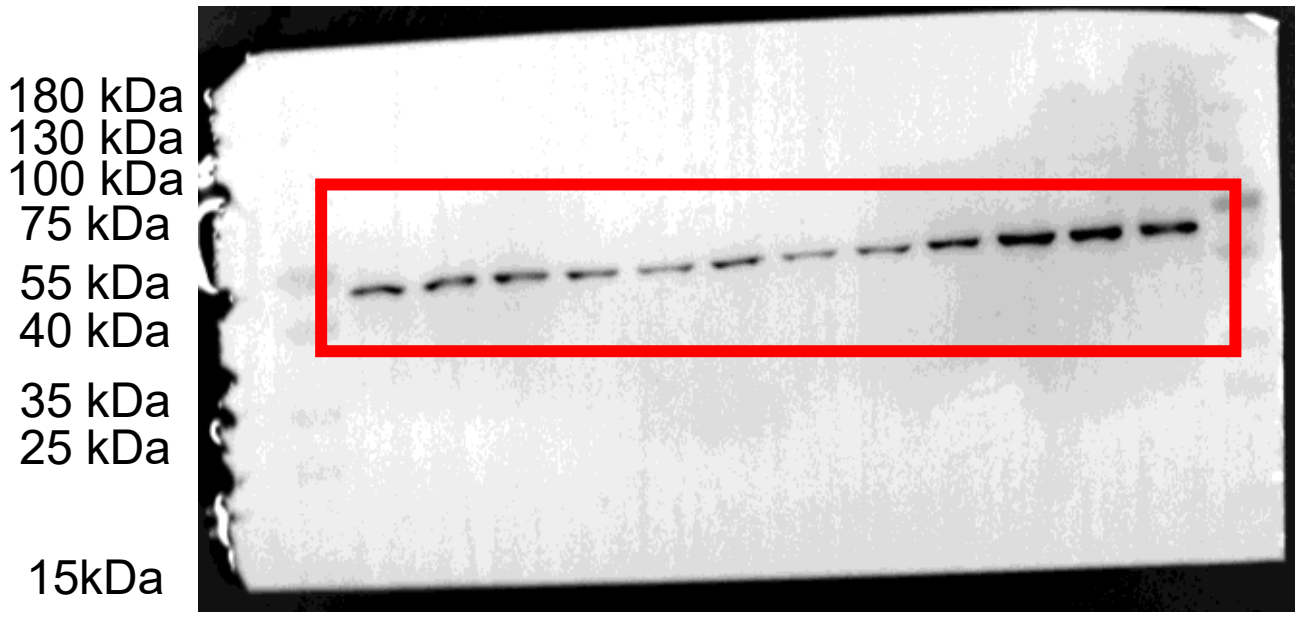

Figure S6f -FT-L-17kDa

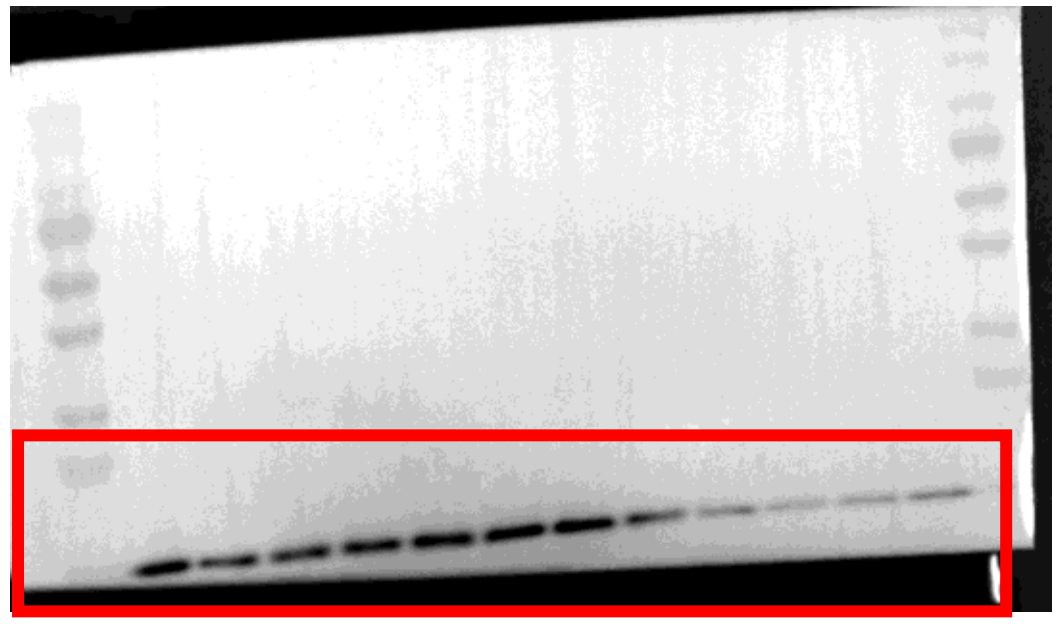

Figure S6f -GPX4-16 kDa

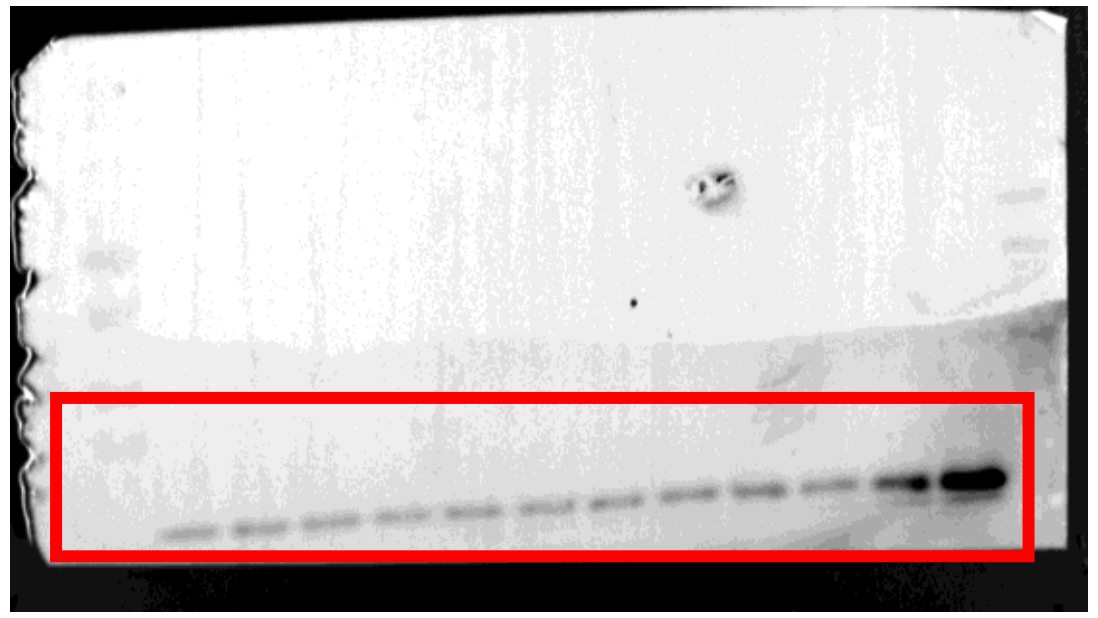

Figure S6f --β-actin--43 kDa

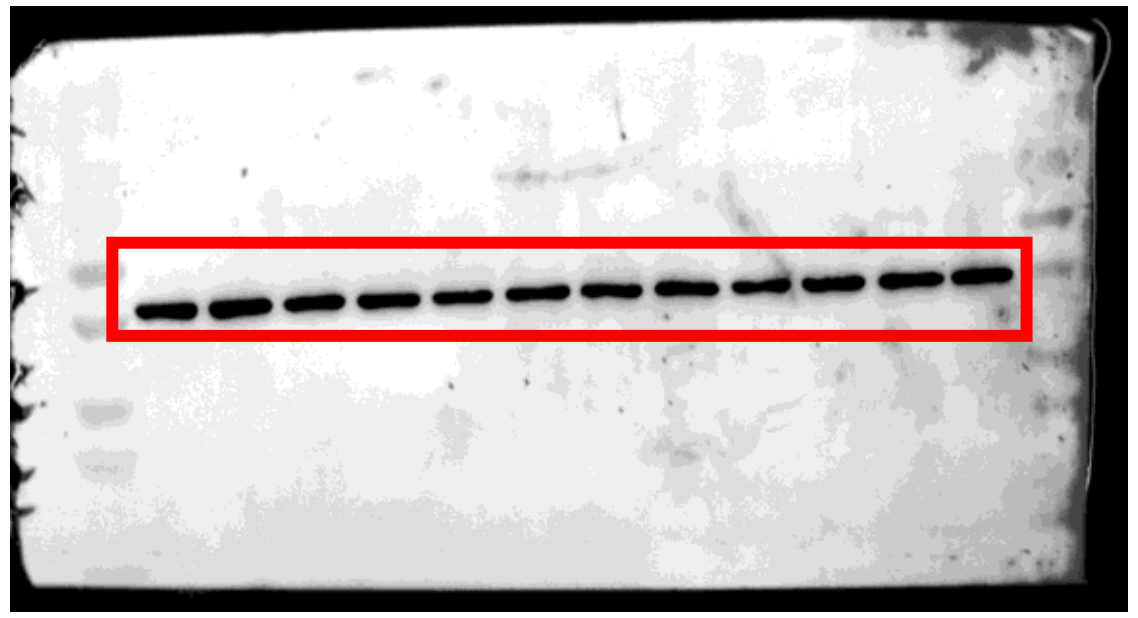

MDA-MB-231

Figure S6f—TFRC-90 kDa

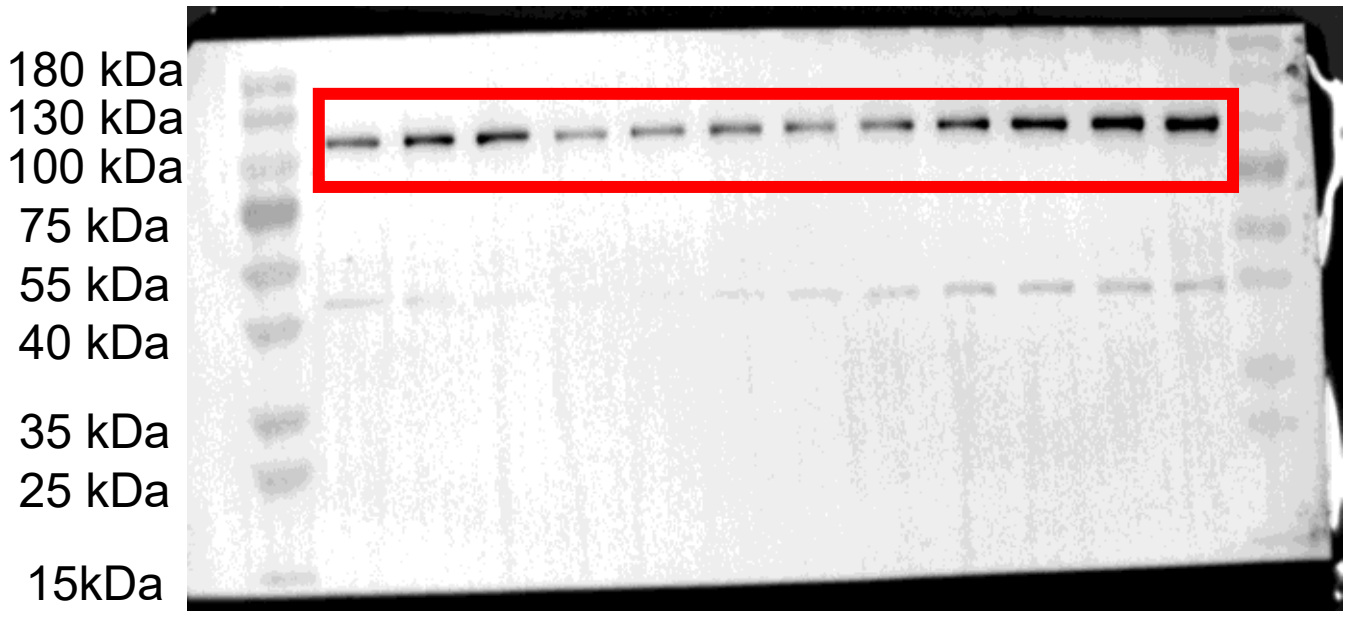

Figure S6f -ACSL4- 75 kDa

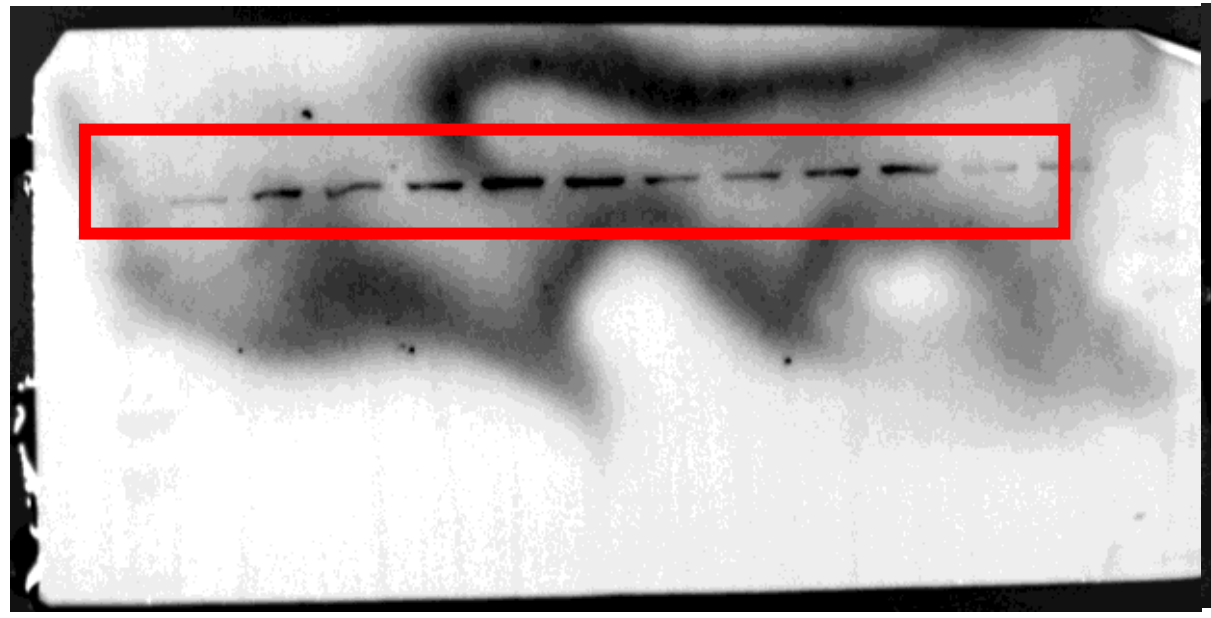

Figure S6f - NCOA4-70 kDa

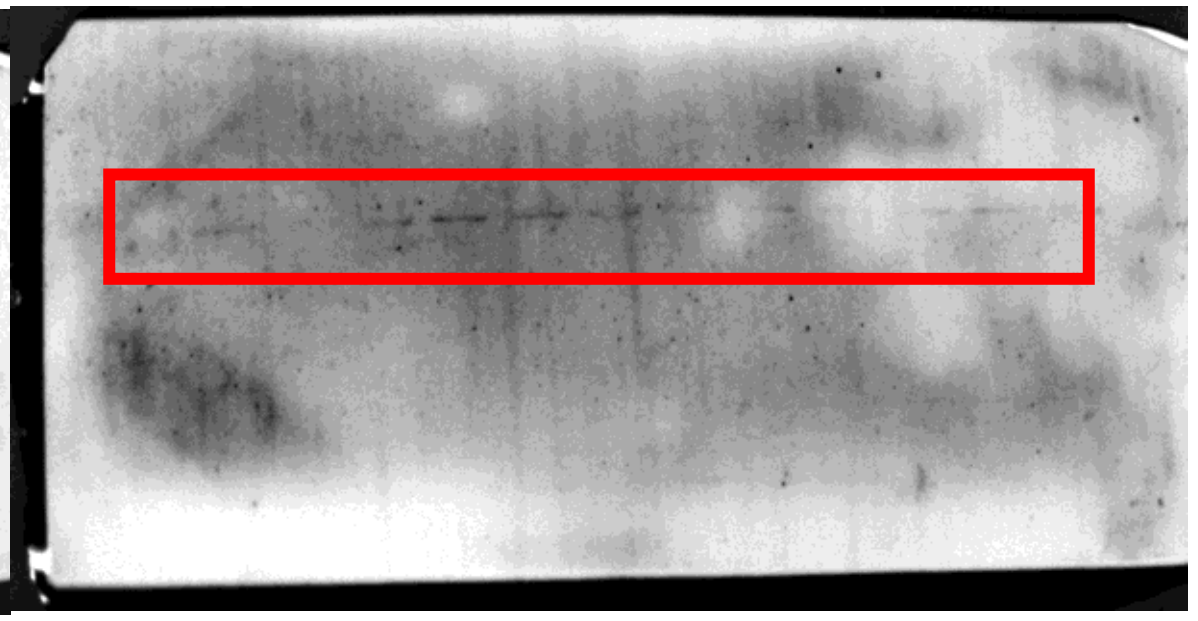

Figure S6f –FPN1-62 kDa

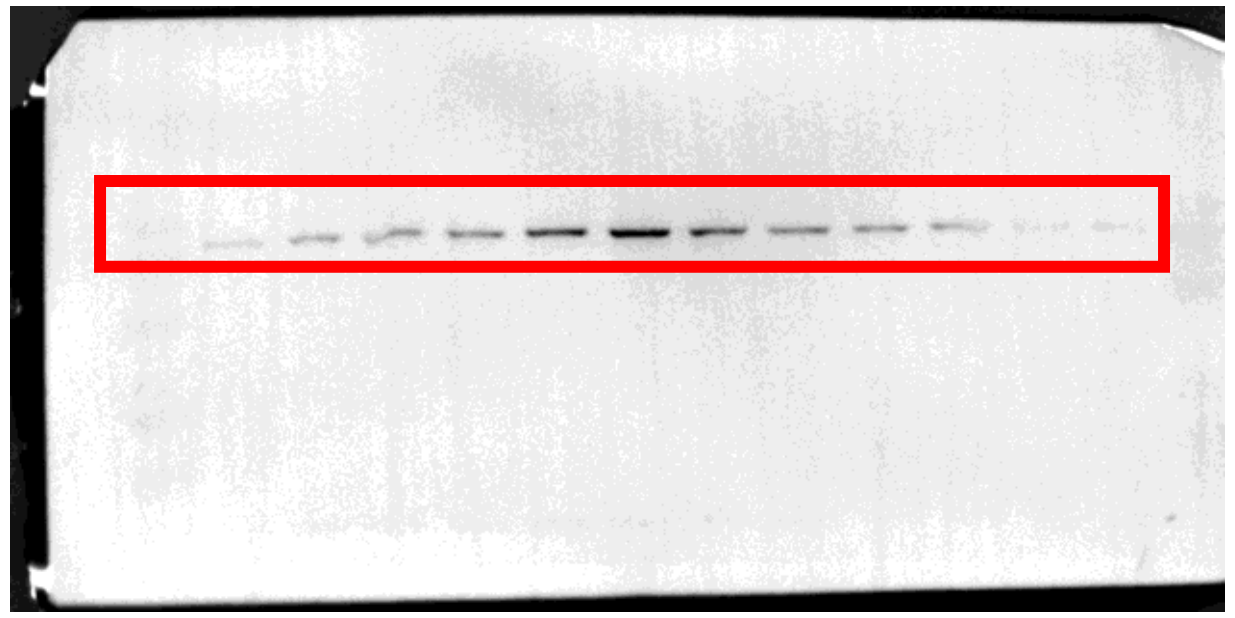

Figure S6f -xCT-47 kDa

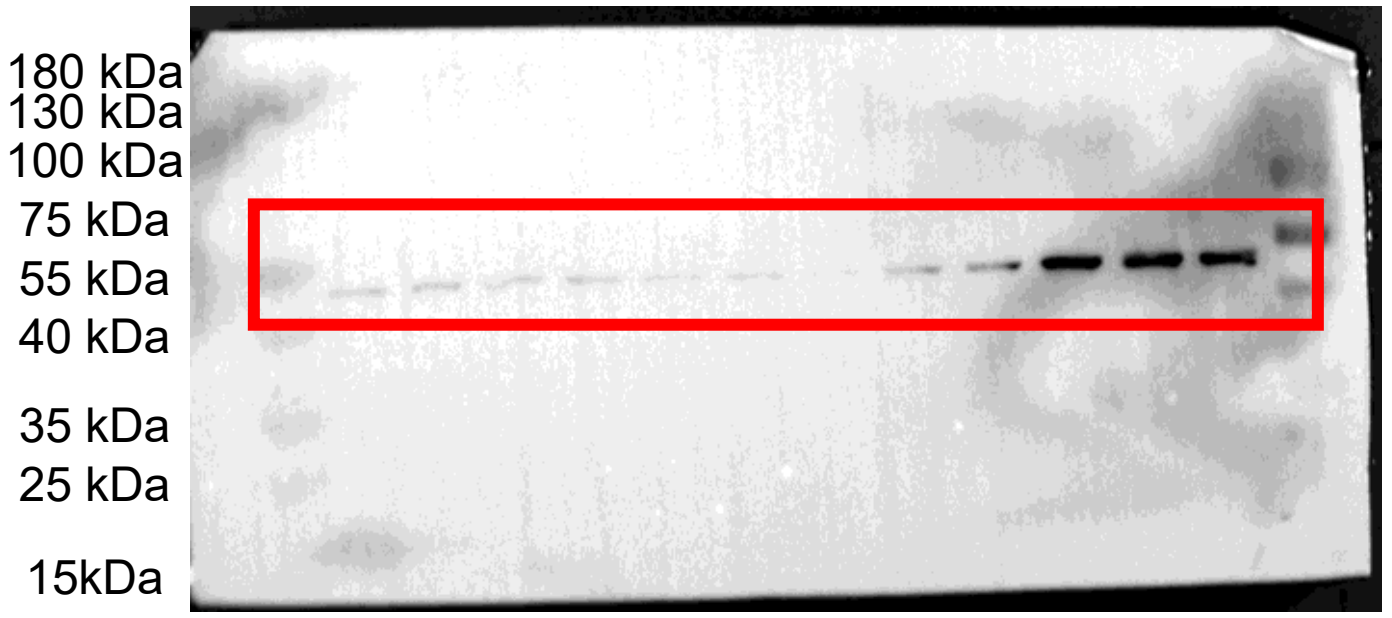

Figure S6f -FT-L-17kDa

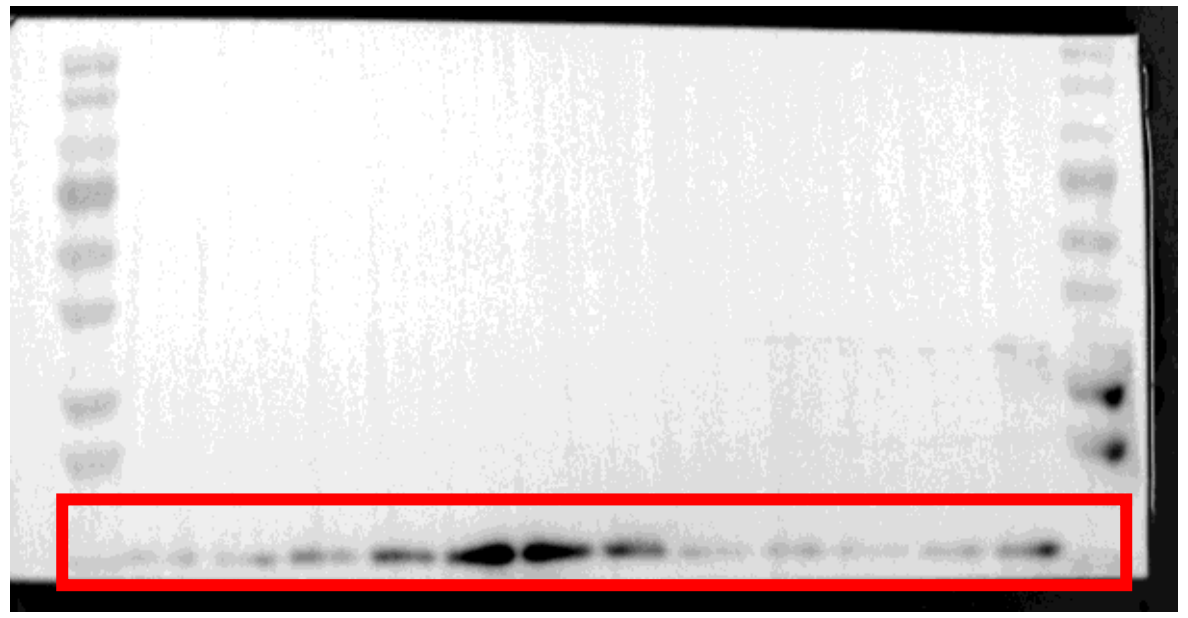

Figure S6f -GPX4-16 kDa

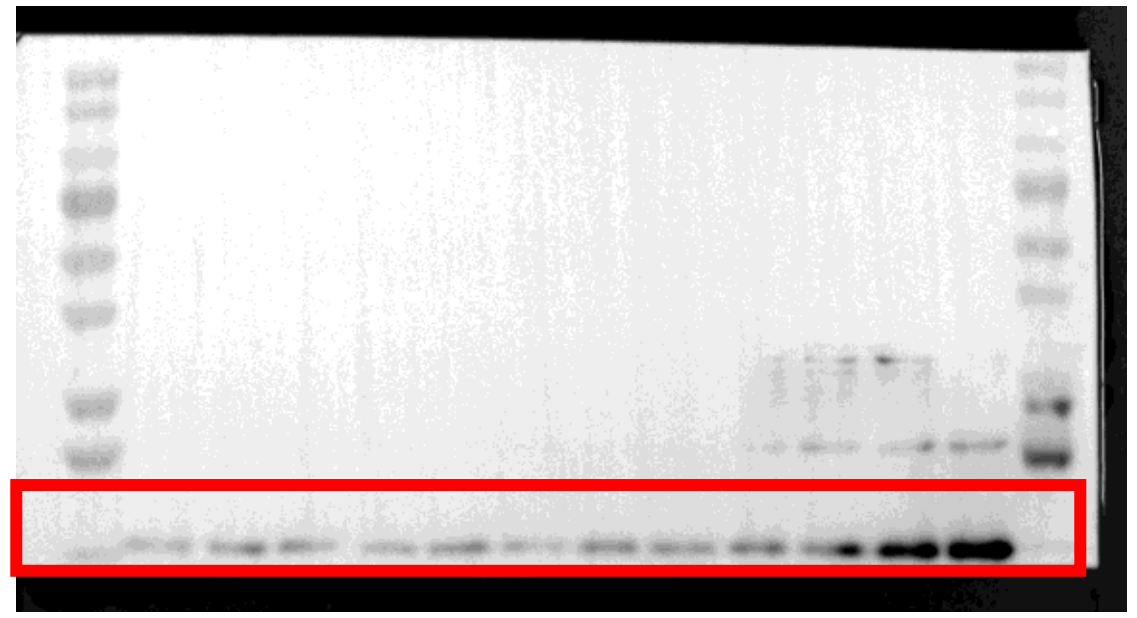

Figure S6f --β-actin--43 kDa

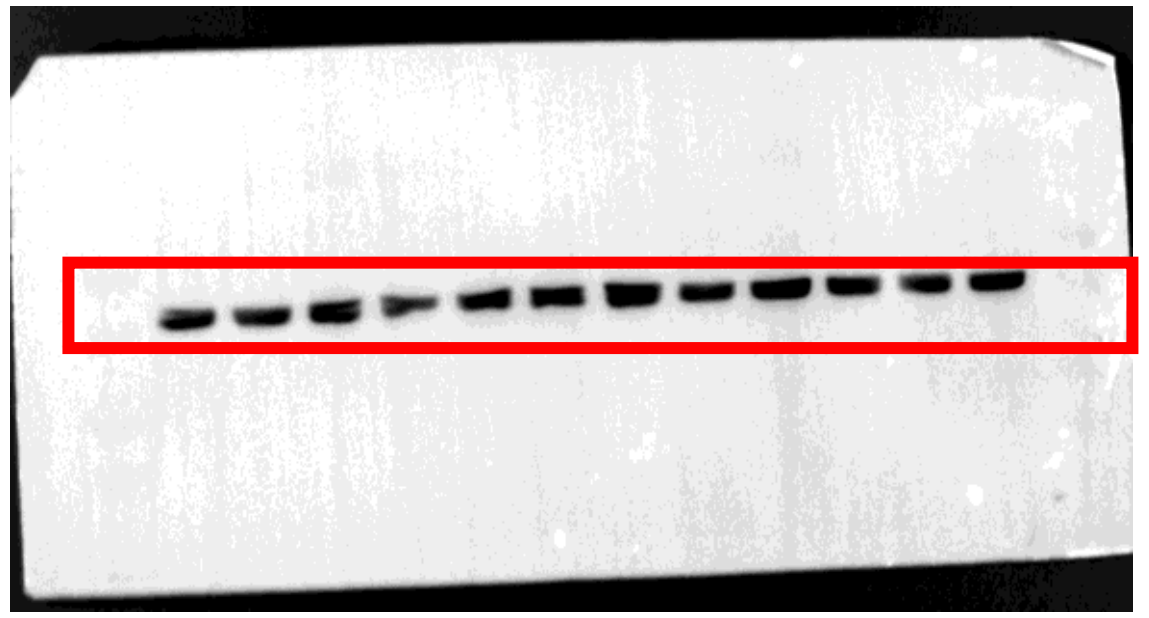

Supplement: Supplementary file 1 [file 41420_2026_3248_MOESM2_ESM.pdf]
